# Datasets for transcriptomics, q-proteomics and phenotype microarrays of polyphosphate metabolism mutants from *Escherichia coli*

**DOI:** 10.1016/j.dib.2017.03.010

**Published:** 2017-03-18

**Authors:** Macarena Varas, Camilo Valdivieso, Cecilia Mauriaca, Javiera Ortíz-Severín, Alberto Paradela, Ignacio Poblete-Castro, Ricardo Cabrera, Francisco P. Chávez

**Affiliations:** a Systems Microbiology Laboratory, Department of Biology, Faculty of Science, University of Chile, Chile; b Department of Ecology, Faculty of Science, University of Chile, Chile; c Proteomic Service, CNB CSIC, Spain; d Facultad de Ciencias Biológicas, Center for Bioinformatics and Integrative Biology, Biosystems Engineering Laboratory, Universidad Andrés Bello, Chile; e Department of Biology, Faculty of Sciences, University of Chile, Chile

**Keywords:** Systems biology, Transcriptomics, Q-proteomics, Phenomics, Polyphosphate

## Abstract

Here, we provide the dataset associated with our research article on the polyphosphate metabolism entitled, “Multi-level evaluation of Escherichia coli polyphosphate related mutants using global transcriptomic, proteomic and phenomic analyses”. By integrating different omics levels (transcriptome, proteome and phenome), we were able to study *Escherichia coli* polyphosphate mutant strains (*Δppk1*, *Δppx*, and *Δppk1-ppx*). We have compiled here all datasets from DNA microarrys, q-proteomic (Isotope-Coded Protein Labeling, ICPL) and phenomic (Phenotype microarray) raw data we have obtained in all polyP metabolism mutants.

**Specifications Table**

**Table:** 

Subject area	*Biology*
More specific subject area	*Microbiology*
Type of data	*Tables and Figures*
How data was acquired	*Functional genomic data were obtained using microarrays, Proteomic data were obtained using Isotope Coded Protein Labeling (ICPL) and Phenomic data were obtained using Biolog Phenotypic microarray*
Data format	*Raw and bioinformatic data*
Experimental factors	*We compare polyphosphate mutant strains (Δppk1, Δppx, and Δppk1-ppx) with the wild type*
Experimental features	*All the strains were growth until mid-exponential phase and were analyzed as described in Materials and Methods*
Data accessibility	*Microarray data are available at* GEO: GSE29954, Q- proteomic raw.baf files are available at http://goo.gl/NrKDCB
	The rest of raw data is included in this article.

**Value of the data**

• Three different omics approaches data are available that provides insights of the polyphosphate metabolism in *Escherichia coli*.
• Functional genomics, quantitative proteomics and phenomics data are useful to identify novel phenotypes and metabolic adjustments during alteration of polyP metabolism.
• This work contributes to the knowledge of bacterial polyphosphate metabolism and will help in designing new and more effective antivirulence toward bacterial pathogens.

## 1 Data

The data provided here represent different omics approaches to contribute to the understanding of the metabolism of the polyphosphates in *Escherichia coli*. The raw data comprise information about differential gene expression, protein abundance and response to different metabolites in three *E. coli* mutants (Δ*ppk1*, Δ*ppx*, and Δ*polyP*) strains related to polyphosphate metabolism. This Data in Brief is associated with the research article in BBA General Subjects entitled “Multi-level evaluation of *Escherichia coli* polyphosphate related mutants using global transcriptomic, proteomic and phenomic analyses” [1].

## 2 Experimental design, materials and methods

Three mutant strains of *E. coli* lacking in the enzymes related to the polyphosphates metabolism were generated *(Δppk1, Δppx, and Δppk1-ppx)*. By different omics approaches (microarrays, quantitative proteomic and phenotypic microarrays) we generated an overview of the metabolism of the polyphosphates in this bacterium. The strains were growth in LB media until mid-exponential phase. Total cells, RNA and proteins were extracted and delivered for their respective analysis. Comparisons among the mutant strains and the wild type were performed at all omics levels.

### 2.1 Transcriptomics analysis using DNA microarray

*E. coli* 50-mer oligonucleotide library from MWGBiotech Oligo Sets (http://www.mwgbiotech.com) were used for microarray analysis as previously described [1].

Acquisition and quantification of microarray images was performed using ScanArray 4000 (Packard BioChips). All images were captured using 65% PMT gain, 70–75% laser power and 10 µm resolution at 50% scan rate.

Microarray data analysis was performed with free software genArise, developed in the Computing Unit of Cellular Physiology Institute of UNAM (http://www.ifc.unam.mx/genarise/). GenArise carry out a number of transformations: background correction, normalization, intensity filter, replicates analysis and selecting differentially expressed genes. The goal of genArise is to identify which genes show good evidence of being differentially expressed. The software identifies differential expressed genes by calculating an intensity-dependent *z*-score. Using a sliding window algorithm to calculate the mean and standard deviation within a window surrounding each data point, and define a *z*-score where *z* measures the number of standard deviations a data is from the mean.

*zi*=(*Ri*–mean(*R*))/sd(*R*), where *zi* is the *z*-score for each element, *Ri* is the log-ratio for each element, and sd(*R*) is the standard deviation of the log-ratio. With this criterion, the elements with a *z*-score>2 standard deviations would be the significantly differentially expressed genes.

The expression data were analyzed using the LIMMA framework in Bioconductor http://www.bioconductor.org. Microarray data is available at the NCBI Gene Expression Omnibus (GEO) database with the GSE29954 as accession number. Appendix file A contained raw files with all differentially expressed genes (DEG).

### 2.2 Q-proteomics analysis using isotope-coded protein label (ICPL)

Total protein extracts preparation for ICPL analysis were prepared as previousy reported [2]. Proteome extracts from three biological replicates (different independent cultures) were mixed using fifty micrograms of each one to obtain a triplicate representative sample of each experimental condition with a total of 150 μg of protein in each case. Total protein was quantified using Bio-Rad Protein Assay^®^ (Bio-Rad, USA) and stored at −20 °C previous to the quantitative proteomics analysis.

ICPL-reagent protocol has been optimized for labeling 100 µg of each individual sample per experiment. Thus, 100 µg of the wild-type and the mutant proteome were individually dissolved in 8 M Urea and 25 mM ammonium bicarbonate, reduced and alkylated with iodoacetamide. Urea concentration was reduced to 2 M and sample digested with trypsin (ratio 20:1) O/N at 37 °C. Digested samples were dried and resuspended in 50 µl of 0.2% trifluoroacetic acid in water and salts and urea removed using high-capacity OMIX C18 tips (Varian, Palo Alto, Ca).

Labeling with the ICPL reagent was performed at the peptide level as previously described [1]. Briefly, 100 µg of each individual proteome sample was individually dissolved in 20 µl of lysis buffer (containing 6 M of guanidinium chloride). ICPL reagent was added to each individual sample and reaction was incubated at 25 °C for 2 h. Collective samples were dried in speed-vac and stored dry at −20 °C until needed. ICPL-labeled combined samples (200 µg per experiment) were fractionated by a 2D-LC approach. Second dimension of the 2D-nano LC ESI-MSMS analyses was performed using an Ultimate 3000 nanoHPLC (Dionex, Sunnyvale, California) coupled to an HCT Ultra ion-trap mass spectrometer (Bruker Daltonics, Bremen, Germany). The LC system was coupled via a nanospray source (Bruker Daltonics, Bremen, Germany) to a 3D ion trap mass spectrometer operating in positive ion mode with the capillary voltage set at 1400 V. Automatic data-dependent acquisition allowed to obtain sequentially both full scan (m/z 350–1500) MS spectra followed by tandem MS CID spectra of the four most abundant ions. Dynamic exclusion was applied to prevent the same *m*/*z* from being isolated for 1 min after its fragmentation.

MS and MS/MS data obtained for individual HPLC fractions were merged using the Analysis Combiner tool and subsequently processed as a single experiment. Merged raw MS and MSMS data were processed using DataAnalysis 3.4 (Bruker Daltonics, Bremen, Germany). For protein identification, the *Escherichia coli* strain K12 database, containing 4407 protein coding genes, was downloaded from the UniProtKB Protein Knowledgebase (http://www.uniprot.org/) [3]. Original.baf files (Bruker Daltonics) for all three mutants are available upon request or available at site http://goo.gl/NrKDCB.

A combined database containing both forward and their corresponding reversed sequences was generated using the program pSCAN (http://pfind.ict.ac.cn). MSMS spectra (in the form of mascot generic files) were searched against this combined database using a licensed version of Mascot v.2.2.04 (www.matrixscience.com; Matrix Science, London, UK). Search parameters were set as follows: carbamidomethyl cystein as fixed modification, oxidized methionines and ICPL- labeling of lysine residues and/or peptide amino termini as variable ones. Peptide mass tolerance was set at 0.6 Da both in MS and MS/MS mode, and 1 missed cleavage was allowed. Typically, an accuracy of ±0.1–0.2 Da was found both for MS and MS/MS spectra. False Discovery Rates (FDR ≤5%) for peptide identification was manually calculated as follows: after database searching, peptide matches with a Mascot score >20 were ordered according to their Mascot scores.

Appendix file B contained the raw datasets with the list contained peptide sequences matching either forward or reversed database sequences. Then, a sublist containing only a 5% of peptides matching the reversed sequences was extracted and used for further analysis. Quantitative analysis was performed using WARP-LC 1.1 (Bruker Daltonics) using the parameters described above. When only one ICPL-labeled peptide was identified, the extracted ion chromatogram of its putative partner was used for quantification. Putative ICPL pairs were determined allowing a mass tolerance of 0.5 Da and a retention time tolerance of 40 s. Relative ratios between the light and heavy counterparts of ICPL-labeled peptides were calculated using the intensity of their respective monoisotopic peaks. Except where indicated otherwise, only proteins identified and quantified with at least two peptides were considered. Ratios were log2-transformed and normalized by subtracting the median value.

### 2.3 Phenomic analysis using phenotypic microarrays

Bacterial cell suspensions from *E. coli* polyphosphate metabolism mutant strains were inoculated into each of the 20 PM plates for full metabolic profiling according to standard protocols recommended by Biolog Inc [4]. *E. coli* K12 was used as parental control strains in all experiments. The PM plates (complete set) were located in an aerobic OmniLog incubator reader set at 30 °C which collected data every 15 min over a 72-h period. PM tests were conducted in duplicate, and the plates were also examined visually at the end of each incubation period for independent confirmation. The OmniLog^®^ V. 1.5 comparison module and the average height parameter were used for data analysis with standard thresholds for detection. A consensus graphical profiles for all metabolic and sensitivity tests for each mutant were generated using two independent runs as previously reported [5]. Original raw data from all polyP mutants strains is available for each polyP metabolism mutant in Appendix file C.

## Appendix A Supplementary material

.

Raw data from microarray experiments (.docx files).

.

Raw data from ICPL proteomic experiments (.xls files).

.

Raw data from phenotypic microarray experiments (.docx files).
