# Supplementary material for: Datasets for transcriptomics, q-proteomics and phenotype microarrays of polyphosphate metabolism mutants from Escherichia coli
Source: Data Brief. 2017 Mar 18;12:13–7. doi: 10.1016/j.dib.2017.03.010 (PMC5367803; doi:10.1016/j.dib.2017.03.010)
Supplement: Supplementary file 2 — Supplementary material [file mmc2.zip › BBAGEN-16-480/Wt vs polyP marrays.docx]

| **ID** | **Symdesc** | **Zscore** |
| --- | --- | --- |
| [murB](http://www.ncbi.nlm.nih.gov/entrez/query.fcgi?CMD=search&DB=gene&term=MURB) | [ECK3964](http://www.ncbi.nlm.nih.gov/entrez/query.fcgi?CMD=search&DB=gene&term=ECK3964) | 2.734929 |
| [lon](http://www.ncbi.nlm.nih.gov/entrez/query.fcgi?CMD=search&DB=gene&term=LON) | [ECK0433](http://www.ncbi.nlm.nih.gov/entrez/query.fcgi?CMD=search&DB=gene&term=ECK0433) | 2.426448 |
| [deaD-R](http://www.ncbi.nlm.nih.gov/entrez/query.fcgi?CMD=search&DB=gene&term=DEAD-R) | [ECK3150](http://www.ncbi.nlm.nih.gov/entrez/query.fcgi?CMD=search&DB=gene&term=ECK3150) | 3.221758 |
| [deaD](http://www.ncbi.nlm.nih.gov/entrez/query.fcgi?CMD=search&DB=gene&term=DEAD) | [ECK3150](http://www.ncbi.nlm.nih.gov/entrez/query.fcgi?CMD=search&DB=gene&term=ECK3150) | 2.349982 |
| [yhiX](http://www.ncbi.nlm.nih.gov/entrez/query.fcgi?CMD=search&DB=gene&term=YHIX) | [ECK3501](http://www.ncbi.nlm.nih.gov/entrez/query.fcgi?CMD=search&DB=gene&term=ECK3501) | 3.235002 |
| [yeaJ](http://www.ncbi.nlm.nih.gov/entrez/query.fcgi?CMD=search&DB=gene&term=YEAJ) | [ECK1784](http://www.ncbi.nlm.nih.gov/entrez/query.fcgi?CMD=search&DB=gene&term=ECK1784) | 1.593704 |
| [yjfC](http://www.ncbi.nlm.nih.gov/entrez/query.fcgi?CMD=search&DB=gene&term=YJFC) | [ECK4182](http://www.ncbi.nlm.nih.gov/entrez/query.fcgi?CMD=search&DB=gene&term=ECK4182) | 1.654162 |
| [yhbJ](http://www.ncbi.nlm.nih.gov/entrez/query.fcgi?CMD=search&DB=gene&term=YHBJ) | [ECK3194](http://www.ncbi.nlm.nih.gov/entrez/query.fcgi?CMD=search&DB=gene&term=ECK3194) | 2.816409 |
| [yjhE](http://www.ncbi.nlm.nih.gov/entrez/query.fcgi?CMD=search&DB=gene&term=YJHE) | [ECK4272](http://www.ncbi.nlm.nih.gov/entrez/query.fcgi?CMD=search&DB=gene&term=ECK4272) | 4.719322 |
| [yhdA](http://www.ncbi.nlm.nih.gov/entrez/query.fcgi?CMD=search&DB=gene&term=YHDA) | [ECK3240](http://www.ncbi.nlm.nih.gov/entrez/query.fcgi?CMD=search&DB=gene&term=ECK3240) | 1.501065 |
| [pfkA](http://www.ncbi.nlm.nih.gov/entrez/query.fcgi?CMD=search&DB=gene&term=PFKA) | [ECK3908](http://www.ncbi.nlm.nih.gov/entrez/query.fcgi?CMD=search&DB=gene&term=ECK3908) | 1.640363 |
| [yciW](http://www.ncbi.nlm.nih.gov/entrez/query.fcgi?CMD=search&DB=gene&term=YCIW) | [ECK1282](http://www.ncbi.nlm.nih.gov/entrez/query.fcgi?CMD=search&DB=gene&term=ECK1282) | 1.504698 |
| [ftsJ](http://www.ncbi.nlm.nih.gov/entrez/query.fcgi?CMD=search&DB=gene&term=FTSJ) | [ECK3168](http://www.ncbi.nlm.nih.gov/entrez/query.fcgi?CMD=search&DB=gene&term=ECK3168) | 1.863759 |
| [tdcB](http://www.ncbi.nlm.nih.gov/entrez/query.fcgi?CMD=search&DB=gene&term=TDCB) | [ECK3106](http://www.ncbi.nlm.nih.gov/entrez/query.fcgi?CMD=search&DB=gene&term=ECK3106) | 2.987604 |
| [B1438](http://www.ncbi.nlm.nih.gov/entrez/query.fcgi?CMD=search&DB=gene&term=B1438) | [ECK1432](http://www.ncbi.nlm.nih.gov/entrez/query.fcgi?CMD=search&DB=gene&term=ECK1432) | 3.429457 |
| [yccD](http://www.ncbi.nlm.nih.gov/entrez/query.fcgi?CMD=search&DB=gene&term=YCCD) | [ECK0990](http://www.ncbi.nlm.nih.gov/entrez/query.fcgi?CMD=search&DB=gene&term=ECK0990) | 2.182722 |
| [B1680](http://www.ncbi.nlm.nih.gov/entrez/query.fcgi?CMD=search&DB=gene&term=B1680) | [ECK1676](http://www.ncbi.nlm.nih.gov/entrez/query.fcgi?CMD=search&DB=gene&term=ECK1676) | 2.231679 |
| [ybaD](http://www.ncbi.nlm.nih.gov/entrez/query.fcgi?CMD=search&DB=gene&term=YBAD) | [ECK0407](http://www.ncbi.nlm.nih.gov/entrez/query.fcgi?CMD=search&DB=gene&term=ECK0407) | 2.128087 |
| [talA](http://www.ncbi.nlm.nih.gov/entrez/query.fcgi?CMD=search&DB=gene&term=TALA) | [ECK2459](http://www.ncbi.nlm.nih.gov/entrez/query.fcgi?CMD=search&DB=gene&term=ECK2459) | 2.039603 |
| [ispA](http://www.ncbi.nlm.nih.gov/entrez/query.fcgi?CMD=search&DB=gene&term=ISPA) | [ECK0415](http://www.ncbi.nlm.nih.gov/entrez/query.fcgi?CMD=search&DB=gene&term=ECK0415) | 1.597783 |
| [ybeZ](http://www.ncbi.nlm.nih.gov/entrez/query.fcgi?CMD=search&DB=gene&term=YBEZ) | [ECK0652](http://www.ncbi.nlm.nih.gov/entrez/query.fcgi?CMD=search&DB=gene&term=ECK0652) | 2.108466 |
| [yhiE](http://www.ncbi.nlm.nih.gov/entrez/query.fcgi?CMD=search&DB=gene&term=YHIE) | [ECK3496](http://www.ncbi.nlm.nih.gov/entrez/query.fcgi?CMD=search&DB=gene&term=ECK3496) | 4.997463 |
| [hdeD](http://www.ncbi.nlm.nih.gov/entrez/query.fcgi?CMD=search&DB=gene&term=HDED) | [ECK3495](http://www.ncbi.nlm.nih.gov/entrez/query.fcgi?CMD=search&DB=gene&term=ECK3495) | 1.748362 |
| [hslV](http://www.ncbi.nlm.nih.gov/entrez/query.fcgi?CMD=search&DB=gene&term=HSLV) | [ECK3924](http://www.ncbi.nlm.nih.gov/entrez/query.fcgi?CMD=search&DB=gene&term=ECK3924) | 1.711615 |
| [B2097](http://www.ncbi.nlm.nih.gov/entrez/query.fcgi?CMD=search&DB=gene&term=B2097) | [ECK2090](http://www.ncbi.nlm.nih.gov/entrez/query.fcgi?CMD=search&DB=gene&term=ECK2090) | 2.142016 |
| [polB](http://www.ncbi.nlm.nih.gov/entrez/query.fcgi?CMD=search&DB=gene&term=POLB) | [ECK0061](http://www.ncbi.nlm.nih.gov/entrez/query.fcgi?CMD=search&DB=gene&term=ECK0061) | 5.394783 |
| [gapC_1](http://www.ncbi.nlm.nih.gov/entrez/query.fcgi?CMD=search&DB=gene&term=GAPC_1) | [ECK1409](http://www.ncbi.nlm.nih.gov/entrez/query.fcgi?CMD=search&DB=gene&term=ECK1409) | 3.836414 |
| [B1297](http://www.ncbi.nlm.nih.gov/entrez/query.fcgi?CMD=search&DB=gene&term=B1297) | [ECK1292](http://www.ncbi.nlm.nih.gov/entrez/query.fcgi?CMD=search&DB=gene&term=ECK1292) | 2.573410 |
| [ybeV](http://www.ncbi.nlm.nih.gov/entrez/query.fcgi?CMD=search&DB=gene&term=YBEV) | [ECK0642](http://www.ncbi.nlm.nih.gov/entrez/query.fcgi?CMD=search&DB=gene&term=ECK0642) | 3.241058 |
| [yihK](http://www.ncbi.nlm.nih.gov/entrez/query.fcgi?CMD=search&DB=gene&term=YIHK) | [ECK3864](http://www.ncbi.nlm.nih.gov/entrez/query.fcgi?CMD=search&DB=gene&term=ECK3864) | 2.778838 |
| [B2228](http://www.ncbi.nlm.nih.gov/entrez/query.fcgi?CMD=search&DB=gene&term=B2228) | [ECK2220](http://www.ncbi.nlm.nih.gov/entrez/query.fcgi?CMD=search&DB=gene&term=ECK2220) | 2.109394 |
| [B2383](http://www.ncbi.nlm.nih.gov/entrez/query.fcgi?CMD=search&DB=gene&term=B2383) | [ECK2379](http://www.ncbi.nlm.nih.gov/entrez/query.fcgi?CMD=search&DB=gene&term=ECK2379) | 2.098338 |
| [hdeB](http://www.ncbi.nlm.nih.gov/entrez/query.fcgi?CMD=search&DB=gene&term=HDEB) | [ECK3493](http://www.ncbi.nlm.nih.gov/entrez/query.fcgi?CMD=search&DB=gene&term=ECK3493) | 5.649414 |
| [prpE](http://www.ncbi.nlm.nih.gov/entrez/query.fcgi?CMD=search&DB=gene&term=PRPE) | [ECK0332](http://www.ncbi.nlm.nih.gov/entrez/query.fcgi?CMD=search&DB=gene&term=ECK0332) | 2.000908 |
| [B1806](http://www.ncbi.nlm.nih.gov/entrez/query.fcgi?CMD=search&DB=gene&term=B1806) | [ECK1804](http://www.ncbi.nlm.nih.gov/entrez/query.fcgi?CMD=search&DB=gene&term=ECK1804) | 3.042599 |
| [ybjE](http://www.ncbi.nlm.nih.gov/entrez/query.fcgi?CMD=search&DB=gene&term=YBJE) | [ECK0865](http://www.ncbi.nlm.nih.gov/entrez/query.fcgi?CMD=search&DB=gene&term=ECK0865) | 1.533201 |
| [rplT](http://www.ncbi.nlm.nih.gov/entrez/query.fcgi?CMD=search&DB=gene&term=RPLT) | [ECK1714](http://www.ncbi.nlm.nih.gov/entrez/query.fcgi?CMD=search&DB=gene&term=ECK1714) | 1.996126 |
| [yceD](http://www.ncbi.nlm.nih.gov/entrez/query.fcgi?CMD=search&DB=gene&term=YCED) | [ECK1074](http://www.ncbi.nlm.nih.gov/entrez/query.fcgi?CMD=search&DB=gene&term=ECK1074) | 1.965143 |
| [wecC](http://www.ncbi.nlm.nih.gov/entrez/query.fcgi?CMD=search&DB=gene&term=WECC) | [ECK3779](http://www.ncbi.nlm.nih.gov/entrez/query.fcgi?CMD=search&DB=gene&term=ECK3779) | 1.782464 |
| [nadC](http://www.ncbi.nlm.nih.gov/entrez/query.fcgi?CMD=search&DB=gene&term=NADC) | [ECK0108](http://www.ncbi.nlm.nih.gov/entrez/query.fcgi?CMD=search&DB=gene&term=ECK0108) | 1.817309 |
| [himA](http://www.ncbi.nlm.nih.gov/entrez/query.fcgi?CMD=search&DB=gene&term=HIMA) | [ECK1710](http://www.ncbi.nlm.nih.gov/entrez/query.fcgi?CMD=search&DB=gene&term=ECK1710) | 3.414050 |
| [rpsM](http://www.ncbi.nlm.nih.gov/entrez/query.fcgi?CMD=search&DB=gene&term=RPSM) | [ECK3285](http://www.ncbi.nlm.nih.gov/entrez/query.fcgi?CMD=search&DB=gene&term=ECK3285) | 1.966231 |
| [mdaA](http://www.ncbi.nlm.nih.gov/entrez/query.fcgi?CMD=search&DB=gene&term=MDAA) | [ECK0842](http://www.ncbi.nlm.nih.gov/entrez/query.fcgi?CMD=search&DB=gene&term=ECK0842) | 1.584968 |
| [ybjZ](http://www.ncbi.nlm.nih.gov/entrez/query.fcgi?CMD=search&DB=gene&term=YBJZ) | [ECK0870](http://www.ncbi.nlm.nih.gov/entrez/query.fcgi?CMD=search&DB=gene&term=ECK0870) | 2.449408 |
| [bglG](http://www.ncbi.nlm.nih.gov/entrez/query.fcgi?CMD=search&DB=gene&term=BGLG) | [ECK3716](http://www.ncbi.nlm.nih.gov/entrez/query.fcgi?CMD=search&DB=gene&term=ECK3716) | 1.887348 |
| [aroG](http://www.ncbi.nlm.nih.gov/entrez/query.fcgi?CMD=search&DB=gene&term=AROG) | [ECK0743](http://www.ncbi.nlm.nih.gov/entrez/query.fcgi?CMD=search&DB=gene&term=ECK0743) | 2.013484 |
| [proS](http://www.ncbi.nlm.nih.gov/entrez/query.fcgi?CMD=search&DB=gene&term=PROS) | [ECK0194](http://www.ncbi.nlm.nih.gov/entrez/query.fcgi?CMD=search&DB=gene&term=ECK0194) | 1.691083 |
| [acrF](http://www.ncbi.nlm.nih.gov/entrez/query.fcgi?CMD=search&DB=gene&term=ACRF) | [ECK3253](http://www.ncbi.nlm.nih.gov/entrez/query.fcgi?CMD=search&DB=gene&term=ECK3253) | 1.844326 |
| [rus-R](http://www.ncbi.nlm.nih.gov/entrez/query.fcgi?CMD=search&DB=gene&term=RUS-R) | [ECK0541](http://www.ncbi.nlm.nih.gov/entrez/query.fcgi?CMD=search&DB=gene&term=ECK0541) | 1.893409 |
| [yjgL](http://www.ncbi.nlm.nih.gov/entrez/query.fcgi?CMD=search&DB=gene&term=YJGL) | [ECK4246](http://www.ncbi.nlm.nih.gov/entrez/query.fcgi?CMD=search&DB=gene&term=ECK4246) | 1.630450 |
| [rfaI](http://www.ncbi.nlm.nih.gov/entrez/query.fcgi?CMD=search&DB=gene&term=RFAI) | [ECK3617](http://www.ncbi.nlm.nih.gov/entrez/query.fcgi?CMD=search&DB=gene&term=ECK3617) | 1.893638 |
| [yabK](http://www.ncbi.nlm.nih.gov/entrez/query.fcgi?CMD=search&DB=gene&term=YABK) | [ECK0068](http://www.ncbi.nlm.nih.gov/entrez/query.fcgi?CMD=search&DB=gene&term=ECK0068) | 1.712494 |
| [minC](http://www.ncbi.nlm.nih.gov/entrez/query.fcgi?CMD=search&DB=gene&term=MINC) | [ECK1164](http://www.ncbi.nlm.nih.gov/entrez/query.fcgi?CMD=search&DB=gene&term=ECK1164) | 1.582556 |
| [fixC](http://www.ncbi.nlm.nih.gov/entrez/query.fcgi?CMD=search&DB=gene&term=FIXC) | [ECK0044](http://www.ncbi.nlm.nih.gov/entrez/query.fcgi?CMD=search&DB=gene&term=ECK0044) | 2.251067 |
| [ybeF](http://www.ncbi.nlm.nih.gov/entrez/query.fcgi?CMD=search&DB=gene&term=YBEF) | [ECK0622](http://www.ncbi.nlm.nih.gov/entrez/query.fcgi?CMD=search&DB=gene&term=ECK0622) | 1.992114 |
| [hpt](http://www.ncbi.nlm.nih.gov/entrez/query.fcgi?CMD=search&DB=gene&term=HPT) | [ECK0124](http://www.ncbi.nlm.nih.gov/entrez/query.fcgi?CMD=search&DB=gene&term=ECK0124) | 1.983425 |
| [xasA](http://www.ncbi.nlm.nih.gov/entrez/query.fcgi?CMD=search&DB=gene&term=XASA) | [ECK1486](http://www.ncbi.nlm.nih.gov/entrez/query.fcgi?CMD=search&DB=gene&term=ECK1486) | 5.407986 |
| [mraY](http://www.ncbi.nlm.nih.gov/entrez/query.fcgi?CMD=search&DB=gene&term=MRAY) | [ECK0088](http://www.ncbi.nlm.nih.gov/entrez/query.fcgi?CMD=search&DB=gene&term=ECK0088) | 1.756673 |
| [nuoB](http://www.ncbi.nlm.nih.gov/entrez/query.fcgi?CMD=search&DB=gene&term=NUOB) | [ECK2281](http://www.ncbi.nlm.nih.gov/entrez/query.fcgi?CMD=search&DB=gene&term=ECK2281) | 3.353278 |
| [yfeU](http://www.ncbi.nlm.nih.gov/entrez/query.fcgi?CMD=search&DB=gene&term=YFEU) | [ECK2423](http://www.ncbi.nlm.nih.gov/entrez/query.fcgi?CMD=search&DB=gene&term=ECK2423) | 2.165240 |
| [ynfM](http://www.ncbi.nlm.nih.gov/entrez/query.fcgi?CMD=search&DB=gene&term=YNFM) | [ECK1591](http://www.ncbi.nlm.nih.gov/entrez/query.fcgi?CMD=search&DB=gene&term=ECK1591) | 1.816201 |
| [cspI](http://www.ncbi.nlm.nih.gov/entrez/query.fcgi?CMD=search&DB=gene&term=CSPI) | [ECK1546](http://www.ncbi.nlm.nih.gov/entrez/query.fcgi?CMD=search&DB=gene&term=ECK1546) | 4.383484 |
| [psiF](http://www.ncbi.nlm.nih.gov/entrez/query.fcgi?CMD=search&DB=gene&term=PSIF) | [ECK0379](http://www.ncbi.nlm.nih.gov/entrez/query.fcgi?CMD=search&DB=gene&term=ECK0379) | 2.476565 |
| [ycaO](http://www.ncbi.nlm.nih.gov/entrez/query.fcgi?CMD=search&DB=gene&term=YCAO) | [ECK0896](http://www.ncbi.nlm.nih.gov/entrez/query.fcgi?CMD=search&DB=gene&term=ECK0896) | 1.616292 |
| [ascF](http://www.ncbi.nlm.nih.gov/entrez/query.fcgi?CMD=search&DB=gene&term=ASCF) | [ECK2710](http://www.ncbi.nlm.nih.gov/entrez/query.fcgi?CMD=search&DB=gene&term=ECK2710) | 1.664356 |
| [yhjU](http://www.ncbi.nlm.nih.gov/entrez/query.fcgi?CMD=search&DB=gene&term=YHJU) | [ECK3523](http://www.ncbi.nlm.nih.gov/entrez/query.fcgi?CMD=search&DB=gene&term=ECK3523) | 2.410678 |
| [ybdJ](http://www.ncbi.nlm.nih.gov/entrez/query.fcgi?CMD=search&DB=gene&term=YBDJ) | [ECK0572](http://www.ncbi.nlm.nih.gov/entrez/query.fcgi?CMD=search&DB=gene&term=ECK0572) | 2.130160 |
| [ybbY](http://www.ncbi.nlm.nih.gov/entrez/query.fcgi?CMD=search&DB=gene&term=YBBY) | [ECK0506](http://www.ncbi.nlm.nih.gov/entrez/query.fcgi?CMD=search&DB=gene&term=ECK0506) | 2.901607 |
| [lplA](http://www.ncbi.nlm.nih.gov/entrez/query.fcgi?CMD=search&DB=gene&term=LPLA) | [ECK4378](http://www.ncbi.nlm.nih.gov/entrez/query.fcgi?CMD=search&DB=gene&term=ECK4378) | 2.917130 |
| [B1485](http://www.ncbi.nlm.nih.gov/entrez/query.fcgi?CMD=search&DB=gene&term=B1485) | [ECK1479](http://www.ncbi.nlm.nih.gov/entrez/query.fcgi?CMD=search&DB=gene&term=ECK1479) | 1.799731 |
| [ugpA](http://www.ncbi.nlm.nih.gov/entrez/query.fcgi?CMD=search&DB=gene&term=UGPA) | [ECK3436](http://www.ncbi.nlm.nih.gov/entrez/query.fcgi?CMD=search&DB=gene&term=ECK3436) | 1.769857 |
| [uspA](http://www.ncbi.nlm.nih.gov/entrez/query.fcgi?CMD=search&DB=gene&term=USPA) | [ECK3480](http://www.ncbi.nlm.nih.gov/entrez/query.fcgi?CMD=search&DB=gene&term=ECK3480) | 1.920082 |
| [ydiC](http://www.ncbi.nlm.nih.gov/entrez/query.fcgi?CMD=search&DB=gene&term=YDIC) | [ECK1680](http://www.ncbi.nlm.nih.gov/entrez/query.fcgi?CMD=search&DB=gene&term=ECK1680) | 1.654258 |
| [B1644](http://www.ncbi.nlm.nih.gov/entrez/query.fcgi?CMD=search&DB=gene&term=B1644) | [ECK1640](http://www.ncbi.nlm.nih.gov/entrez/query.fcgi?CMD=search&DB=gene&term=ECK1640) | 1.591223 |
| [ykfF](http://www.ncbi.nlm.nih.gov/entrez/query.fcgi?CMD=search&DB=gene&term=YKFF) | [ECK0251](http://www.ncbi.nlm.nih.gov/entrez/query.fcgi?CMD=search&DB=gene&term=ECK0251) | 1.702792 |
| [yadH](http://www.ncbi.nlm.nih.gov/entrez/query.fcgi?CMD=search&DB=gene&term=YADH) | [ECK0127](http://www.ncbi.nlm.nih.gov/entrez/query.fcgi?CMD=search&DB=gene&term=ECK0127) | 2.793199 |
| [rffH](http://www.ncbi.nlm.nih.gov/entrez/query.fcgi?CMD=search&DB=gene&term=RFFH) | [ECK3781](http://www.ncbi.nlm.nih.gov/entrez/query.fcgi?CMD=search&DB=gene&term=ECK3781) | 1.621781 |
| [ygcA](http://www.ncbi.nlm.nih.gov/entrez/query.fcgi?CMD=search&DB=gene&term=YGCA) | [ECK2779](http://www.ncbi.nlm.nih.gov/entrez/query.fcgi?CMD=search&DB=gene&term=ECK2779) | 1.659322 |
| [B1560](http://www.ncbi.nlm.nih.gov/entrez/query.fcgi?CMD=search&DB=gene&term=B1560) | [ECK1554](http://www.ncbi.nlm.nih.gov/entrez/query.fcgi?CMD=search&DB=gene&term=ECK1554) | 1.709562 |
| [B2790](http://www.ncbi.nlm.nih.gov/entrez/query.fcgi?CMD=search&DB=gene&term=B2790) | [ECK2784](http://www.ncbi.nlm.nih.gov/entrez/query.fcgi?CMD=search&DB=gene&term=ECK2784) | 1.672276 |
| [hdeA](http://www.ncbi.nlm.nih.gov/entrez/query.fcgi?CMD=search&DB=gene&term=HDEA) | [ECK3494](http://www.ncbi.nlm.nih.gov/entrez/query.fcgi?CMD=search&DB=gene&term=ECK3494) | 3.990820 |
| [yjhP](http://www.ncbi.nlm.nih.gov/entrez/query.fcgi?CMD=search&DB=gene&term=YJHP) | [ECK4296](http://www.ncbi.nlm.nih.gov/entrez/query.fcgi?CMD=search&DB=gene&term=ECK4296) | 2.110786 |
| [ytfL](http://www.ncbi.nlm.nih.gov/entrez/query.fcgi?CMD=search&DB=gene&term=YTFL) | [ECK4214](http://www.ncbi.nlm.nih.gov/entrez/query.fcgi?CMD=search&DB=gene&term=ECK4214) | 5.509768 |
| [ndh](http://www.ncbi.nlm.nih.gov/entrez/query.fcgi?CMD=search&DB=gene&term=NDH) | [ECK1095](http://www.ncbi.nlm.nih.gov/entrez/query.fcgi?CMD=search&DB=gene&term=ECK1095) | 1.571376 |
| [guaC](http://www.ncbi.nlm.nih.gov/entrez/query.fcgi?CMD=search&DB=gene&term=GUAC) | [ECK0104](http://www.ncbi.nlm.nih.gov/entrez/query.fcgi?CMD=search&DB=gene&term=ECK0104) | 1.768770 |
| [cybC](http://www.ncbi.nlm.nih.gov/entrez/query.fcgi?CMD=search&DB=gene&term=CYBC) | [ECK4231](http://www.ncbi.nlm.nih.gov/entrez/query.fcgi?CMD=search&DB=gene&term=ECK4231) | 1.825332 |
| [sseB](http://www.ncbi.nlm.nih.gov/entrez/query.fcgi?CMD=search&DB=gene&term=SSEB) | [ECK2519](http://www.ncbi.nlm.nih.gov/entrez/query.fcgi?CMD=search&DB=gene&term=ECK2519) | 2.101563 |
| [ycjV](http://www.ncbi.nlm.nih.gov/entrez/query.fcgi?CMD=search&DB=gene&term=YCJV) | [ECK1313](http://www.ncbi.nlm.nih.gov/entrez/query.fcgi?CMD=search&DB=gene&term=ECK1313) | 2.033674 |
| [yhaR](http://www.ncbi.nlm.nih.gov/entrez/query.fcgi?CMD=search&DB=gene&term=YHAR) | [ECK3102](http://www.ncbi.nlm.nih.gov/entrez/query.fcgi?CMD=search&DB=gene&term=ECK3102) | 2.213044 |
| [gadB](http://www.ncbi.nlm.nih.gov/entrez/query.fcgi?CMD=search&DB=gene&term=GADB) | [ECK1487](http://www.ncbi.nlm.nih.gov/entrez/query.fcgi?CMD=search&DB=gene&term=ECK1487) | 5.660959 |
| [kdsA](http://www.ncbi.nlm.nih.gov/entrez/query.fcgi?CMD=search&DB=gene&term=KDSA) | [ECK1203](http://www.ncbi.nlm.nih.gov/entrez/query.fcgi?CMD=search&DB=gene&term=ECK1203) | 1.987355 |
| [yjaH](http://www.ncbi.nlm.nih.gov/entrez/query.fcgi?CMD=search&DB=gene&term=YJAH) | [ECK3993](http://www.ncbi.nlm.nih.gov/entrez/query.fcgi?CMD=search&DB=gene&term=ECK3993) | 2.014322 |
| [rpoB](http://www.ncbi.nlm.nih.gov/entrez/query.fcgi?CMD=search&DB=gene&term=RPOB) | [ECK3978](http://www.ncbi.nlm.nih.gov/entrez/query.fcgi?CMD=search&DB=gene&term=ECK3978) | 1.991817 |
| [cycA](http://www.ncbi.nlm.nih.gov/entrez/query.fcgi?CMD=search&DB=gene&term=CYCA) | [ECK4204](http://www.ncbi.nlm.nih.gov/entrez/query.fcgi?CMD=search&DB=gene&term=ECK4204) | 2.246404 |
| [yjbJ](http://www.ncbi.nlm.nih.gov/entrez/query.fcgi?CMD=search&DB=gene&term=YJBJ) | [ECK4037](http://www.ncbi.nlm.nih.gov/entrez/query.fcgi?CMD=search&DB=gene&term=ECK4037) | 2.034005 |
| [ygcY](http://www.ncbi.nlm.nih.gov/entrez/query.fcgi?CMD=search&DB=gene&term=YGCY) | [ECK2782](http://www.ncbi.nlm.nih.gov/entrez/query.fcgi?CMD=search&DB=gene&term=ECK2782) | 1.695113 |
| [dksA](http://www.ncbi.nlm.nih.gov/entrez/query.fcgi?CMD=search&DB=gene&term=DKSA) | [ECK0144](http://www.ncbi.nlm.nih.gov/entrez/query.fcgi?CMD=search&DB=gene&term=ECK0144) | 2.956851 |
| [gntP](http://www.ncbi.nlm.nih.gov/entrez/query.fcgi?CMD=search&DB=gene&term=GNTP) | [ECK4312](http://www.ncbi.nlm.nih.gov/entrez/query.fcgi?CMD=search&DB=gene&term=ECK4312) | 2.433331 |
| [hemD](http://www.ncbi.nlm.nih.gov/entrez/query.fcgi?CMD=search&DB=gene&term=HEMD) | [ECK3798](http://www.ncbi.nlm.nih.gov/entrez/query.fcgi?CMD=search&DB=gene&term=ECK3798) | 2.676993 |
| [pepB](http://www.ncbi.nlm.nih.gov/entrez/query.fcgi?CMD=search&DB=gene&term=PEPB) | [ECK2520](http://www.ncbi.nlm.nih.gov/entrez/query.fcgi?CMD=search&DB=gene&term=ECK2520) | 2.094795 |
| [ycaC](http://www.ncbi.nlm.nih.gov/entrez/query.fcgi?CMD=search&DB=gene&term=YCAC) | [ECK0888](http://www.ncbi.nlm.nih.gov/entrez/query.fcgi?CMD=search&DB=gene&term=ECK0888) | 1.610546 |
| [folA](http://www.ncbi.nlm.nih.gov/entrez/query.fcgi?CMD=search&DB=gene&term=FOLA) | [ECK0049](http://www.ncbi.nlm.nih.gov/entrez/query.fcgi?CMD=search&DB=gene&term=ECK0049) | 1.524983 |
| [dnaJ](http://www.ncbi.nlm.nih.gov/entrez/query.fcgi?CMD=search&DB=gene&term=DNAJ) | [ECK0015](http://www.ncbi.nlm.nih.gov/entrez/query.fcgi?CMD=search&DB=gene&term=ECK0015) | 1.528563 |
| [cspC](http://www.ncbi.nlm.nih.gov/entrez/query.fcgi?CMD=search&DB=gene&term=CSPC) | [ECK1821](http://www.ncbi.nlm.nih.gov/entrez/query.fcgi?CMD=search&DB=gene&term=ECK1821) | 2.877364 |
| [dacB](http://www.ncbi.nlm.nih.gov/entrez/query.fcgi?CMD=search&DB=gene&term=DACB) | [ECK3171](http://www.ncbi.nlm.nih.gov/entrez/query.fcgi?CMD=search&DB=gene&term=ECK3171) | 1.528532 |
| [tolA](http://www.ncbi.nlm.nih.gov/entrez/query.fcgi?CMD=search&DB=gene&term=TOLA) | [ECK0728](http://www.ncbi.nlm.nih.gov/entrez/query.fcgi?CMD=search&DB=gene&term=ECK0728) | 3.782478 |
| [yahA](http://www.ncbi.nlm.nih.gov/entrez/query.fcgi?CMD=search&DB=gene&term=YAHA) | [ECK0313](http://www.ncbi.nlm.nih.gov/entrez/query.fcgi?CMD=search&DB=gene&term=ECK0313) | 1.686329 |
| [yjgB](http://www.ncbi.nlm.nih.gov/entrez/query.fcgi?CMD=search&DB=gene&term=YJGB) | [ECK4262](http://www.ncbi.nlm.nih.gov/entrez/query.fcgi?CMD=search&DB=gene&term=ECK4262) | 1.636967 |
| [cspG](http://www.ncbi.nlm.nih.gov/entrez/query.fcgi?CMD=search&DB=gene&term=CSPG) | [ECK0980](http://www.ncbi.nlm.nih.gov/entrez/query.fcgi?CMD=search&DB=gene&term=ECK0980) | 4.134070 |
| [hupB](http://www.ncbi.nlm.nih.gov/entrez/query.fcgi?CMD=search&DB=gene&term=HUPB) | [ECK0434](http://www.ncbi.nlm.nih.gov/entrez/query.fcgi?CMD=search&DB=gene&term=ECK0434) | 1.516099 |
| [sucD](http://www.ncbi.nlm.nih.gov/entrez/query.fcgi?CMD=search&DB=gene&term=SUCD) | [ECK0717](http://www.ncbi.nlm.nih.gov/entrez/query.fcgi?CMD=search&DB=gene&term=ECK0717) | 1.600132 |
| [ybbD](http://www.ncbi.nlm.nih.gov/entrez/query.fcgi?CMD=search&DB=gene&term=YBBD) | [ECK0494](http://www.ncbi.nlm.nih.gov/entrez/query.fcgi?CMD=search&DB=gene&term=ECK0494) | 1.788906 |
| [ttdA](http://www.ncbi.nlm.nih.gov/entrez/query.fcgi?CMD=search&DB=gene&term=TTDA) | [ECK3051](http://www.ncbi.nlm.nih.gov/entrez/query.fcgi?CMD=search&DB=gene&term=ECK3051) | 1.543455 |
| [mhpB](http://www.ncbi.nlm.nih.gov/entrez/query.fcgi?CMD=search&DB=gene&term=MHPB) | [ECK0345](http://www.ncbi.nlm.nih.gov/entrez/query.fcgi?CMD=search&DB=gene&term=ECK0345) | 2.253652 |
| [atpF](http://www.ncbi.nlm.nih.gov/entrez/query.fcgi?CMD=search&DB=gene&term=ATPF) | [ECK3729](http://www.ncbi.nlm.nih.gov/entrez/query.fcgi?CMD=search&DB=gene&term=ECK3729) | 2.213623 |
| [nrfA](http://www.ncbi.nlm.nih.gov/entrez/query.fcgi?CMD=search&DB=gene&term=NRFA) | [ECK4063](http://www.ncbi.nlm.nih.gov/entrez/query.fcgi?CMD=search&DB=gene&term=ECK4063) | 1.539597 |
| [B1541](http://www.ncbi.nlm.nih.gov/entrez/query.fcgi?CMD=search&DB=gene&term=B1541) | [ECK1534](http://www.ncbi.nlm.nih.gov/entrez/query.fcgi?CMD=search&DB=gene&term=ECK1534) | 1.548987 |
| [dcuB](http://www.ncbi.nlm.nih.gov/entrez/query.fcgi?CMD=search&DB=gene&term=DCUB) | [ECK4116](http://www.ncbi.nlm.nih.gov/entrez/query.fcgi?CMD=search&DB=gene&term=ECK4116) | 1.592547 |
| [yfjT](http://www.ncbi.nlm.nih.gov/entrez/query.fcgi?CMD=search&DB=gene&term=YFJT) | [ECK2633](http://www.ncbi.nlm.nih.gov/entrez/query.fcgi?CMD=search&DB=gene&term=ECK2633) | 2.230162 |
| [B1543](http://www.ncbi.nlm.nih.gov/entrez/query.fcgi?CMD=search&DB=gene&term=B1543) | [ECK1536](http://www.ncbi.nlm.nih.gov/entrez/query.fcgi?CMD=search&DB=gene&term=ECK1536) | 1.723875 |
| [fdhD](http://www.ncbi.nlm.nih.gov/entrez/query.fcgi?CMD=search&DB=gene&term=FDHD) | [ECK3888](http://www.ncbi.nlm.nih.gov/entrez/query.fcgi?CMD=search&DB=gene&term=ECK3888) | 1.643866 |
| [yibN](http://www.ncbi.nlm.nih.gov/entrez/query.fcgi?CMD=search&DB=gene&term=YIBN) | [ECK3601](http://www.ncbi.nlm.nih.gov/entrez/query.fcgi?CMD=search&DB=gene&term=ECK3601) | 2.008352 |
| [ybbB](http://www.ncbi.nlm.nih.gov/entrez/query.fcgi?CMD=search&DB=gene&term=YBBB) | [ECK0496](http://www.ncbi.nlm.nih.gov/entrez/query.fcgi?CMD=search&DB=gene&term=ECK0496) | 2.226119 |
| [ebgC](http://www.ncbi.nlm.nih.gov/entrez/query.fcgi?CMD=search&DB=gene&term=EBGC) | [ECK3067](http://www.ncbi.nlm.nih.gov/entrez/query.fcgi?CMD=search&DB=gene&term=ECK3067) | 2.018050 |
| [dcuA](http://www.ncbi.nlm.nih.gov/entrez/query.fcgi?CMD=search&DB=gene&term=DCUA) | [ECK4132](http://www.ncbi.nlm.nih.gov/entrez/query.fcgi?CMD=search&DB=gene&term=ECK4132) | 1.595225 |
| [B1396](http://www.ncbi.nlm.nih.gov/entrez/query.fcgi?CMD=search&DB=gene&term=B1396) | [ECK1393](http://www.ncbi.nlm.nih.gov/entrez/query.fcgi?CMD=search&DB=gene&term=ECK1393) | 1.551288 |
| [yifM_2](http://www.ncbi.nlm.nih.gov/entrez/query.fcgi?CMD=search&DB=gene&term=YIFM_2) | [ECK3786](http://www.ncbi.nlm.nih.gov/entrez/query.fcgi?CMD=search&DB=gene&term=ECK3786) | 2.259738 |
| [ykgB](http://www.ncbi.nlm.nih.gov/entrez/query.fcgi?CMD=search&DB=gene&term=YKGB) | [ECK0301](http://www.ncbi.nlm.nih.gov/entrez/query.fcgi?CMD=search&DB=gene&term=ECK0301) | 1.883818 |
| [fabG](http://www.ncbi.nlm.nih.gov/entrez/query.fcgi?CMD=search&DB=gene&term=FABG) | [ECK1079](http://www.ncbi.nlm.nih.gov/entrez/query.fcgi?CMD=search&DB=gene&term=ECK1079) | 2.277366 |
| [yajB](http://www.ncbi.nlm.nih.gov/entrez/query.fcgi?CMD=search&DB=gene&term=YAJB) | [ECK0398](http://www.ncbi.nlm.nih.gov/entrez/query.fcgi?CMD=search&DB=gene&term=ECK0398) | 2.009245 |
| [relA](http://www.ncbi.nlm.nih.gov/entrez/query.fcgi?CMD=search&DB=gene&term=RELA) | [ECK2778](http://www.ncbi.nlm.nih.gov/entrez/query.fcgi?CMD=search&DB=gene&term=ECK2778) | 1.631266 |
| [yjhG](http://www.ncbi.nlm.nih.gov/entrez/query.fcgi?CMD=search&DB=gene&term=YJHG) | [ECK4286](http://www.ncbi.nlm.nih.gov/entrez/query.fcgi?CMD=search&DB=gene&term=ECK4286) | 1.545657 |
| [ycfQ](http://www.ncbi.nlm.nih.gov/entrez/query.fcgi?CMD=search&DB=gene&term=YCFQ) | [ECK1097](http://www.ncbi.nlm.nih.gov/entrez/query.fcgi?CMD=search&DB=gene&term=ECK1097) | 1.580843 |
| [nuoF](http://www.ncbi.nlm.nih.gov/entrez/query.fcgi?CMD=search&DB=gene&term=NUOF) | [ECK2278](http://www.ncbi.nlm.nih.gov/entrez/query.fcgi?CMD=search&DB=gene&term=ECK2278) | 1.549868 |
| [xylG](http://www.ncbi.nlm.nih.gov/entrez/query.fcgi?CMD=search&DB=gene&term=XYLG) | [ECK3556](http://www.ncbi.nlm.nih.gov/entrez/query.fcgi?CMD=search&DB=gene&term=ECK3556) | 1.780818 |
| [wcaA](http://www.ncbi.nlm.nih.gov/entrez/query.fcgi?CMD=search&DB=gene&term=WCAA) | [ECK2053](http://www.ncbi.nlm.nih.gov/entrez/query.fcgi?CMD=search&DB=gene&term=ECK2053) | 1.501404 |
| [oppC](http://www.ncbi.nlm.nih.gov/entrez/query.fcgi?CMD=search&DB=gene&term=OPPC) | [ECK1239](http://www.ncbi.nlm.nih.gov/entrez/query.fcgi?CMD=search&DB=gene&term=ECK1239) | 1.605776 |
| [ybdM](http://www.ncbi.nlm.nih.gov/entrez/query.fcgi?CMD=search&DB=gene&term=YBDM) | [ECK0595](http://www.ncbi.nlm.nih.gov/entrez/query.fcgi?CMD=search&DB=gene&term=ECK0595) | 1.531566 |
| [flhB](http://www.ncbi.nlm.nih.gov/entrez/query.fcgi?CMD=search&DB=gene&term=FLHB) | [ECK1881](http://www.ncbi.nlm.nih.gov/entrez/query.fcgi?CMD=search&DB=gene&term=ECK1881) | 2.271594 |
| [phoP](http://www.ncbi.nlm.nih.gov/entrez/query.fcgi?CMD=search&DB=gene&term=PHOP) | [ECK1116](http://www.ncbi.nlm.nih.gov/entrez/query.fcgi?CMD=search&DB=gene&term=ECK1116) | 1.642071 |
| [rplA](http://www.ncbi.nlm.nih.gov/entrez/query.fcgi?CMD=search&DB=gene&term=RPLA) | [ECK3975](http://www.ncbi.nlm.nih.gov/entrez/query.fcgi?CMD=search&DB=gene&term=ECK3975) | 2.221349 |
| [ybbL](http://www.ncbi.nlm.nih.gov/entrez/query.fcgi?CMD=search&DB=gene&term=YBBL) | [ECK0484](http://www.ncbi.nlm.nih.gov/entrez/query.fcgi?CMD=search&DB=gene&term=ECK0484) | 1.664282 |
| [B0499](http://www.ncbi.nlm.nih.gov/entrez/query.fcgi?CMD=search&DB=gene&term=B0499) | [ECK0493](http://www.ncbi.nlm.nih.gov/entrez/query.fcgi?CMD=search&DB=gene&term=ECK0493) | 2.057108 |
| [phoE](http://www.ncbi.nlm.nih.gov/entrez/query.fcgi?CMD=search&DB=gene&term=PHOE) | [ECK0242](http://www.ncbi.nlm.nih.gov/entrez/query.fcgi?CMD=search&DB=gene&term=ECK0242) | 1.864216 |
| [B2789](http://www.ncbi.nlm.nih.gov/entrez/query.fcgi?CMD=search&DB=gene&term=B2789) | [ECK2783](http://www.ncbi.nlm.nih.gov/entrez/query.fcgi?CMD=search&DB=gene&term=ECK2783) | 1.730869 |
| [proA](http://www.ncbi.nlm.nih.gov/entrez/query.fcgi?CMD=search&DB=gene&term=PROA) | [ECK0244](http://www.ncbi.nlm.nih.gov/entrez/query.fcgi?CMD=search&DB=gene&term=ECK0244) | 1.824195 |
| [B2667](http://www.ncbi.nlm.nih.gov/entrez/query.fcgi?CMD=search&DB=gene&term=B2667) | [ECK2661](http://www.ncbi.nlm.nih.gov/entrez/query.fcgi?CMD=search&DB=gene&term=ECK2661) | 1.685932 |
| [mreD](http://www.ncbi.nlm.nih.gov/entrez/query.fcgi?CMD=search&DB=gene&term=MRED) | [ECK3237](http://www.ncbi.nlm.nih.gov/entrez/query.fcgi?CMD=search&DB=gene&term=ECK3237) | 1.961327 |
| [yabP](http://www.ncbi.nlm.nih.gov/entrez/query.fcgi?CMD=search&DB=gene&term=YABP) | [ECK0057](http://www.ncbi.nlm.nih.gov/entrez/query.fcgi?CMD=search&DB=gene&term=ECK0057) | 1.964911 |
| [B0866](http://www.ncbi.nlm.nih.gov/entrez/query.fcgi?CMD=search&DB=gene&term=B0866) | [ECK0857](http://www.ncbi.nlm.nih.gov/entrez/query.fcgi?CMD=search&DB=gene&term=ECK0857) | 1.552536 |
| [yehD](http://www.ncbi.nlm.nih.gov/entrez/query.fcgi?CMD=search&DB=gene&term=YEHD) | [ECK2104](http://www.ncbi.nlm.nih.gov/entrez/query.fcgi?CMD=search&DB=gene&term=ECK2104) | 2.018084 |
| [B1028](http://www.ncbi.nlm.nih.gov/entrez/query.fcgi?CMD=search&DB=gene&term=B1028) | [ECK1016](http://www.ncbi.nlm.nih.gov/entrez/query.fcgi?CMD=search&DB=gene&term=ECK1016) | 1.806833 |
| [aroF](http://www.ncbi.nlm.nih.gov/entrez/query.fcgi?CMD=search&DB=gene&term=AROF) | [ECK2598](http://www.ncbi.nlm.nih.gov/entrez/query.fcgi?CMD=search&DB=gene&term=ECK2598) | 1.566831 |
| [B2603](http://www.ncbi.nlm.nih.gov/entrez/query.fcgi?CMD=search&DB=gene&term=B2603) | [ECK2600](http://www.ncbi.nlm.nih.gov/entrez/query.fcgi?CMD=search&DB=gene&term=ECK2600) | 2.589028 |
| [lpxD](http://www.ncbi.nlm.nih.gov/entrez/query.fcgi?CMD=search&DB=gene&term=LPXD) | [ECK0178](http://www.ncbi.nlm.nih.gov/entrez/query.fcgi?CMD=search&DB=gene&term=ECK0178) | 1.966573 |
| [uhpC](http://www.ncbi.nlm.nih.gov/entrez/query.fcgi?CMD=search&DB=gene&term=UHPC) | [ECK3658](http://www.ncbi.nlm.nih.gov/entrez/query.fcgi?CMD=search&DB=gene&term=ECK3658) | 1.993596 |
| [yddB](http://www.ncbi.nlm.nih.gov/entrez/query.fcgi?CMD=search&DB=gene&term=YDDB) | [ECK1489](http://www.ncbi.nlm.nih.gov/entrez/query.fcgi?CMD=search&DB=gene&term=ECK1489) | 2.420937 |
| [mrdA](http://www.ncbi.nlm.nih.gov/entrez/query.fcgi?CMD=search&DB=gene&term=MRDA) | [ECK0628](http://www.ncbi.nlm.nih.gov/entrez/query.fcgi?CMD=search&DB=gene&term=ECK0628) | 2.032699 |
| [yibP](http://www.ncbi.nlm.nih.gov/entrez/query.fcgi?CMD=search&DB=gene&term=YIBP) | [ECK3603](http://www.ncbi.nlm.nih.gov/entrez/query.fcgi?CMD=search&DB=gene&term=ECK3603) | 1.899523 |
| [secF](http://www.ncbi.nlm.nih.gov/entrez/query.fcgi?CMD=search&DB=gene&term=SECF) | [ECK0403](http://www.ncbi.nlm.nih.gov/entrez/query.fcgi?CMD=search&DB=gene&term=ECK0403) | 1.589414 |
| [yhcC](http://www.ncbi.nlm.nih.gov/entrez/query.fcgi?CMD=search&DB=gene&term=YHCC) | [ECK3201](http://www.ncbi.nlm.nih.gov/entrez/query.fcgi?CMD=search&DB=gene&term=ECK3201) | 1.548309 |
| [ykgE](http://www.ncbi.nlm.nih.gov/entrez/query.fcgi?CMD=search&DB=gene&term=YKGE) | [ECK0305](http://www.ncbi.nlm.nih.gov/entrez/query.fcgi?CMD=search&DB=gene&term=ECK0305) | 1.625563 |
| [ybaX](http://www.ncbi.nlm.nih.gov/entrez/query.fcgi?CMD=search&DB=gene&term=YBAX) | [ECK0438](http://www.ncbi.nlm.nih.gov/entrez/query.fcgi?CMD=search&DB=gene&term=ECK0438) | 2.531246 |
| [ptsG](http://www.ncbi.nlm.nih.gov/entrez/query.fcgi?CMD=search&DB=gene&term=PTSG) | [ECK1087](http://www.ncbi.nlm.nih.gov/entrez/query.fcgi?CMD=search&DB=gene&term=ECK1087) | 1.958491 |
| [ansB](http://www.ncbi.nlm.nih.gov/entrez/query.fcgi?CMD=search&DB=gene&term=ANSB) | [ECK2952](http://www.ncbi.nlm.nih.gov/entrez/query.fcgi?CMD=search&DB=gene&term=ECK2952) | 2.424205 |
| [B2619](http://www.ncbi.nlm.nih.gov/entrez/query.fcgi?CMD=search&DB=gene&term=B2619) | [ECK2615](http://www.ncbi.nlm.nih.gov/entrez/query.fcgi?CMD=search&DB=gene&term=ECK2615) | 1.525717 |
| [yhaV](http://www.ncbi.nlm.nih.gov/entrez/query.fcgi?CMD=search&DB=gene&term=YHAV) | [ECK3118](http://www.ncbi.nlm.nih.gov/entrez/query.fcgi?CMD=search&DB=gene&term=ECK3118) | 2.110994 |
| [ykfD](http://www.ncbi.nlm.nih.gov/entrez/query.fcgi?CMD=search&DB=gene&term=YKFD) | [ECK0262](http://www.ncbi.nlm.nih.gov/entrez/query.fcgi?CMD=search&DB=gene&term=ECK0262) | 1.602560 |
| [rfaS](http://www.ncbi.nlm.nih.gov/entrez/query.fcgi?CMD=search&DB=gene&term=RFAS) | [ECK3619](http://www.ncbi.nlm.nih.gov/entrez/query.fcgi?CMD=search&DB=gene&term=ECK3619) | 1.717686 |
| [yiaC](http://www.ncbi.nlm.nih.gov/entrez/query.fcgi?CMD=search&DB=gene&term=YIAC) | [ECK3537](http://www.ncbi.nlm.nih.gov/entrez/query.fcgi?CMD=search&DB=gene&term=ECK3537) | 2.125623 |
| [dbpA](http://www.ncbi.nlm.nih.gov/entrez/query.fcgi?CMD=search&DB=gene&term=DBPA) | [ECK1340](http://www.ncbi.nlm.nih.gov/entrez/query.fcgi?CMD=search&DB=gene&term=ECK1340) | 1.721711 |
| [yhbZ](http://www.ncbi.nlm.nih.gov/entrez/query.fcgi?CMD=search&DB=gene&term=YHBZ) | [ECK3172](http://www.ncbi.nlm.nih.gov/entrez/query.fcgi?CMD=search&DB=gene&term=ECK3172) | 1.543437 |
| [yraN](http://www.ncbi.nlm.nih.gov/entrez/query.fcgi?CMD=search&DB=gene&term=YRAN) | [ECK3136](http://www.ncbi.nlm.nih.gov/entrez/query.fcgi?CMD=search&DB=gene&term=ECK3136) | 2.050771 |
| [rcsC](http://www.ncbi.nlm.nih.gov/entrez/query.fcgi?CMD=search&DB=gene&term=RCSC) | [ECK2211](http://www.ncbi.nlm.nih.gov/entrez/query.fcgi?CMD=search&DB=gene&term=ECK2211) | 1.669493 |
| [fimD](http://www.ncbi.nlm.nih.gov/entrez/query.fcgi?CMD=search&DB=gene&term=FIMD) | [ECK4308](http://www.ncbi.nlm.nih.gov/entrez/query.fcgi?CMD=search&DB=gene&term=ECK4308) | 1.591184 |
| [agaA](http://www.ncbi.nlm.nih.gov/entrez/query.fcgi?CMD=search&DB=gene&term=AGAA) | [ECK3123](http://www.ncbi.nlm.nih.gov/entrez/query.fcgi?CMD=search&DB=gene&term=ECK3123) | 1.524447 |
| [ygiY](http://www.ncbi.nlm.nih.gov/entrez/query.fcgi?CMD=search&DB=gene&term=YGIY) | [ECK3017](http://www.ncbi.nlm.nih.gov/entrez/query.fcgi?CMD=search&DB=gene&term=ECK3017) | 1.873249 |
| [B2074](http://www.ncbi.nlm.nih.gov/entrez/query.fcgi?CMD=search&DB=gene&term=B2074) | [ECK2070](http://www.ncbi.nlm.nih.gov/entrez/query.fcgi?CMD=search&DB=gene&term=ECK2070) | 2.067866 |
| [cca](http://www.ncbi.nlm.nih.gov/entrez/query.fcgi?CMD=search&DB=gene&term=CCA) | [ECK3046](http://www.ncbi.nlm.nih.gov/entrez/query.fcgi?CMD=search&DB=gene&term=ECK3046) | 1.816671 |
| [greA](http://www.ncbi.nlm.nih.gov/entrez/query.fcgi?CMD=search&DB=gene&term=GREA) | [ECK3170](http://www.ncbi.nlm.nih.gov/entrez/query.fcgi?CMD=search&DB=gene&term=ECK3170) | 2.268726 |
| [B2595](http://www.ncbi.nlm.nih.gov/entrez/query.fcgi?CMD=search&DB=gene&term=B2595) | [ECK2593](http://www.ncbi.nlm.nih.gov/entrez/query.fcgi?CMD=search&DB=gene&term=ECK2593) | 1.891646 |
| [pitA](http://www.ncbi.nlm.nih.gov/entrez/query.fcgi?CMD=search&DB=gene&term=PITA) | [ECK3478](http://www.ncbi.nlm.nih.gov/entrez/query.fcgi?CMD=search&DB=gene&term=ECK3478) | 1.555485 |
| [ycdY](http://www.ncbi.nlm.nih.gov/entrez/query.fcgi?CMD=search&DB=gene&term=YCDY) | [ECK1021](http://www.ncbi.nlm.nih.gov/entrez/query.fcgi?CMD=search&DB=gene&term=ECK1021) | 1.964990 |
| [nirC](http://www.ncbi.nlm.nih.gov/entrez/query.fcgi?CMD=search&DB=gene&term=NIRC) | [ECK3355](http://www.ncbi.nlm.nih.gov/entrez/query.fcgi?CMD=search&DB=gene&term=ECK3355) | 1.693689 |
| [eno](http://www.ncbi.nlm.nih.gov/entrez/query.fcgi?CMD=search&DB=gene&term=ENO) | [ECK2773](http://www.ncbi.nlm.nih.gov/entrez/query.fcgi?CMD=search&DB=gene&term=ECK2773) | 1.686952 |
| [ykfA](http://www.ncbi.nlm.nih.gov/entrez/query.fcgi?CMD=search&DB=gene&term=YKFA) | [ECK0255](http://www.ncbi.nlm.nih.gov/entrez/query.fcgi?CMD=search&DB=gene&term=ECK0255) | 1.607227 |
| [sbmA](http://www.ncbi.nlm.nih.gov/entrez/query.fcgi?CMD=search&DB=gene&term=SBMA) | [ECK0372](http://www.ncbi.nlm.nih.gov/entrez/query.fcgi?CMD=search&DB=gene&term=ECK0372) | 2.316975 |
| [ybaR](http://www.ncbi.nlm.nih.gov/entrez/query.fcgi?CMD=search&DB=gene&term=YBAR) | [ECK0478](http://www.ncbi.nlm.nih.gov/entrez/query.fcgi?CMD=search&DB=gene&term=ECK0478) | 1.719794 |
| [yacK](http://www.ncbi.nlm.nih.gov/entrez/query.fcgi?CMD=search&DB=gene&term=YACK) | [ECK0122](http://www.ncbi.nlm.nih.gov/entrez/query.fcgi?CMD=search&DB=gene&term=ECK0122) | 1.722713 |
| [tehA](http://www.ncbi.nlm.nih.gov/entrez/query.fcgi?CMD=search&DB=gene&term=TEHA) | [ECK1422](http://www.ncbi.nlm.nih.gov/entrez/query.fcgi?CMD=search&DB=gene&term=ECK1422) | 1.577316 |
| [malP](http://www.ncbi.nlm.nih.gov/entrez/query.fcgi?CMD=search&DB=gene&term=MALP) | [ECK3404](http://www.ncbi.nlm.nih.gov/entrez/query.fcgi?CMD=search&DB=gene&term=ECK3404) | 1.512250 |
| [yjbH](http://www.ncbi.nlm.nih.gov/entrez/query.fcgi?CMD=search&DB=gene&term=YJBH) | [ECK4021](http://www.ncbi.nlm.nih.gov/entrez/query.fcgi?CMD=search&DB=gene&term=ECK4021) | 1.813340 |
| [yjeJ](http://www.ncbi.nlm.nih.gov/entrez/query.fcgi?CMD=search&DB=gene&term=YJEJ) | [ECK4139](http://www.ncbi.nlm.nih.gov/entrez/query.fcgi?CMD=search&DB=gene&term=ECK4139) | 1.926837 |
| [yrfD](http://www.ncbi.nlm.nih.gov/entrez/query.fcgi?CMD=search&DB=gene&term=YRFD) | [ECK3382](http://www.ncbi.nlm.nih.gov/entrez/query.fcgi?CMD=search&DB=gene&term=ECK3382) | 1.624641 |
| [ytfE](http://www.ncbi.nlm.nih.gov/entrez/query.fcgi?CMD=search&DB=gene&term=YTFE) | [ECK4205](http://www.ncbi.nlm.nih.gov/entrez/query.fcgi?CMD=search&DB=gene&term=ECK4205) | 1.535802 |
| [tdcE](http://www.ncbi.nlm.nih.gov/entrez/query.fcgi?CMD=search&DB=gene&term=TDCE) | [ECK3103](http://www.ncbi.nlm.nih.gov/entrez/query.fcgi?CMD=search&DB=gene&term=ECK3103) | 1.770911 |
| [B2274](http://www.ncbi.nlm.nih.gov/entrez/query.fcgi?CMD=search&DB=gene&term=B2274) | [ECK2268](http://www.ncbi.nlm.nih.gov/entrez/query.fcgi?CMD=search&DB=gene&term=ECK2268) | 1.560972 |
| [rpiA](http://www.ncbi.nlm.nih.gov/entrez/query.fcgi?CMD=search&DB=gene&term=RPIA) | [ECK2910](http://www.ncbi.nlm.nih.gov/entrez/query.fcgi?CMD=search&DB=gene&term=ECK2910) | 1.521417 |
| [yhjG](http://www.ncbi.nlm.nih.gov/entrez/query.fcgi?CMD=search&DB=gene&term=YHJG) | [ECK3509](http://www.ncbi.nlm.nih.gov/entrez/query.fcgi?CMD=search&DB=gene&term=ECK3509) | 1.549160 |
| [yeaX](http://www.ncbi.nlm.nih.gov/entrez/query.fcgi?CMD=search&DB=gene&term=YEAX) | [ECK1801](http://www.ncbi.nlm.nih.gov/entrez/query.fcgi?CMD=search&DB=gene&term=ECK1801) | 1.758228 |
| [frdA](http://www.ncbi.nlm.nih.gov/entrez/query.fcgi?CMD=search&DB=gene&term=FRDA) | [ECK4150](http://www.ncbi.nlm.nih.gov/entrez/query.fcgi?CMD=search&DB=gene&term=ECK4150) | 1.502872 |
| [ygiN](http://www.ncbi.nlm.nih.gov/entrez/query.fcgi?CMD=search&DB=gene&term=YGIN) | [ECK3020](http://www.ncbi.nlm.nih.gov/entrez/query.fcgi?CMD=search&DB=gene&term=ECK3020) | 2.070768 |
| [fic](http://www.ncbi.nlm.nih.gov/entrez/query.fcgi?CMD=search&DB=gene&term=FIC) | [ECK3349](http://www.ncbi.nlm.nih.gov/entrez/query.fcgi?CMD=search&DB=gene&term=ECK3349) | -1.967541 |
| [B0502](http://www.ncbi.nlm.nih.gov/entrez/query.fcgi?CMD=search&DB=gene&term=B0502) | [ECK0495](http://www.ncbi.nlm.nih.gov/entrez/query.fcgi?CMD=search&DB=gene&term=ECK0495) | -2.271640 |
| [fhuE](http://www.ncbi.nlm.nih.gov/entrez/query.fcgi?CMD=search&DB=gene&term=FHUE) | [ECK1088](http://www.ncbi.nlm.nih.gov/entrez/query.fcgi?CMD=search&DB=gene&term=ECK1088) | -2.936048 |
| [rfaZ](http://www.ncbi.nlm.nih.gov/entrez/query.fcgi?CMD=search&DB=gene&term=RFAZ) | [ECK3614](http://www.ncbi.nlm.nih.gov/entrez/query.fcgi?CMD=search&DB=gene&term=ECK3614) | -1.946559 |
| [nfrB](http://www.ncbi.nlm.nih.gov/entrez/query.fcgi?CMD=search&DB=gene&term=NFRB) | [ECK0561](http://www.ncbi.nlm.nih.gov/entrez/query.fcgi?CMD=search&DB=gene&term=ECK0561) | -1.868078 |
| [yegW](http://www.ncbi.nlm.nih.gov/entrez/query.fcgi?CMD=search&DB=gene&term=YEGW) | [ECK2094](http://www.ncbi.nlm.nih.gov/entrez/query.fcgi?CMD=search&DB=gene&term=ECK2094) | -2.835461 |
| [B1555](http://www.ncbi.nlm.nih.gov/entrez/query.fcgi?CMD=search&DB=gene&term=B1555) | [ECK1549](http://www.ncbi.nlm.nih.gov/entrez/query.fcgi?CMD=search&DB=gene&term=ECK1549) | -1.613058 |
| [B1497](http://www.ncbi.nlm.nih.gov/entrez/query.fcgi?CMD=search&DB=gene&term=B1497) | [ECK1491](http://www.ncbi.nlm.nih.gov/entrez/query.fcgi?CMD=search&DB=gene&term=ECK1491) | -2.528000 |
| [fliA](http://www.ncbi.nlm.nih.gov/entrez/query.fcgi?CMD=search&DB=gene&term=FLIA) | [ECK1921](http://www.ncbi.nlm.nih.gov/entrez/query.fcgi?CMD=search&DB=gene&term=ECK1921) | -4.228811 |
| [ycdU](http://www.ncbi.nlm.nih.gov/entrez/query.fcgi?CMD=search&DB=gene&term=YCDU) | [ECK1017](http://www.ncbi.nlm.nih.gov/entrez/query.fcgi?CMD=search&DB=gene&term=ECK1017) | -1.569491 |
| [ycfA](http://www.ncbi.nlm.nih.gov/entrez/query.fcgi?CMD=search&DB=gene&term=YCFA) | [ECK1142](http://www.ncbi.nlm.nih.gov/entrez/query.fcgi?CMD=search&DB=gene&term=ECK1142) | -2.244534 |
| [fliS](http://www.ncbi.nlm.nih.gov/entrez/query.fcgi?CMD=search&DB=gene&term=FLIS) | [ECK1924](http://www.ncbi.nlm.nih.gov/entrez/query.fcgi?CMD=search&DB=gene&term=ECK1924) | -4.046874 |
| [shiA](http://www.ncbi.nlm.nih.gov/entrez/query.fcgi?CMD=search&DB=gene&term=SHIA) | [ECK1976](http://www.ncbi.nlm.nih.gov/entrez/query.fcgi?CMD=search&DB=gene&term=ECK1976) | -2.279820 |
| [pyrF](http://www.ncbi.nlm.nih.gov/entrez/query.fcgi?CMD=search&DB=gene&term=PYRF) | [ECK1276](http://www.ncbi.nlm.nih.gov/entrez/query.fcgi?CMD=search&DB=gene&term=ECK1276) | -1.868014 |
| [rspA](http://www.ncbi.nlm.nih.gov/entrez/query.fcgi?CMD=search&DB=gene&term=RSPA) | [ECK1576](http://www.ncbi.nlm.nih.gov/entrez/query.fcgi?CMD=search&DB=gene&term=ECK1576) | -1.545076 |
| [ydiT](http://www.ncbi.nlm.nih.gov/entrez/query.fcgi?CMD=search&DB=gene&term=YDIT) | [ECK1698](http://www.ncbi.nlm.nih.gov/entrez/query.fcgi?CMD=search&DB=gene&term=ECK1698) | -2.617584 |
| [asnC](http://www.ncbi.nlm.nih.gov/entrez/query.fcgi?CMD=search&DB=gene&term=ASNC) | [ECK3737](http://www.ncbi.nlm.nih.gov/entrez/query.fcgi?CMD=search&DB=gene&term=ECK3737) | -2.001392 |
| [flhC](http://www.ncbi.nlm.nih.gov/entrez/query.fcgi?CMD=search&DB=gene&term=FLHC) | [ECK1892](http://www.ncbi.nlm.nih.gov/entrez/query.fcgi?CMD=search&DB=gene&term=ECK1892) | -2.561323 |
| [prpC](http://www.ncbi.nlm.nih.gov/entrez/query.fcgi?CMD=search&DB=gene&term=PRPC) | [ECK0330](http://www.ncbi.nlm.nih.gov/entrez/query.fcgi?CMD=search&DB=gene&term=ECK0330) | -2.819996 |
| [B1667](http://www.ncbi.nlm.nih.gov/entrez/query.fcgi?CMD=search&DB=gene&term=B1667) | [ECK1663](http://www.ncbi.nlm.nih.gov/entrez/query.fcgi?CMD=search&DB=gene&term=ECK1663) | -2.163255 |
| [ptxA](http://www.ncbi.nlm.nih.gov/entrez/query.fcgi?CMD=search&DB=gene&term=PTXA) | [ECK4191](http://www.ncbi.nlm.nih.gov/entrez/query.fcgi?CMD=search&DB=gene&term=ECK4191) | -1.504116 |
| [ybfA](http://www.ncbi.nlm.nih.gov/entrez/query.fcgi?CMD=search&DB=gene&term=YBFA) | [ECK0688](http://www.ncbi.nlm.nih.gov/entrez/query.fcgi?CMD=search&DB=gene&term=ECK0688) | -1.576673 |
| [ybcT](http://www.ncbi.nlm.nih.gov/entrez/query.fcgi?CMD=search&DB=gene&term=YBCT) | [ECK0547](http://www.ncbi.nlm.nih.gov/entrez/query.fcgi?CMD=search&DB=gene&term=ECK0547) | -1.600865 |
| [entE](http://www.ncbi.nlm.nih.gov/entrez/query.fcgi?CMD=search&DB=gene&term=ENTE) | [ECK0587](http://www.ncbi.nlm.nih.gov/entrez/query.fcgi?CMD=search&DB=gene&term=ECK0587) | -1.658691 |
| [yccF](http://www.ncbi.nlm.nih.gov/entrez/query.fcgi?CMD=search&DB=gene&term=YCCF) | [ECK0952](http://www.ncbi.nlm.nih.gov/entrez/query.fcgi?CMD=search&DB=gene&term=ECK0952) | -1.526150 |
| [yjfO](http://www.ncbi.nlm.nih.gov/entrez/query.fcgi?CMD=search&DB=gene&term=YJFO) | [ECK4185](http://www.ncbi.nlm.nih.gov/entrez/query.fcgi?CMD=search&DB=gene&term=ECK4185) | -1.507600 |
| [rcsA](http://www.ncbi.nlm.nih.gov/entrez/query.fcgi?CMD=search&DB=gene&term=RCSA) | [ECK1949](http://www.ncbi.nlm.nih.gov/entrez/query.fcgi?CMD=search&DB=gene&term=ECK1949) | -1.628588 |
| [B2670](http://www.ncbi.nlm.nih.gov/entrez/query.fcgi?CMD=search&DB=gene&term=B2670) | [ECK2664](http://www.ncbi.nlm.nih.gov/entrez/query.fcgi?CMD=search&DB=gene&term=ECK2664) | -1.561577 |
| [ydaK](http://www.ncbi.nlm.nih.gov/entrez/query.fcgi?CMD=search&DB=gene&term=YDAK) | [ECK1335](http://www.ncbi.nlm.nih.gov/entrez/query.fcgi?CMD=search&DB=gene&term=ECK1335) | -1.763126 |
| [speE](http://www.ncbi.nlm.nih.gov/entrez/query.fcgi?CMD=search&DB=gene&term=SPEE) | [ECK0120](http://www.ncbi.nlm.nih.gov/entrez/query.fcgi?CMD=search&DB=gene&term=ECK0120) | -3.198427 |
| [yehR](http://www.ncbi.nlm.nih.gov/entrez/query.fcgi?CMD=search&DB=gene&term=YEHR) | [ECK2115](http://www.ncbi.nlm.nih.gov/entrez/query.fcgi?CMD=search&DB=gene&term=ECK2115) | -1.576619 |
| [B1121](http://www.ncbi.nlm.nih.gov/entrez/query.fcgi?CMD=search&DB=gene&term=B1121) | [ECK1107](http://www.ncbi.nlm.nih.gov/entrez/query.fcgi?CMD=search&DB=gene&term=ECK1107) | -1.531827 |
| [aceF](http://www.ncbi.nlm.nih.gov/entrez/query.fcgi?CMD=search&DB=gene&term=ACEF) | [ECK0114](http://www.ncbi.nlm.nih.gov/entrez/query.fcgi?CMD=search&DB=gene&term=ECK0114) | -1.948046 |
| [B2503](http://www.ncbi.nlm.nih.gov/entrez/query.fcgi?CMD=search&DB=gene&term=B2503) | [ECK2499](http://www.ncbi.nlm.nih.gov/entrez/query.fcgi?CMD=search&DB=gene&term=ECK2499) | -1.506300 |
| [B1337](http://www.ncbi.nlm.nih.gov/entrez/query.fcgi?CMD=search&DB=gene&term=B1337) | [ECK1333](http://www.ncbi.nlm.nih.gov/entrez/query.fcgi?CMD=search&DB=gene&term=ECK1333) | -1.998761 |
| [cydA](http://www.ncbi.nlm.nih.gov/entrez/query.fcgi?CMD=search&DB=gene&term=CYDA) | [ECK0721](http://www.ncbi.nlm.nih.gov/entrez/query.fcgi?CMD=search&DB=gene&term=ECK0721) | -1.603705 |
| [yehC](http://www.ncbi.nlm.nih.gov/entrez/query.fcgi?CMD=search&DB=gene&term=YEHC) | [ECK2103](http://www.ncbi.nlm.nih.gov/entrez/query.fcgi?CMD=search&DB=gene&term=ECK2103) | -2.257949 |
| [B1669](http://www.ncbi.nlm.nih.gov/entrez/query.fcgi?CMD=search&DB=gene&term=B1669) | [ECK1665](http://www.ncbi.nlm.nih.gov/entrez/query.fcgi?CMD=search&DB=gene&term=ECK1665) | -1.897453 |
| [ygaC](http://www.ncbi.nlm.nih.gov/entrez/query.fcgi?CMD=search&DB=gene&term=YGAC) | [ECK2665](http://www.ncbi.nlm.nih.gov/entrez/query.fcgi?CMD=search&DB=gene&term=ECK2665) | -1.775239 |
| [sdaA](http://www.ncbi.nlm.nih.gov/entrez/query.fcgi?CMD=search&DB=gene&term=SDAA) | [ECK1812](http://www.ncbi.nlm.nih.gov/entrez/query.fcgi?CMD=search&DB=gene&term=ECK1812) | -1.544358 |
| [yejM](http://www.ncbi.nlm.nih.gov/entrez/query.fcgi?CMD=search&DB=gene&term=YEJM) | [ECK2182](http://www.ncbi.nlm.nih.gov/entrez/query.fcgi?CMD=search&DB=gene&term=ECK2182) | -1.960653 |
| [eutH](http://www.ncbi.nlm.nih.gov/entrez/query.fcgi?CMD=search&DB=gene&term=EUTH) | [ECK2447](http://www.ncbi.nlm.nih.gov/entrez/query.fcgi?CMD=search&DB=gene&term=ECK2447) | -1.949432 |
| [flgG](http://www.ncbi.nlm.nih.gov/entrez/query.fcgi?CMD=search&DB=gene&term=FLGG) | [ECK1063](http://www.ncbi.nlm.nih.gov/entrez/query.fcgi?CMD=search&DB=gene&term=ECK1063) | -6.575605 |
| [ybhC](http://www.ncbi.nlm.nih.gov/entrez/query.fcgi?CMD=search&DB=gene&term=YBHC) | [ECK0761](http://www.ncbi.nlm.nih.gov/entrez/query.fcgi?CMD=search&DB=gene&term=ECK0761) | -1.506937 |
| [glnG](http://www.ncbi.nlm.nih.gov/entrez/query.fcgi?CMD=search&DB=gene&term=GLNG) | [ECK3861](http://www.ncbi.nlm.nih.gov/entrez/query.fcgi?CMD=search&DB=gene&term=ECK3861) | -1.603989 |
| [wzzE](http://www.ncbi.nlm.nih.gov/entrez/query.fcgi?CMD=search&DB=gene&term=WZZE) | [ECK3777](http://www.ncbi.nlm.nih.gov/entrez/query.fcgi?CMD=search&DB=gene&term=ECK3777) | -2.032466 |
| [rnpA](http://www.ncbi.nlm.nih.gov/entrez/query.fcgi?CMD=search&DB=gene&term=RNPA) | [ECK3696](http://www.ncbi.nlm.nih.gov/entrez/query.fcgi?CMD=search&DB=gene&term=ECK3696) | -1.758804 |
| [adiA](http://www.ncbi.nlm.nih.gov/entrez/query.fcgi?CMD=search&DB=gene&term=ADIA) | [ECK4110](http://www.ncbi.nlm.nih.gov/entrez/query.fcgi?CMD=search&DB=gene&term=ECK4110) | -1.592181 |
| [phoR](http://www.ncbi.nlm.nih.gov/entrez/query.fcgi?CMD=search&DB=gene&term=PHOR) | [ECK0394](http://www.ncbi.nlm.nih.gov/entrez/query.fcgi?CMD=search&DB=gene&term=ECK0394) | -2.294967 |
| [ycbQ](http://www.ncbi.nlm.nih.gov/entrez/query.fcgi?CMD=search&DB=gene&term=YCBQ) | [ECK0929](http://www.ncbi.nlm.nih.gov/entrez/query.fcgi?CMD=search&DB=gene&term=ECK0929) | -5.468365 |
| [cpsB](http://www.ncbi.nlm.nih.gov/entrez/query.fcgi?CMD=search&DB=gene&term=CPSB) | [ECK2043](http://www.ncbi.nlm.nih.gov/entrez/query.fcgi?CMD=search&DB=gene&term=ECK2043) | -2.520982 |
| yjbG | [ECK4020](http://www.ncbi.nlm.nih.gov/entrez/query.fcgi?CMD=search&DB=gene&term=ECK4020) | -1.742174 |
| [yciF](http://www.ncbi.nlm.nih.gov/entrez/query.fcgi?CMD=search&DB=gene&term=YCIF) | [ECK1252](http://www.ncbi.nlm.nih.gov/entrez/query.fcgi?CMD=search&DB=gene&term=ECK1252) | -1.786016 |
| [yiiT](http://www.ncbi.nlm.nih.gov/entrez/query.fcgi?CMD=search&DB=gene&term=YIIT) | [ECK3915](http://www.ncbi.nlm.nih.gov/entrez/query.fcgi?CMD=search&DB=gene&term=ECK3915) | -1.596005 |
| [yiaV](http://www.ncbi.nlm.nih.gov/entrez/query.fcgi?CMD=search&DB=gene&term=YIAV) | [ECK3575](http://www.ncbi.nlm.nih.gov/entrez/query.fcgi?CMD=search&DB=gene&term=ECK3575) | -2.244650 |
| [yqeA](http://www.ncbi.nlm.nih.gov/entrez/query.fcgi?CMD=search&DB=gene&term=YQEA) | [ECK2870](http://www.ncbi.nlm.nih.gov/entrez/query.fcgi?CMD=search&DB=gene&term=ECK2870) | -1.697314 |
| [ycjF](http://www.ncbi.nlm.nih.gov/entrez/query.fcgi?CMD=search&DB=gene&term=YCJF) | [ECK1318](http://www.ncbi.nlm.nih.gov/entrez/query.fcgi?CMD=search&DB=gene&term=ECK1318) | -1.817569 |
| [purF](http://www.ncbi.nlm.nih.gov/entrez/query.fcgi?CMD=search&DB=gene&term=PURF) | [ECK2306](http://www.ncbi.nlm.nih.gov/entrez/query.fcgi?CMD=search&DB=gene&term=ECK2306) | -2.055200 |
| [B1481](http://www.ncbi.nlm.nih.gov/entrez/query.fcgi?CMD=search&DB=gene&term=B1481) | [ECK1475](http://www.ncbi.nlm.nih.gov/entrez/query.fcgi?CMD=search&DB=gene&term=ECK1475) | -1.567744 |
| [yhaP](http://www.ncbi.nlm.nih.gov/entrez/query.fcgi?CMD=search&DB=gene&term=YHAP) | [ECK3101](http://www.ncbi.nlm.nih.gov/entrez/query.fcgi?CMD=search&DB=gene&term=ECK3101) | -2.177024 |
| [yjjB](http://www.ncbi.nlm.nih.gov/entrez/query.fcgi?CMD=search&DB=gene&term=YJJB) | [ECK4353](http://www.ncbi.nlm.nih.gov/entrez/query.fcgi?CMD=search&DB=gene&term=ECK4353) | -1.716848 |
| [sanA](http://www.ncbi.nlm.nih.gov/entrez/query.fcgi?CMD=search&DB=gene&term=SANA) | [ECK2137](http://www.ncbi.nlm.nih.gov/entrez/query.fcgi?CMD=search&DB=gene&term=ECK2137) | -2.409350 |
| [intF](http://www.ncbi.nlm.nih.gov/entrez/query.fcgi?CMD=search&DB=gene&term=INTF) | [ECK0280](http://www.ncbi.nlm.nih.gov/entrez/query.fcgi?CMD=search&DB=gene&term=ECK0280) | -2.459768 |
| [B3472](http://www.ncbi.nlm.nih.gov/entrez/query.fcgi?CMD=search&DB=gene&term=B3472) | [ECK3456](http://www.ncbi.nlm.nih.gov/entrez/query.fcgi?CMD=search&DB=gene&term=ECK3456) | -2.239567 |
| [ybhI](http://www.ncbi.nlm.nih.gov/entrez/query.fcgi?CMD=search&DB=gene&term=YBHI) | [ECK0759](http://www.ncbi.nlm.nih.gov/entrez/query.fcgi?CMD=search&DB=gene&term=ECK0759) | -2.990642 |
| [umuC](http://www.ncbi.nlm.nih.gov/entrez/query.fcgi?CMD=search&DB=gene&term=UMUC) | [ECK1172](http://www.ncbi.nlm.nih.gov/entrez/query.fcgi?CMD=search&DB=gene&term=ECK1172) | -1.983907 |
| [yhjM](http://www.ncbi.nlm.nih.gov/entrez/query.fcgi?CMD=search&DB=gene&term=YHJM) | [ECK3516](http://www.ncbi.nlm.nih.gov/entrez/query.fcgi?CMD=search&DB=gene&term=ECK3516) | -1.739291 |
| [ycdQ](http://www.ncbi.nlm.nih.gov/entrez/query.fcgi?CMD=search&DB=gene&term=YCDQ) | [ECK1012](http://www.ncbi.nlm.nih.gov/entrez/query.fcgi?CMD=search&DB=gene&term=ECK1012) | -1.634310 |
| [B1978](http://www.ncbi.nlm.nih.gov/entrez/query.fcgi?CMD=search&DB=gene&term=B1978) | [ECK1974](http://www.ncbi.nlm.nih.gov/entrez/query.fcgi?CMD=search&DB=gene&term=ECK1974) | -1.629617 |
| [ygjO](http://www.ncbi.nlm.nih.gov/entrez/query.fcgi?CMD=search&DB=gene&term=YGJO) | [ECK3074](http://www.ncbi.nlm.nih.gov/entrez/query.fcgi?CMD=search&DB=gene&term=ECK3074) | -4.065674 |
| [frdC](http://www.ncbi.nlm.nih.gov/entrez/query.fcgi?CMD=search&DB=gene&term=FRDC) | [ECK4148](http://www.ncbi.nlm.nih.gov/entrez/query.fcgi?CMD=search&DB=gene&term=ECK4148) | -2.444496 |
| [ybaT](http://www.ncbi.nlm.nih.gov/entrez/query.fcgi?CMD=search&DB=gene&term=YBAT) | [ECK0480](http://www.ncbi.nlm.nih.gov/entrez/query.fcgi?CMD=search&DB=gene&term=ECK0480) | -3.343506 |
| [baeS](http://www.ncbi.nlm.nih.gov/entrez/query.fcgi?CMD=search&DB=gene&term=BAES) | [ECK2074](http://www.ncbi.nlm.nih.gov/entrez/query.fcgi?CMD=search&DB=gene&term=ECK2074) | -1.538282 |
| [yggF](http://www.ncbi.nlm.nih.gov/entrez/query.fcgi?CMD=search&DB=gene&term=YGGF) | [ECK2926](http://www.ncbi.nlm.nih.gov/entrez/query.fcgi?CMD=search&DB=gene&term=ECK2926) | -1.614458 |
| [yqcE](http://www.ncbi.nlm.nih.gov/entrez/query.fcgi?CMD=search&DB=gene&term=YQCE) | [ECK2769](http://www.ncbi.nlm.nih.gov/entrez/query.fcgi?CMD=search&DB=gene&term=ECK2769) | -1.628739 |
| [sfmA](http://www.ncbi.nlm.nih.gov/entrez/query.fcgi?CMD=search&DB=gene&term=SFMA) | [ECK0523](http://www.ncbi.nlm.nih.gov/entrez/query.fcgi?CMD=search&DB=gene&term=ECK0523) | -2.012430 |
| [ynaJ](http://www.ncbi.nlm.nih.gov/entrez/query.fcgi?CMD=search&DB=gene&term=YNAJ) | [ECK1328](http://www.ncbi.nlm.nih.gov/entrez/query.fcgi?CMD=search&DB=gene&term=ECK1328) | -1.593639 |
| [ycgY](http://www.ncbi.nlm.nih.gov/entrez/query.fcgi?CMD=search&DB=gene&term=YCGY) | [ECK1184](http://www.ncbi.nlm.nih.gov/entrez/query.fcgi?CMD=search&DB=gene&term=ECK1184) | -2.300155 |
| [B2817](http://www.ncbi.nlm.nih.gov/entrez/query.fcgi?CMD=search&DB=gene&term=B2817) | [ECK2813](http://www.ncbi.nlm.nih.gov/entrez/query.fcgi?CMD=search&DB=gene&term=ECK2813) | -1.521270 |
| [sucA](http://www.ncbi.nlm.nih.gov/entrez/query.fcgi?CMD=search&DB=gene&term=SUCA) | [ECK0714](http://www.ncbi.nlm.nih.gov/entrez/query.fcgi?CMD=search&DB=gene&term=ECK0714) | -1.761891 |
| [B1192](http://www.ncbi.nlm.nih.gov/entrez/query.fcgi?CMD=search&DB=gene&term=B1192) | [ECK1180](http://www.ncbi.nlm.nih.gov/entrez/query.fcgi?CMD=search&DB=gene&term=ECK1180) | -1.555839 |
| [fur](http://www.ncbi.nlm.nih.gov/entrez/query.fcgi?CMD=search&DB=gene&term=FUR) | [ECK0671](http://www.ncbi.nlm.nih.gov/entrez/query.fcgi?CMD=search&DB=gene&term=ECK0671) | -1.563568 |
| [yhcD](http://www.ncbi.nlm.nih.gov/entrez/query.fcgi?CMD=search&DB=gene&term=YHCD) | [ECK3206](http://www.ncbi.nlm.nih.gov/entrez/query.fcgi?CMD=search&DB=gene&term=ECK3206) | -1.604454 |
| [yfgA](http://www.ncbi.nlm.nih.gov/entrez/query.fcgi?CMD=search&DB=gene&term=YFGA) | [ECK2512](http://www.ncbi.nlm.nih.gov/entrez/query.fcgi?CMD=search&DB=gene&term=ECK2512) | -3.952218 |
| [ybbV](http://www.ncbi.nlm.nih.gov/entrez/query.fcgi?CMD=search&DB=gene&term=YBBV) | [ECK0503](http://www.ncbi.nlm.nih.gov/entrez/query.fcgi?CMD=search&DB=gene&term=ECK0503) | -1.556440 |
| [yiiM](http://www.ncbi.nlm.nih.gov/entrez/query.fcgi?CMD=search&DB=gene&term=YIIM) | [ECK3903](http://www.ncbi.nlm.nih.gov/entrez/query.fcgi?CMD=search&DB=gene&term=ECK3903) | -2.210097 |
| [B1586](http://www.ncbi.nlm.nih.gov/entrez/query.fcgi?CMD=search&DB=gene&term=B1586) | [ECK1581](http://www.ncbi.nlm.nih.gov/entrez/query.fcgi?CMD=search&DB=gene&term=ECK1581) | -1.552847 |
| [fabH](http://www.ncbi.nlm.nih.gov/entrez/query.fcgi?CMD=search&DB=gene&term=FABH) | [ECK1077](http://www.ncbi.nlm.nih.gov/entrez/query.fcgi?CMD=search&DB=gene&term=ECK1077) | -1.718481 |
| [yifM_1](http://www.ncbi.nlm.nih.gov/entrez/query.fcgi?CMD=search&DB=gene&term=YIFM_1) | [ECK3786](http://www.ncbi.nlm.nih.gov/entrez/query.fcgi?CMD=search&DB=gene&term=ECK3786) | -1.719893 |
| [B1489](http://www.ncbi.nlm.nih.gov/entrez/query.fcgi?CMD=search&DB=gene&term=B1489) | [ECK1483](http://www.ncbi.nlm.nih.gov/entrez/query.fcgi?CMD=search&DB=gene&term=ECK1483) | -1.603816 |
| [lacY](http://www.ncbi.nlm.nih.gov/entrez/query.fcgi?CMD=search&DB=gene&term=LACY) | [ECK0340](http://www.ncbi.nlm.nih.gov/entrez/query.fcgi?CMD=search&DB=gene&term=ECK0340) | -1.942322 |
| [carA](http://www.ncbi.nlm.nih.gov/entrez/query.fcgi?CMD=search&DB=gene&term=CARA) | [ECK0033](http://www.ncbi.nlm.nih.gov/entrez/query.fcgi?CMD=search&DB=gene&term=ECK0033) | -1.589625 |
| [tsx](http://www.ncbi.nlm.nih.gov/entrez/query.fcgi?CMD=search&DB=gene&term=TSX) | [ECK0405](http://www.ncbi.nlm.nih.gov/entrez/query.fcgi?CMD=search&DB=gene&term=ECK0405) | -1.995846 |
| [dsbD](http://www.ncbi.nlm.nih.gov/entrez/query.fcgi?CMD=search&DB=gene&term=DSBD) | [ECK4130](http://www.ncbi.nlm.nih.gov/entrez/query.fcgi?CMD=search&DB=gene&term=ECK4130) | -1.932282 |
| [himD](http://www.ncbi.nlm.nih.gov/entrez/query.fcgi?CMD=search&DB=gene&term=HIMD) | [ECK0903](http://www.ncbi.nlm.nih.gov/entrez/query.fcgi?CMD=search&DB=gene&term=ECK0903) | -1.896070 |
| [B1598](http://www.ncbi.nlm.nih.gov/entrez/query.fcgi?CMD=search&DB=gene&term=B1598) | [ECK1593](http://www.ncbi.nlm.nih.gov/entrez/query.fcgi?CMD=search&DB=gene&term=ECK1593) | -3.255304 |
| [ilvN](http://www.ncbi.nlm.nih.gov/entrez/query.fcgi?CMD=search&DB=gene&term=ILVN) | [ECK3661](http://www.ncbi.nlm.nih.gov/entrez/query.fcgi?CMD=search&DB=gene&term=ECK3661) | -2.336420 |
| [B1007](http://www.ncbi.nlm.nih.gov/entrez/query.fcgi?CMD=search&DB=gene&term=B1007) | [ECK0998](http://www.ncbi.nlm.nih.gov/entrez/query.fcgi?CMD=search&DB=gene&term=ECK0998) | -2.026708 |
| [narH](http://www.ncbi.nlm.nih.gov/entrez/query.fcgi?CMD=search&DB=gene&term=NARH) | [ECK1219](http://www.ncbi.nlm.nih.gov/entrez/query.fcgi?CMD=search&DB=gene&term=ECK1219) | -2.279035 |
| [yraK](http://www.ncbi.nlm.nih.gov/entrez/query.fcgi?CMD=search&DB=gene&term=YRAK) | [ECK3133](http://www.ncbi.nlm.nih.gov/entrez/query.fcgi?CMD=search&DB=gene&term=ECK3133) | -1.558122 |
| [gcvT](http://www.ncbi.nlm.nih.gov/entrez/query.fcgi?CMD=search&DB=gene&term=GCVT) | [ECK2900](http://www.ncbi.nlm.nih.gov/entrez/query.fcgi?CMD=search&DB=gene&term=ECK2900) | -1.701913 |
| [osmC](http://www.ncbi.nlm.nih.gov/entrez/query.fcgi?CMD=search&DB=gene&term=OSMC) | [ECK1476](http://www.ncbi.nlm.nih.gov/entrez/query.fcgi?CMD=search&DB=gene&term=ECK1476) | -2.169895 |
| [B0830](http://www.ncbi.nlm.nih.gov/entrez/query.fcgi?CMD=search&DB=gene&term=B0830) | [ECK0820](http://www.ncbi.nlm.nih.gov/entrez/query.fcgi?CMD=search&DB=gene&term=ECK0820) | -1.805138 |
| [ycgN](http://www.ncbi.nlm.nih.gov/entrez/query.fcgi?CMD=search&DB=gene&term=YCGN) | [ECK1169](http://www.ncbi.nlm.nih.gov/entrez/query.fcgi?CMD=search&DB=gene&term=ECK1169) | -1.547768 |
| [ilvY](http://www.ncbi.nlm.nih.gov/entrez/query.fcgi?CMD=search&DB=gene&term=ILVY) | [ECK3765](http://www.ncbi.nlm.nih.gov/entrez/query.fcgi?CMD=search&DB=gene&term=ECK3765) | -2.141972 |
| [htgA](http://www.ncbi.nlm.nih.gov/entrez/query.fcgi?CMD=search&DB=gene&term=HTGA) | [ECK0012](http://www.ncbi.nlm.nih.gov/entrez/query.fcgi?CMD=search&DB=gene&term=ECK0012) | -1.764229 |
| [fecA](http://www.ncbi.nlm.nih.gov/entrez/query.fcgi?CMD=search&DB=gene&term=FECA) | [ECK4281](http://www.ncbi.nlm.nih.gov/entrez/query.fcgi?CMD=search&DB=gene&term=ECK4281) | -1.719398 |
| [B2878](http://www.ncbi.nlm.nih.gov/entrez/query.fcgi?CMD=search&DB=gene&term=B2878) | [ECK2874](http://www.ncbi.nlm.nih.gov/entrez/query.fcgi?CMD=search&DB=gene&term=ECK2874) | -1.888837 |
| [rpsJ](http://www.ncbi.nlm.nih.gov/entrez/query.fcgi?CMD=search&DB=gene&term=RPSJ) | [ECK3308](http://www.ncbi.nlm.nih.gov/entrez/query.fcgi?CMD=search&DB=gene&term=ECK3308) | -1.864991 |
| [glnL](http://www.ncbi.nlm.nih.gov/entrez/query.fcgi?CMD=search&DB=gene&term=GLNL) | [ECK3862](http://www.ncbi.nlm.nih.gov/entrez/query.fcgi?CMD=search&DB=gene&term=ECK3862) | -1.567770 |
| [yjeE](http://www.ncbi.nlm.nih.gov/entrez/query.fcgi?CMD=search&DB=gene&term=YJEE) | [ECK4164](http://www.ncbi.nlm.nih.gov/entrez/query.fcgi?CMD=search&DB=gene&term=ECK4164) | -2.692204 |
| [rimJ](http://www.ncbi.nlm.nih.gov/entrez/query.fcgi?CMD=search&DB=gene&term=RIMJ) | [ECK1051](http://www.ncbi.nlm.nih.gov/entrez/query.fcgi?CMD=search&DB=gene&term=ECK1051) | -2.538217 |
| [yheE](http://www.ncbi.nlm.nih.gov/entrez/query.fcgi?CMD=search&DB=gene&term=YHEE) | [ECK3311](http://www.ncbi.nlm.nih.gov/entrez/query.fcgi?CMD=search&DB=gene&term=ECK3311) | -1.582575 |
| [wcaJ](http://www.ncbi.nlm.nih.gov/entrez/query.fcgi?CMD=search&DB=gene&term=WCAJ) | [ECK2041](http://www.ncbi.nlm.nih.gov/entrez/query.fcgi?CMD=search&DB=gene&term=ECK2041) | -1.738650 |
| [frvB](http://www.ncbi.nlm.nih.gov/entrez/query.fcgi?CMD=search&DB=gene&term=FRVB) | [ECK3892](http://www.ncbi.nlm.nih.gov/entrez/query.fcgi?CMD=search&DB=gene&term=ECK3892) | -2.216503 |
| [B1330](http://www.ncbi.nlm.nih.gov/entrez/query.fcgi?CMD=search&DB=gene&term=B1330) | [ECK1327](http://www.ncbi.nlm.nih.gov/entrez/query.fcgi?CMD=search&DB=gene&term=ECK1327) | -1.696372 |
| [yheG](http://www.ncbi.nlm.nih.gov/entrez/query.fcgi?CMD=search&DB=gene&term=YHEG) | [ECK3313](http://www.ncbi.nlm.nih.gov/entrez/query.fcgi?CMD=search&DB=gene&term=ECK3313) | -1.637964 |
| [aldA](http://www.ncbi.nlm.nih.gov/entrez/query.fcgi?CMD=search&DB=gene&term=ALDA) | [ECK1408](http://www.ncbi.nlm.nih.gov/entrez/query.fcgi?CMD=search&DB=gene&term=ECK1408) | -1.838882 |
| [yjfM](http://www.ncbi.nlm.nih.gov/entrez/query.fcgi?CMD=search&DB=gene&term=YJFM) | [ECK4181](http://www.ncbi.nlm.nih.gov/entrez/query.fcgi?CMD=search&DB=gene&term=ECK4181) | -3.688797 |
| [amtB](http://www.ncbi.nlm.nih.gov/entrez/query.fcgi?CMD=search&DB=gene&term=AMTB) | [ECK0445](http://www.ncbi.nlm.nih.gov/entrez/query.fcgi?CMD=search&DB=gene&term=ECK0445) | -1.753768 |
| [B1815](http://www.ncbi.nlm.nih.gov/entrez/query.fcgi?CMD=search&DB=gene&term=B1815) | [ECK1813](http://www.ncbi.nlm.nih.gov/entrez/query.fcgi?CMD=search&DB=gene&term=ECK1813) | -1.509535 |
| [hyaC](http://www.ncbi.nlm.nih.gov/entrez/query.fcgi?CMD=search&DB=gene&term=HYAC) | [ECK0965](http://www.ncbi.nlm.nih.gov/entrez/query.fcgi?CMD=search&DB=gene&term=ECK0965) | -1.979108 |
| [yagS](http://www.ncbi.nlm.nih.gov/entrez/query.fcgi?CMD=search&DB=gene&term=YAGS) | [ECK0284](http://www.ncbi.nlm.nih.gov/entrez/query.fcgi?CMD=search&DB=gene&term=ECK0284) | -1.692900 |
| [ygfO](http://www.ncbi.nlm.nih.gov/entrez/query.fcgi?CMD=search&DB=gene&term=YGFO) | [ECK2878](http://www.ncbi.nlm.nih.gov/entrez/query.fcgi?CMD=search&DB=gene&term=ECK2878) | -2.367834 |
| [ycbO](http://www.ncbi.nlm.nih.gov/entrez/query.fcgi?CMD=search&DB=gene&term=YCBO) | [ECK0927](http://www.ncbi.nlm.nih.gov/entrez/query.fcgi?CMD=search&DB=gene&term=ECK0927) | -1.512055 |
| [citF](http://www.ncbi.nlm.nih.gov/entrez/query.fcgi?CMD=search&DB=gene&term=CITF) | [ECK0608](http://www.ncbi.nlm.nih.gov/entrez/query.fcgi?CMD=search&DB=gene&term=ECK0608) | -1.737230 |
| [panC](http://www.ncbi.nlm.nih.gov/entrez/query.fcgi?CMD=search&DB=gene&term=PANC) | [ECK0132](http://www.ncbi.nlm.nih.gov/entrez/query.fcgi?CMD=search&DB=gene&term=ECK0132) | -3.065781 |
| [ygbB](http://www.ncbi.nlm.nih.gov/entrez/query.fcgi?CMD=search&DB=gene&term=YGBB) | [ECK2741](http://www.ncbi.nlm.nih.gov/entrez/query.fcgi?CMD=search&DB=gene&term=ECK2741) | -1.720791 |
| [nuoC](http://www.ncbi.nlm.nih.gov/entrez/query.fcgi?CMD=search&DB=gene&term=NUOC) | [ECK2280](http://www.ncbi.nlm.nih.gov/entrez/query.fcgi?CMD=search&DB=gene&term=ECK2280) | -2.081084 |
| [wecD](http://www.ncbi.nlm.nih.gov/entrez/query.fcgi?CMD=search&DB=gene&term=WECD) | [ECK3782](http://www.ncbi.nlm.nih.gov/entrez/query.fcgi?CMD=search&DB=gene&term=ECK3782) | -1.639006 |
| [yhjX](http://www.ncbi.nlm.nih.gov/entrez/query.fcgi?CMD=search&DB=gene&term=YHJX) | [ECK3534](http://www.ncbi.nlm.nih.gov/entrez/query.fcgi?CMD=search&DB=gene&term=ECK3534) | -1.728182 |
| [yhiI](http://www.ncbi.nlm.nih.gov/entrez/query.fcgi?CMD=search&DB=gene&term=YHII) | [ECK3472](http://www.ncbi.nlm.nih.gov/entrez/query.fcgi?CMD=search&DB=gene&term=ECK3472) | -2.403598 |
| [rplU](http://www.ncbi.nlm.nih.gov/entrez/query.fcgi?CMD=search&DB=gene&term=RPLU) | [ECK3175](http://www.ncbi.nlm.nih.gov/entrez/query.fcgi?CMD=search&DB=gene&term=ECK3175) | -2.177419 |
| [glnB](http://www.ncbi.nlm.nih.gov/entrez/query.fcgi?CMD=search&DB=gene&term=GLNB) | [ECK2550](http://www.ncbi.nlm.nih.gov/entrez/query.fcgi?CMD=search&DB=gene&term=ECK2550) | -1.977844 |
| [mdh-R](http://www.ncbi.nlm.nih.gov/entrez/query.fcgi?CMD=search&DB=gene&term=MDH-R) | [ECK3225](http://www.ncbi.nlm.nih.gov/entrez/query.fcgi?CMD=search&DB=gene&term=ECK3225) | -2.373228 |
| [tnaA](http://www.ncbi.nlm.nih.gov/entrez/query.fcgi?CMD=search&DB=gene&term=TNAA) | [ECK3701](http://www.ncbi.nlm.nih.gov/entrez/query.fcgi?CMD=search&DB=gene&term=ECK3701) | -4.752225 |
| [ygfJ](http://www.ncbi.nlm.nih.gov/entrez/query.fcgi?CMD=search&DB=gene&term=YGFJ) | [ECK2873](http://www.ncbi.nlm.nih.gov/entrez/query.fcgi?CMD=search&DB=gene&term=ECK2873) | -2.367405 |
| [B2641](http://www.ncbi.nlm.nih.gov/entrez/query.fcgi?CMD=search&DB=gene&term=B2641) | [ECK2637](http://www.ncbi.nlm.nih.gov/entrez/query.fcgi?CMD=search&DB=gene&term=ECK2637) | -1.710454 |
| [pdxK](http://www.ncbi.nlm.nih.gov/entrez/query.fcgi?CMD=search&DB=gene&term=PDXK) | [ECK2413](http://www.ncbi.nlm.nih.gov/entrez/query.fcgi?CMD=search&DB=gene&term=ECK2413) | -1.814420 |
| [ybgA](http://www.ncbi.nlm.nih.gov/entrez/query.fcgi?CMD=search&DB=gene&term=YBGA) | [ECK0696](http://www.ncbi.nlm.nih.gov/entrez/query.fcgi?CMD=search&DB=gene&term=ECK0696) | -3.385054 |
| [B2080](http://www.ncbi.nlm.nih.gov/entrez/query.fcgi?CMD=search&DB=gene&term=B2080) | [ECK2076](http://www.ncbi.nlm.nih.gov/entrez/query.fcgi?CMD=search&DB=gene&term=ECK2076) | -2.131119 |
| [B1827](http://www.ncbi.nlm.nih.gov/entrez/query.fcgi?CMD=search&DB=gene&term=B1827) | [ECK1826](http://www.ncbi.nlm.nih.gov/entrez/query.fcgi?CMD=search&DB=gene&term=ECK1826) | -2.561012 |
| [ydbC](http://www.ncbi.nlm.nih.gov/entrez/query.fcgi?CMD=search&DB=gene&term=YDBC) | [ECK1399](http://www.ncbi.nlm.nih.gov/entrez/query.fcgi?CMD=search&DB=gene&term=ECK1399) | -2.074521 |
| [leuB](http://www.ncbi.nlm.nih.gov/entrez/query.fcgi?CMD=search&DB=gene&term=LEUB) | [ECK0075](http://www.ncbi.nlm.nih.gov/entrez/query.fcgi?CMD=search&DB=gene&term=ECK0075) | -1.513399 |
| [bisC](http://www.ncbi.nlm.nih.gov/entrez/query.fcgi?CMD=search&DB=gene&term=BISC) | [ECK3538](http://www.ncbi.nlm.nih.gov/entrez/query.fcgi?CMD=search&DB=gene&term=ECK3538) | -1.831177 |
| [ybgR](http://www.ncbi.nlm.nih.gov/entrez/query.fcgi?CMD=search&DB=gene&term=YBGR) | [ECK0741](http://www.ncbi.nlm.nih.gov/entrez/query.fcgi?CMD=search&DB=gene&term=ECK0741) | -1.806756 |
| [prmA](http://www.ncbi.nlm.nih.gov/entrez/query.fcgi?CMD=search&DB=gene&term=PRMA) | [ECK3246](http://www.ncbi.nlm.nih.gov/entrez/query.fcgi?CMD=search&DB=gene&term=ECK3246) | -2.241461 |
| [sms](http://www.ncbi.nlm.nih.gov/entrez/query.fcgi?CMD=search&DB=gene&term=SMS) | [ECK4381](http://www.ncbi.nlm.nih.gov/entrez/query.fcgi?CMD=search&DB=gene&term=ECK4381) | -1.618143 |
| [lipA](http://www.ncbi.nlm.nih.gov/entrez/query.fcgi?CMD=search&DB=gene&term=LIPA) | [ECK0621](http://www.ncbi.nlm.nih.gov/entrez/query.fcgi?CMD=search&DB=gene&term=ECK0621) | -3.236206 |
| [yaaF](http://www.ncbi.nlm.nih.gov/entrez/query.fcgi?CMD=search&DB=gene&term=YAAF) | [ECK0031](http://www.ncbi.nlm.nih.gov/entrez/query.fcgi?CMD=search&DB=gene&term=ECK0031) | -1.519973 |
| [wecE](http://www.ncbi.nlm.nih.gov/entrez/query.fcgi?CMD=search&DB=gene&term=WECE) | [ECK3783](http://www.ncbi.nlm.nih.gov/entrez/query.fcgi?CMD=search&DB=gene&term=ECK3783) | -2.102368 |
| [oppA](http://www.ncbi.nlm.nih.gov/entrez/query.fcgi?CMD=search&DB=gene&term=OPPA) | [ECK1237](http://www.ncbi.nlm.nih.gov/entrez/query.fcgi?CMD=search&DB=gene&term=ECK1237) | -1.635115 |
| [dsrB](http://www.ncbi.nlm.nih.gov/entrez/query.fcgi?CMD=search&DB=gene&term=DSRB) | [ECK1950](http://www.ncbi.nlm.nih.gov/entrez/query.fcgi?CMD=search&DB=gene&term=ECK1950) | -2.425996 |
| [yagY](http://www.ncbi.nlm.nih.gov/entrez/query.fcgi?CMD=search&DB=gene&term=YAGY) | [ECK0291](http://www.ncbi.nlm.nih.gov/entrez/query.fcgi?CMD=search&DB=gene&term=ECK0291) | -2.453305 |
| [yeeX](http://www.ncbi.nlm.nih.gov/entrez/query.fcgi?CMD=search&DB=gene&term=YEEX) | [ECK2001](http://www.ncbi.nlm.nih.gov/entrez/query.fcgi?CMD=search&DB=gene&term=ECK2001) | -1.997556 |
| [ybeU](http://www.ncbi.nlm.nih.gov/entrez/query.fcgi?CMD=search&DB=gene&term=YBEU) | [ECK0641](http://www.ncbi.nlm.nih.gov/entrez/query.fcgi?CMD=search&DB=gene&term=ECK0641) | -2.791618 |
| [yfiA](http://www.ncbi.nlm.nih.gov/entrez/query.fcgi?CMD=search&DB=gene&term=YFIA) | [ECK2594](http://www.ncbi.nlm.nih.gov/entrez/query.fcgi?CMD=search&DB=gene&term=ECK2594) | -1.508843 |
| [yccE](http://www.ncbi.nlm.nih.gov/entrez/query.fcgi?CMD=search&DB=gene&term=YCCE) | [ECK0992](http://www.ncbi.nlm.nih.gov/entrez/query.fcgi?CMD=search&DB=gene&term=ECK0992) | -2.023104 |
| [ycfL](http://www.ncbi.nlm.nih.gov/entrez/query.fcgi?CMD=search&DB=gene&term=YCFL) | [ECK1090](http://www.ncbi.nlm.nih.gov/entrez/query.fcgi?CMD=search&DB=gene&term=ECK1090) | -3.039359 |
| [yhhG](http://www.ncbi.nlm.nih.gov/entrez/query.fcgi?CMD=search&DB=gene&term=YHHG) | [ECK3465](http://www.ncbi.nlm.nih.gov/entrez/query.fcgi?CMD=search&DB=gene&term=ECK3465) | -1.560841 |
| [fabA](http://www.ncbi.nlm.nih.gov/entrez/query.fcgi?CMD=search&DB=gene&term=FABA) | [ECK0945](http://www.ncbi.nlm.nih.gov/entrez/query.fcgi?CMD=search&DB=gene&term=ECK0945) | -1.508455 |
| [yhaJ](http://www.ncbi.nlm.nih.gov/entrez/query.fcgi?CMD=search&DB=gene&term=YHAJ) | [ECK3096](http://www.ncbi.nlm.nih.gov/entrez/query.fcgi?CMD=search&DB=gene&term=ECK3096) | -2.080990 |
| [dnaN](http://www.ncbi.nlm.nih.gov/entrez/query.fcgi?CMD=search&DB=gene&term=DNAN) | [ECK3693](http://www.ncbi.nlm.nih.gov/entrez/query.fcgi?CMD=search&DB=gene&term=ECK3693) | -2.375741 |
| [agaY](http://www.ncbi.nlm.nih.gov/entrez/query.fcgi?CMD=search&DB=gene&term=AGAY) | [ECK3125](http://www.ncbi.nlm.nih.gov/entrez/query.fcgi?CMD=search&DB=gene&term=ECK3125) | -1.515141 |
| [B2071](http://www.ncbi.nlm.nih.gov/entrez/query.fcgi?CMD=search&DB=gene&term=B2071) | [ECK2065](http://www.ncbi.nlm.nih.gov/entrez/query.fcgi?CMD=search&DB=gene&term=ECK2065) | -1.531424 |
| [crr](http://www.ncbi.nlm.nih.gov/entrez/query.fcgi?CMD=search&DB=gene&term=CRR) | [ECK2412](http://www.ncbi.nlm.nih.gov/entrez/query.fcgi?CMD=search&DB=gene&term=ECK2412) | -3.337411 |
| [nrdE](http://www.ncbi.nlm.nih.gov/entrez/query.fcgi?CMD=search&DB=gene&term=NRDE) | [ECK2669](http://www.ncbi.nlm.nih.gov/entrez/query.fcgi?CMD=search&DB=gene&term=ECK2669) | -2.537358 |
| [ygfD](http://www.ncbi.nlm.nih.gov/entrez/query.fcgi?CMD=search&DB=gene&term=YGFD) | [ECK2914](http://www.ncbi.nlm.nih.gov/entrez/query.fcgi?CMD=search&DB=gene&term=ECK2914) | -1.695011 |
| [yoaG](http://www.ncbi.nlm.nih.gov/entrez/query.fcgi?CMD=search&DB=gene&term=YOAG) | [ECK1794](http://www.ncbi.nlm.nih.gov/entrez/query.fcgi?CMD=search&DB=gene&term=ECK1794) | -1.957386 |
| [cvpA](http://www.ncbi.nlm.nih.gov/entrez/query.fcgi?CMD=search&DB=gene&term=CVPA) | [ECK2307](http://www.ncbi.nlm.nih.gov/entrez/query.fcgi?CMD=search&DB=gene&term=ECK2307) | -2.780685 |
| [B1757](http://www.ncbi.nlm.nih.gov/entrez/query.fcgi?CMD=search&DB=gene&term=B1757) | [ECK1755](http://www.ncbi.nlm.nih.gov/entrez/query.fcgi?CMD=search&DB=gene&term=ECK1755) | -2.397927 |
| [YieK](http://www.ncbi.nlm.nih.gov/entrez/query.fcgi?CMD=search&DB=gene&term=YIEK) | [ECK3711](http://www.ncbi.nlm.nih.gov/entrez/query.fcgi?CMD=search&DB=gene&term=ECK3711) | -3.298330 |
| [ydjA](http://www.ncbi.nlm.nih.gov/entrez/query.fcgi?CMD=search&DB=gene&term=YDJA) | [ECK1763](http://www.ncbi.nlm.nih.gov/entrez/query.fcgi?CMD=search&DB=gene&term=ECK1763) | -1.844669 |
| [ydbA_2](http://www.ncbi.nlm.nih.gov/entrez/query.fcgi?CMD=search&DB=gene&term=YDBA_2) | [ECK1398](http://www.ncbi.nlm.nih.gov/entrez/query.fcgi?CMD=search&DB=gene&term=ECK1398) | -1.672328 |
| [smg](http://www.ncbi.nlm.nih.gov/entrez/query.fcgi?CMD=search&DB=gene&term=SMG) | [ECK3271](http://www.ncbi.nlm.nih.gov/entrez/query.fcgi?CMD=search&DB=gene&term=ECK3271) | -1.907280 |
| [gloA](http://www.ncbi.nlm.nih.gov/entrez/query.fcgi?CMD=search&DB=gene&term=GLOA) | [ECK1647](http://www.ncbi.nlm.nih.gov/entrez/query.fcgi?CMD=search&DB=gene&term=ECK1647) | -1.550147 |
| [B2511](http://www.ncbi.nlm.nih.gov/entrez/query.fcgi?CMD=search&DB=gene&term=B2511) | [ECK2507](http://www.ncbi.nlm.nih.gov/entrez/query.fcgi?CMD=search&DB=gene&term=ECK2507) | -1.501875 |
| [topB](http://www.ncbi.nlm.nih.gov/entrez/query.fcgi?CMD=search&DB=gene&term=TOPB) | [ECK1761](http://www.ncbi.nlm.nih.gov/entrez/query.fcgi?CMD=search&DB=gene&term=ECK1761) | -2.697226 |
| [yhjK](http://www.ncbi.nlm.nih.gov/entrez/query.fcgi?CMD=search&DB=gene&term=YHJK) | [ECK3514](http://www.ncbi.nlm.nih.gov/entrez/query.fcgi?CMD=search&DB=gene&term=ECK3514) | -1.506791 |
| [yi41](http://www.ncbi.nlm.nih.gov/entrez/query.fcgi?CMD=search&DB=gene&term=YI41) | [ECK4268](http://www.ncbi.nlm.nih.gov/entrez/query.fcgi?CMD=search&DB=gene&term=ECK4268) | -1.715660 |
| [parE](http://www.ncbi.nlm.nih.gov/entrez/query.fcgi?CMD=search&DB=gene&term=PARE) | [ECK3021](http://www.ncbi.nlm.nih.gov/entrez/query.fcgi?CMD=search&DB=gene&term=ECK3021) | -1.925272 |
| [yagF](http://www.ncbi.nlm.nih.gov/entrez/query.fcgi?CMD=search&DB=gene&term=YAGF) | [ECK0270](http://www.ncbi.nlm.nih.gov/entrez/query.fcgi?CMD=search&DB=gene&term=ECK0270) | -1.536311 |
| [yaiB](http://www.ncbi.nlm.nih.gov/entrez/query.fcgi?CMD=search&DB=gene&term=YAIB) | [ECK0377](http://www.ncbi.nlm.nih.gov/entrez/query.fcgi?CMD=search&DB=gene&term=ECK0377) | -1.991787 |
| [yciL](http://www.ncbi.nlm.nih.gov/entrez/query.fcgi?CMD=search&DB=gene&term=YCIL) | [ECK1263](http://www.ncbi.nlm.nih.gov/entrez/query.fcgi?CMD=search&DB=gene&term=ECK1263) | -3.205161 |
| [hisD](http://www.ncbi.nlm.nih.gov/entrez/query.fcgi?CMD=search&DB=gene&term=HISD) | [ECK2015](http://www.ncbi.nlm.nih.gov/entrez/query.fcgi?CMD=search&DB=gene&term=ECK2015) | -2.201710 |
| [galE](http://www.ncbi.nlm.nih.gov/entrez/query.fcgi?CMD=search&DB=gene&term=GALE) | [ECK0748](http://www.ncbi.nlm.nih.gov/entrez/query.fcgi?CMD=search&DB=gene&term=ECK0748) | -2.709263 |
| [pepP](http://www.ncbi.nlm.nih.gov/entrez/query.fcgi?CMD=search&DB=gene&term=PEPP) | [ECK2903](http://www.ncbi.nlm.nih.gov/entrez/query.fcgi?CMD=search&DB=gene&term=ECK2903) | -1.729436 |
| [mdh](http://www.ncbi.nlm.nih.gov/entrez/query.fcgi?CMD=search&DB=gene&term=MDH) | [ECK3225](http://www.ncbi.nlm.nih.gov/entrez/query.fcgi?CMD=search&DB=gene&term=ECK3225) | -2.822397 |
| [asd](http://www.ncbi.nlm.nih.gov/entrez/query.fcgi?CMD=search&DB=gene&term=ASD) | [ECK3419](http://www.ncbi.nlm.nih.gov/entrez/query.fcgi?CMD=search&DB=gene&term=ECK3419) | -1.886412 |
| [yhcM](http://www.ncbi.nlm.nih.gov/entrez/query.fcgi?CMD=search&DB=gene&term=YHCM) | [ECK3221](http://www.ncbi.nlm.nih.gov/entrez/query.fcgi?CMD=search&DB=gene&term=ECK3221) | -2.462311 |
| [spy](http://www.ncbi.nlm.nih.gov/entrez/query.fcgi?CMD=search&DB=gene&term=SPY) | [ECK1741](http://www.ncbi.nlm.nih.gov/entrez/query.fcgi?CMD=search&DB=gene&term=ECK1741) | -1.654387 |
| [yjiT](http://www.ncbi.nlm.nih.gov/entrez/query.fcgi?CMD=search&DB=gene&term=YJIT) | [ECK4333](http://www.ncbi.nlm.nih.gov/entrez/query.fcgi?CMD=search&DB=gene&term=ECK4333) | -1.503364 |
| [yahD](http://www.ncbi.nlm.nih.gov/entrez/query.fcgi?CMD=search&DB=gene&term=YAHD) | [ECK0316](http://www.ncbi.nlm.nih.gov/entrez/query.fcgi?CMD=search&DB=gene&term=ECK0316) | -1.649484 |
| [yicN](http://www.ncbi.nlm.nih.gov/entrez/query.fcgi?CMD=search&DB=gene&term=YICN) | [ECK3654](http://www.ncbi.nlm.nih.gov/entrez/query.fcgi?CMD=search&DB=gene&term=ECK3654) | -2.315268 |
| [mhpF](http://www.ncbi.nlm.nih.gov/entrez/query.fcgi?CMD=search&DB=gene&term=MHPF) | [ECK0348](http://www.ncbi.nlm.nih.gov/entrez/query.fcgi?CMD=search&DB=gene&term=ECK0348) | -1.860893 |
| [ylbE](http://www.ncbi.nlm.nih.gov/entrez/query.fcgi?CMD=search&DB=gene&term=YLBE) | [ECK0512](http://www.ncbi.nlm.nih.gov/entrez/query.fcgi?CMD=search&DB=gene&term=ECK0512) | -1.670205 |
| [racC](http://www.ncbi.nlm.nih.gov/entrez/query.fcgi?CMD=search&DB=gene&term=RACC) | [ECK1348](http://www.ncbi.nlm.nih.gov/entrez/query.fcgi?CMD=search&DB=gene&term=ECK1348) | -2.335120 |
| [yihT](http://www.ncbi.nlm.nih.gov/entrez/query.fcgi?CMD=search&DB=gene&term=YIHT) | [ECK3874](http://www.ncbi.nlm.nih.gov/entrez/query.fcgi?CMD=search&DB=gene&term=ECK3874) | -1.652700 |
| [yagK](http://www.ncbi.nlm.nih.gov/entrez/query.fcgi?CMD=search&DB=gene&term=YAGK) | [ECK0276](http://www.ncbi.nlm.nih.gov/entrez/query.fcgi?CMD=search&DB=gene&term=ECK0276) | -2.529059 |
| [eco](http://www.ncbi.nlm.nih.gov/entrez/query.fcgi?CMD=search&DB=gene&term=ECO) | [ECK2201](http://www.ncbi.nlm.nih.gov/entrez/query.fcgi?CMD=search&DB=gene&term=ECK2201) | -3.318490 |
| [ychB](http://www.ncbi.nlm.nih.gov/entrez/query.fcgi?CMD=search&DB=gene&term=YCHB) | [ECK1196](http://www.ncbi.nlm.nih.gov/entrez/query.fcgi?CMD=search&DB=gene&term=ECK1196) | -1.566236 |
| [tmk](http://www.ncbi.nlm.nih.gov/entrez/query.fcgi?CMD=search&DB=gene&term=TMK) | [ECK1084](http://www.ncbi.nlm.nih.gov/entrez/query.fcgi?CMD=search&DB=gene&term=ECK1084) | -1.757615 |
| [yidW](http://www.ncbi.nlm.nih.gov/entrez/query.fcgi?CMD=search&DB=gene&term=YIDW) | [ECK3687](http://www.ncbi.nlm.nih.gov/entrez/query.fcgi?CMD=search&DB=gene&term=ECK3687) | -2.818164 |
| [topA](http://www.ncbi.nlm.nih.gov/entrez/query.fcgi?CMD=search&DB=gene&term=TOPA) | [ECK1268](http://www.ncbi.nlm.nih.gov/entrez/query.fcgi?CMD=search&DB=gene&term=ECK1268) | -2.014914 |
| [frr](http://www.ncbi.nlm.nih.gov/entrez/query.fcgi?CMD=search&DB=gene&term=FRR) | [ECK0171](http://www.ncbi.nlm.nih.gov/entrez/query.fcgi?CMD=search&DB=gene&term=ECK0171) | -1.705872 |
| [yeeA](http://www.ncbi.nlm.nih.gov/entrez/query.fcgi?CMD=search&DB=gene&term=YEEA) | [ECK2002](http://www.ncbi.nlm.nih.gov/entrez/query.fcgi?CMD=search&DB=gene&term=ECK2002) | -2.507008 |
| [yghA](http://www.ncbi.nlm.nih.gov/entrez/query.fcgi?CMD=search&DB=gene&term=YGHA) | [ECK2997](http://www.ncbi.nlm.nih.gov/entrez/query.fcgi?CMD=search&DB=gene&term=ECK2997) | -1.882122 |
| [qor](http://www.ncbi.nlm.nih.gov/entrez/query.fcgi?CMD=search&DB=gene&term=QOR) | [ECK4043](http://www.ncbi.nlm.nih.gov/entrez/query.fcgi?CMD=search&DB=gene&term=ECK4043) | -1.701969 |
| [yhiQ](http://www.ncbi.nlm.nih.gov/entrez/query.fcgi?CMD=search&DB=gene&term=YHIQ) | [ECK3482](http://www.ncbi.nlm.nih.gov/entrez/query.fcgi?CMD=search&DB=gene&term=ECK3482) | -1.564663 |
| [hisA](http://www.ncbi.nlm.nih.gov/entrez/query.fcgi?CMD=search&DB=gene&term=HISA) | [ECK2019](http://www.ncbi.nlm.nih.gov/entrez/query.fcgi?CMD=search&DB=gene&term=ECK2019) | -1.638739 |
| [mscL](http://www.ncbi.nlm.nih.gov/entrez/query.fcgi?CMD=search&DB=gene&term=MSCL) | [ECK3277](http://www.ncbi.nlm.nih.gov/entrez/query.fcgi?CMD=search&DB=gene&term=ECK3277) | -3.277363 |
| [glnA](http://www.ncbi.nlm.nih.gov/entrez/query.fcgi?CMD=search&DB=gene&term=GLNA) | [ECK3863](http://www.ncbi.nlm.nih.gov/entrez/query.fcgi?CMD=search&DB=gene&term=ECK3863) | -1.831054 |
| [B1955](http://www.ncbi.nlm.nih.gov/entrez/query.fcgi?CMD=search&DB=gene&term=B1955) | [ECK1953](http://www.ncbi.nlm.nih.gov/entrez/query.fcgi?CMD=search&DB=gene&term=ECK1953) | -1.602177 |
| [yiaE](http://www.ncbi.nlm.nih.gov/entrez/query.fcgi?CMD=search&DB=gene&term=YIAE) | [ECK3540](http://www.ncbi.nlm.nih.gov/entrez/query.fcgi?CMD=search&DB=gene&term=ECK3540) | -2.208559 |
| [narY](http://www.ncbi.nlm.nih.gov/entrez/query.fcgi?CMD=search&DB=gene&term=NARY) | [ECK1461](http://www.ncbi.nlm.nih.gov/entrez/query.fcgi?CMD=search&DB=gene&term=ECK1461) | -1.706379 |
| [CmtB](http://www.ncbi.nlm.nih.gov/entrez/query.fcgi?CMD=search&DB=gene&term=CMTB) | [ECK2929](http://www.ncbi.nlm.nih.gov/entrez/query.fcgi?CMD=search&DB=gene&term=ECK2929) | -2.085594 |
| [ilvI](http://www.ncbi.nlm.nih.gov/entrez/query.fcgi?CMD=search&DB=gene&term=ILVI) | [ECK0079](http://www.ncbi.nlm.nih.gov/entrez/query.fcgi?CMD=search&DB=gene&term=ECK0079) | -2.113207 |
| [chaA-R](http://www.ncbi.nlm.nih.gov/entrez/query.fcgi?CMD=search&DB=gene&term=CHAA-R) | [ECK1210](http://www.ncbi.nlm.nih.gov/entrez/query.fcgi?CMD=search&DB=gene&term=ECK1210) | -2.553787 |
| [B2873](http://www.ncbi.nlm.nih.gov/entrez/query.fcgi?CMD=search&DB=gene&term=B2873) | [ECK2869](http://www.ncbi.nlm.nih.gov/entrez/query.fcgi?CMD=search&DB=gene&term=ECK2869) | -1.889365 |
| [B1963](http://www.ncbi.nlm.nih.gov/entrez/query.fcgi?CMD=search&DB=gene&term=B1963) | [ECK1961](http://www.ncbi.nlm.nih.gov/entrez/query.fcgi?CMD=search&DB=gene&term=ECK1961) | -1.880072 |
| [gyrA](http://www.ncbi.nlm.nih.gov/entrez/query.fcgi?CMD=search&DB=gene&term=GYRA) | [ECK2223](http://www.ncbi.nlm.nih.gov/entrez/query.fcgi?CMD=search&DB=gene&term=ECK2223) | -1.828661 |
| [ygiB](http://www.ncbi.nlm.nih.gov/entrez/query.fcgi?CMD=search&DB=gene&term=YGIB) | [ECK3028](http://www.ncbi.nlm.nih.gov/entrez/query.fcgi?CMD=search&DB=gene&term=ECK3028) | -1.714242 |
| [B1980](http://www.ncbi.nlm.nih.gov/entrez/query.fcgi?CMD=search&DB=gene&term=B1980) | [ECK1975](http://www.ncbi.nlm.nih.gov/entrez/query.fcgi?CMD=search&DB=gene&term=ECK1975) | -1.599249 |
| [proP](http://www.ncbi.nlm.nih.gov/entrez/query.fcgi?CMD=search&DB=gene&term=PROP) | [ECK4104](http://www.ncbi.nlm.nih.gov/entrez/query.fcgi?CMD=search&DB=gene&term=ECK4104) | -1.811810 |
| [yiaO](http://www.ncbi.nlm.nih.gov/entrez/query.fcgi?CMD=search&DB=gene&term=YIAO) | [ECK3568](http://www.ncbi.nlm.nih.gov/entrez/query.fcgi?CMD=search&DB=gene&term=ECK3568) | -2.230370 |
| [B2650](http://www.ncbi.nlm.nih.gov/entrez/query.fcgi?CMD=search&DB=gene&term=B2650) | [ECK2647](http://www.ncbi.nlm.nih.gov/entrez/query.fcgi?CMD=search&DB=gene&term=ECK2647) | -1.933651 |
| [yljA](http://www.ncbi.nlm.nih.gov/entrez/query.fcgi?CMD=search&DB=gene&term=YLJA) | [ECK0872](http://www.ncbi.nlm.nih.gov/entrez/query.fcgi?CMD=search&DB=gene&term=ECK0872) | -2.354350 |
| [ycfN](http://www.ncbi.nlm.nih.gov/entrez/query.fcgi?CMD=search&DB=gene&term=YCFN) | [ECK1092](http://www.ncbi.nlm.nih.gov/entrez/query.fcgi?CMD=search&DB=gene&term=ECK1092) | -2.432220 |
| [B1432](http://www.ncbi.nlm.nih.gov/entrez/query.fcgi?CMD=search&DB=gene&term=B1432) | [ECK1426](http://www.ncbi.nlm.nih.gov/entrez/query.fcgi?CMD=search&DB=gene&term=ECK1426) | -2.397101 |
| [chaA](http://www.ncbi.nlm.nih.gov/entrez/query.fcgi?CMD=search&DB=gene&term=CHAA) | [ECK1210](http://www.ncbi.nlm.nih.gov/entrez/query.fcgi?CMD=search&DB=gene&term=ECK1210) | -2.498427 |
| [yidP](http://www.ncbi.nlm.nih.gov/entrez/query.fcgi?CMD=search&DB=gene&term=YIDP) | [ECK3676](http://www.ncbi.nlm.nih.gov/entrez/query.fcgi?CMD=search&DB=gene&term=ECK3676) | -2.557285 |
| [prpB](http://www.ncbi.nlm.nih.gov/entrez/query.fcgi?CMD=search&DB=gene&term=PRPB) | [ECK0329](http://www.ncbi.nlm.nih.gov/entrez/query.fcgi?CMD=search&DB=gene&term=ECK0329) | -1.611275 |
| [ahpF](http://www.ncbi.nlm.nih.gov/entrez/query.fcgi?CMD=search&DB=gene&term=AHPF) | [ECK0600](http://www.ncbi.nlm.nih.gov/entrez/query.fcgi?CMD=search&DB=gene&term=ECK0600) | -2.320441 |
| [yciH](http://www.ncbi.nlm.nih.gov/entrez/query.fcgi?CMD=search&DB=gene&term=YCIH) | [ECK1277](http://www.ncbi.nlm.nih.gov/entrez/query.fcgi?CMD=search&DB=gene&term=ECK1277) | -1.950905 |
| [flhA](http://www.ncbi.nlm.nih.gov/entrez/query.fcgi?CMD=search&DB=gene&term=FLHA) | [ECK1880](http://www.ncbi.nlm.nih.gov/entrez/query.fcgi?CMD=search&DB=gene&term=ECK1880) | -1.738556 |
| [ycaH](http://www.ncbi.nlm.nih.gov/entrez/query.fcgi?CMD=search&DB=gene&term=YCAH) | [ECK0906](http://www.ncbi.nlm.nih.gov/entrez/query.fcgi?CMD=search&DB=gene&term=ECK0906) | -2.019557 |
| [ydeD](http://www.ncbi.nlm.nih.gov/entrez/query.fcgi?CMD=search&DB=gene&term=YDED) | [ECK1526](http://www.ncbi.nlm.nih.gov/entrez/query.fcgi?CMD=search&DB=gene&term=ECK1526) | -2.021874 |
