# Supplementary material for: Datasets for transcriptomics, q-proteomics and phenotype microarrays of polyphosphate metabolism mutants from Escherichia coli
Source: Data Brief. 2017 Mar 18;12:13–7. doi: 10.1016/j.dib.2017.03.010 (PMC5367803; doi:10.1016/j.dib.2017.03.010)
Supplement: Supplementary file 2 — Supplementary material [file mmc2.zip › BBAGEN-16-480/Wt vs ppx marrays.docx]

| **ID** | **Symdesc** | **Zscore** |
| --- | --- | --- |
| [ydeB](http://www.ncbi.nlm.nih.gov/entrez/query.fcgi?CMD=search&DB=gene&term=YDEB) | [ECK1522](http://www.ncbi.nlm.nih.gov/entrez/query.fcgi?CMD=search&DB=gene&term=ECK1522) | 2.553394 |
| [yiaA](http://www.ncbi.nlm.nih.gov/entrez/query.fcgi?CMD=search&DB=gene&term=YIAA) | [ECK3551](http://www.ncbi.nlm.nih.gov/entrez/query.fcgi?CMD=search&DB=gene&term=ECK3551) | 3.161879 |
| [yiaY](http://www.ncbi.nlm.nih.gov/entrez/query.fcgi?CMD=search&DB=gene&term=YIAY) | [ECK3578](http://www.ncbi.nlm.nih.gov/entrez/query.fcgi?CMD=search&DB=gene&term=ECK3578) | 1.809596 |
| [deaD-R](http://www.ncbi.nlm.nih.gov/entrez/query.fcgi?CMD=search&DB=gene&term=DEAD-R) | [ECK3150](http://www.ncbi.nlm.nih.gov/entrez/query.fcgi?CMD=search&DB=gene&term=ECK3150) | 2.495808 |
| [B2145](http://www.ncbi.nlm.nih.gov/entrez/query.fcgi?CMD=search&DB=gene&term=B2145) | [ECK2138](http://www.ncbi.nlm.nih.gov/entrez/query.fcgi?CMD=search&DB=gene&term=ECK2138) | 2.058980 |
| [ydeI](http://www.ncbi.nlm.nih.gov/entrez/query.fcgi?CMD=search&DB=gene&term=YDEI) | [ECK1529](http://www.ncbi.nlm.nih.gov/entrez/query.fcgi?CMD=search&DB=gene&term=ECK1529) | 1.781732 |
| [B3021](http://www.ncbi.nlm.nih.gov/entrez/query.fcgi?CMD=search&DB=gene&term=B3021) | [ECK3012](http://www.ncbi.nlm.nih.gov/entrez/query.fcgi?CMD=search&DB=gene&term=ECK3012) | 1.981964 |
| [wcaD](http://www.ncbi.nlm.nih.gov/entrez/query.fcgi?CMD=search&DB=gene&term=WCAD) | [ECK2050](http://www.ncbi.nlm.nih.gov/entrez/query.fcgi?CMD=search&DB=gene&term=ECK2050) | 1.819425 |
| [deaD](http://www.ncbi.nlm.nih.gov/entrez/query.fcgi?CMD=search&DB=gene&term=DEAD) | [ECK3150](http://www.ncbi.nlm.nih.gov/entrez/query.fcgi?CMD=search&DB=gene&term=ECK3150) | 2.282447 |
| [yhiX](http://www.ncbi.nlm.nih.gov/entrez/query.fcgi?CMD=search&DB=gene&term=YHIX) | [ECK3501](http://www.ncbi.nlm.nih.gov/entrez/query.fcgi?CMD=search&DB=gene&term=ECK3501) | 4.249161 |
| [yjiM](http://www.ncbi.nlm.nih.gov/entrez/query.fcgi?CMD=search&DB=gene&term=YJIM) | [ECK4326](http://www.ncbi.nlm.nih.gov/entrez/query.fcgi?CMD=search&DB=gene&term=ECK4326) | 2.639465 |
| [ldhA](http://www.ncbi.nlm.nih.gov/entrez/query.fcgi?CMD=search&DB=gene&term=LDHA) | [ECK1377](http://www.ncbi.nlm.nih.gov/entrez/query.fcgi?CMD=search&DB=gene&term=ECK1377) | 1.503310 |
| [hyfG](http://www.ncbi.nlm.nih.gov/entrez/query.fcgi?CMD=search&DB=gene&term=HYFG) | [ECK2483](http://www.ncbi.nlm.nih.gov/entrez/query.fcgi?CMD=search&DB=gene&term=ECK2483) | 1.519283 |
| [farR](http://www.ncbi.nlm.nih.gov/entrez/query.fcgi?CMD=search&DB=gene&term=FARR) | [ECK0718](http://www.ncbi.nlm.nih.gov/entrez/query.fcgi?CMD=search&DB=gene&term=ECK0718) | 1.547947 |
| [ydjY](http://www.ncbi.nlm.nih.gov/entrez/query.fcgi?CMD=search&DB=gene&term=YDJY) | [ECK1749](http://www.ncbi.nlm.nih.gov/entrez/query.fcgi?CMD=search&DB=gene&term=ECK1749) | 1.523610 |
| [yhiE](http://www.ncbi.nlm.nih.gov/entrez/query.fcgi?CMD=search&DB=gene&term=YHIE) | [ECK3496](http://www.ncbi.nlm.nih.gov/entrez/query.fcgi?CMD=search&DB=gene&term=ECK3496) | 4.575320 |
| [hlyE](http://www.ncbi.nlm.nih.gov/entrez/query.fcgi?CMD=search&DB=gene&term=HLYE) | [ECK1170](http://www.ncbi.nlm.nih.gov/entrez/query.fcgi?CMD=search&DB=gene&term=ECK1170) | 1.758001 |
| [murB](http://www.ncbi.nlm.nih.gov/entrez/query.fcgi?CMD=search&DB=gene&term=MURB) | [ECK3964](http://www.ncbi.nlm.nih.gov/entrez/query.fcgi?CMD=search&DB=gene&term=ECK3964) | 1.595150 |
| [ybeZ](http://www.ncbi.nlm.nih.gov/entrez/query.fcgi?CMD=search&DB=gene&term=YBEZ) | [ECK0652](http://www.ncbi.nlm.nih.gov/entrez/query.fcgi?CMD=search&DB=gene&term=ECK0652) | 2.136703 |
| [nmpC](http://www.ncbi.nlm.nih.gov/entrez/query.fcgi?CMD=search&DB=gene&term=NMPC) | [ECK0544](http://www.ncbi.nlm.nih.gov/entrez/query.fcgi?CMD=search&DB=gene&term=ECK0544) | 4.466373 |
| [yhcJ](http://www.ncbi.nlm.nih.gov/entrez/query.fcgi?CMD=search&DB=gene&term=YHCJ) | [ECK3212](http://www.ncbi.nlm.nih.gov/entrez/query.fcgi?CMD=search&DB=gene&term=ECK3212) | 1.657406 |
| [hdeD](http://www.ncbi.nlm.nih.gov/entrez/query.fcgi?CMD=search&DB=gene&term=HDED) | [ECK3495](http://www.ncbi.nlm.nih.gov/entrez/query.fcgi?CMD=search&DB=gene&term=ECK3495) | 1.998314 |
| [tdcB](http://www.ncbi.nlm.nih.gov/entrez/query.fcgi?CMD=search&DB=gene&term=TDCB) | [ECK3106](http://www.ncbi.nlm.nih.gov/entrez/query.fcgi?CMD=search&DB=gene&term=ECK3106) | 4.554219 |
| [emrD](http://www.ncbi.nlm.nih.gov/entrez/query.fcgi?CMD=search&DB=gene&term=EMRD) | [ECK3664](http://www.ncbi.nlm.nih.gov/entrez/query.fcgi?CMD=search&DB=gene&term=ECK3664) | 1.516012 |
| [yccF](http://www.ncbi.nlm.nih.gov/entrez/query.fcgi?CMD=search&DB=gene&term=YCCF) | [ECK0952](http://www.ncbi.nlm.nih.gov/entrez/query.fcgi?CMD=search&DB=gene&term=ECK0952) | 2.025280 |
| [phnO](http://www.ncbi.nlm.nih.gov/entrez/query.fcgi?CMD=search&DB=gene&term=PHNO) | [ECK4086](http://www.ncbi.nlm.nih.gov/entrez/query.fcgi?CMD=search&DB=gene&term=ECK4086) | 1.677435 |
| [cadA](http://www.ncbi.nlm.nih.gov/entrez/query.fcgi?CMD=search&DB=gene&term=CADA) | [ECK4125](http://www.ncbi.nlm.nih.gov/entrez/query.fcgi?CMD=search&DB=gene&term=ECK4125) | 1.635007 |
| [ygjR](http://www.ncbi.nlm.nih.gov/entrez/query.fcgi?CMD=search&DB=gene&term=YGJR) | [ECK3077](http://www.ncbi.nlm.nih.gov/entrez/query.fcgi?CMD=search&DB=gene&term=ECK3077) | 3.297521 |
| [flgA](http://www.ncbi.nlm.nih.gov/entrez/query.fcgi?CMD=search&DB=gene&term=FLGA) | [ECK1057](http://www.ncbi.nlm.nih.gov/entrez/query.fcgi?CMD=search&DB=gene&term=ECK1057) | 2.444282 |
| [gapC_1](http://www.ncbi.nlm.nih.gov/entrez/query.fcgi?CMD=search&DB=gene&term=GAPC_1) | [ECK1409](http://www.ncbi.nlm.nih.gov/entrez/query.fcgi?CMD=search&DB=gene&term=ECK1409) | 2.788965 |
| [ygiA](http://www.ncbi.nlm.nih.gov/entrez/query.fcgi?CMD=search&DB=gene&term=YGIA) | [ECK3027](http://www.ncbi.nlm.nih.gov/entrez/query.fcgi?CMD=search&DB=gene&term=ECK3027) | 3.946951 |
| [ybeV](http://www.ncbi.nlm.nih.gov/entrez/query.fcgi?CMD=search&DB=gene&term=YBEV) | [ECK0642](http://www.ncbi.nlm.nih.gov/entrez/query.fcgi?CMD=search&DB=gene&term=ECK0642) | 1.798562 |
| [B1163](http://www.ncbi.nlm.nih.gov/entrez/query.fcgi?CMD=search&DB=gene&term=B1163) | [ECK1150](http://www.ncbi.nlm.nih.gov/entrez/query.fcgi?CMD=search&DB=gene&term=ECK1150) | 1.641691 |
| [wecC](http://www.ncbi.nlm.nih.gov/entrez/query.fcgi?CMD=search&DB=gene&term=WECC) | [ECK3779](http://www.ncbi.nlm.nih.gov/entrez/query.fcgi?CMD=search&DB=gene&term=ECK3779) | 2.130529 |
| [polB](http://www.ncbi.nlm.nih.gov/entrez/query.fcgi?CMD=search&DB=gene&term=POLB) | [ECK0061](http://www.ncbi.nlm.nih.gov/entrez/query.fcgi?CMD=search&DB=gene&term=ECK0061) | 5.717198 |
| [B1806](http://www.ncbi.nlm.nih.gov/entrez/query.fcgi?CMD=search&DB=gene&term=B1806) | [ECK1804](http://www.ncbi.nlm.nih.gov/entrez/query.fcgi?CMD=search&DB=gene&term=ECK1804) | 2.020121 |
| [rlpB](http://www.ncbi.nlm.nih.gov/entrez/query.fcgi?CMD=search&DB=gene&term=RLPB) | [ECK0634](http://www.ncbi.nlm.nih.gov/entrez/query.fcgi?CMD=search&DB=gene&term=ECK0634) | 2.314503 |
| [yejF](http://www.ncbi.nlm.nih.gov/entrez/query.fcgi?CMD=search&DB=gene&term=YEJF) | [ECK2174](http://www.ncbi.nlm.nih.gov/entrez/query.fcgi?CMD=search&DB=gene&term=ECK2174) | 1.741527 |
| [B1565](http://www.ncbi.nlm.nih.gov/entrez/query.fcgi?CMD=search&DB=gene&term=B1565) | [ECK1559](http://www.ncbi.nlm.nih.gov/entrez/query.fcgi?CMD=search&DB=gene&term=ECK1559) | 1.659278 |
| [B1762](http://www.ncbi.nlm.nih.gov/entrez/query.fcgi?CMD=search&DB=gene&term=B1762) | [ECK1760](http://www.ncbi.nlm.nih.gov/entrez/query.fcgi?CMD=search&DB=gene&term=ECK1760) | 1.664023 |
| [ybjE](http://www.ncbi.nlm.nih.gov/entrez/query.fcgi?CMD=search&DB=gene&term=YBJE) | [ECK0865](http://www.ncbi.nlm.nih.gov/entrez/query.fcgi?CMD=search&DB=gene&term=ECK0865) | 2.977527 |
| [ymcB](http://www.ncbi.nlm.nih.gov/entrez/query.fcgi?CMD=search&DB=gene&term=YMCB) | [ECK0976](http://www.ncbi.nlm.nih.gov/entrez/query.fcgi?CMD=search&DB=gene&term=ECK0976) | 1.872606 |
| [nrdA](http://www.ncbi.nlm.nih.gov/entrez/query.fcgi?CMD=search&DB=gene&term=NRDA) | [ECK2226](http://www.ncbi.nlm.nih.gov/entrez/query.fcgi?CMD=search&DB=gene&term=ECK2226) | 1.792871 |
| [B1724](http://www.ncbi.nlm.nih.gov/entrez/query.fcgi?CMD=search&DB=gene&term=B1724) | [ECK1722](http://www.ncbi.nlm.nih.gov/entrez/query.fcgi?CMD=search&DB=gene&term=ECK1722) | 1.500757 |
| [yliI](http://www.ncbi.nlm.nih.gov/entrez/query.fcgi?CMD=search&DB=gene&term=YLII) | [ECK0827](http://www.ncbi.nlm.nih.gov/entrez/query.fcgi?CMD=search&DB=gene&term=ECK0827) | 1.536252 |
| [lytB](http://www.ncbi.nlm.nih.gov/entrez/query.fcgi?CMD=search&DB=gene&term=LYTB) | [ECK0030](http://www.ncbi.nlm.nih.gov/entrez/query.fcgi?CMD=search&DB=gene&term=ECK0030) | 2.012317 |
| [hdeB](http://www.ncbi.nlm.nih.gov/entrez/query.fcgi?CMD=search&DB=gene&term=HDEB) | [ECK3493](http://www.ncbi.nlm.nih.gov/entrez/query.fcgi?CMD=search&DB=gene&term=ECK3493) | 5.732525 |
| [elaA](http://www.ncbi.nlm.nih.gov/entrez/query.fcgi?CMD=search&DB=gene&term=ELAA) | [ECK2261](http://www.ncbi.nlm.nih.gov/entrez/query.fcgi?CMD=search&DB=gene&term=ECK2261) | 1.537929 |
| [ydaL](http://www.ncbi.nlm.nih.gov/entrez/query.fcgi?CMD=search&DB=gene&term=YDAL) | [ECK1337](http://www.ncbi.nlm.nih.gov/entrez/query.fcgi?CMD=search&DB=gene&term=ECK1337) | 2.367209 |
| [yrbI](http://www.ncbi.nlm.nih.gov/entrez/query.fcgi?CMD=search&DB=gene&term=YRBI) | [ECK3187](http://www.ncbi.nlm.nih.gov/entrez/query.fcgi?CMD=search&DB=gene&term=ECK3187) | 2.005979 |
| [yhiW](http://www.ncbi.nlm.nih.gov/entrez/query.fcgi?CMD=search&DB=gene&term=YHIW) | [ECK3499](http://www.ncbi.nlm.nih.gov/entrez/query.fcgi?CMD=search&DB=gene&term=ECK3499) | 2.112225 |
| [ygjN](http://www.ncbi.nlm.nih.gov/entrez/query.fcgi?CMD=search&DB=gene&term=YGJN) | [ECK3073](http://www.ncbi.nlm.nih.gov/entrez/query.fcgi?CMD=search&DB=gene&term=ECK3073) | 2.015857 |
| [pgpA](http://www.ncbi.nlm.nih.gov/entrez/query.fcgi?CMD=search&DB=gene&term=PGPA) | [ECK0412](http://www.ncbi.nlm.nih.gov/entrez/query.fcgi?CMD=search&DB=gene&term=ECK0412) | 1.733984 |
| [yegX](http://www.ncbi.nlm.nih.gov/entrez/query.fcgi?CMD=search&DB=gene&term=YEGX) | [ECK2095](http://www.ncbi.nlm.nih.gov/entrez/query.fcgi?CMD=search&DB=gene&term=ECK2095) | 2.825102 |
| [B2001](http://www.ncbi.nlm.nih.gov/entrez/query.fcgi?CMD=search&DB=gene&term=B2001) | [ECK1994](http://www.ncbi.nlm.nih.gov/entrez/query.fcgi?CMD=search&DB=gene&term=ECK1994) | 1.811636 |
| [hupA-R](http://www.ncbi.nlm.nih.gov/entrez/query.fcgi?CMD=search&DB=gene&term=HUPA-R) | [ECK3992](http://www.ncbi.nlm.nih.gov/entrez/query.fcgi?CMD=search&DB=gene&term=ECK3992) | 2.204576 |
| [hfq](http://www.ncbi.nlm.nih.gov/entrez/query.fcgi?CMD=search&DB=gene&term=HFQ) | [ECK4168](http://www.ncbi.nlm.nih.gov/entrez/query.fcgi?CMD=search&DB=gene&term=ECK4168) | 2.191434 |
| [ptsI](http://www.ncbi.nlm.nih.gov/entrez/query.fcgi?CMD=search&DB=gene&term=PTSI) | [ECK2411](http://www.ncbi.nlm.nih.gov/entrez/query.fcgi?CMD=search&DB=gene&term=ECK2411) | 1.710685 |
| [rpmF](http://www.ncbi.nlm.nih.gov/entrez/query.fcgi?CMD=search&DB=gene&term=RPMF) | [ECK1075](http://www.ncbi.nlm.nih.gov/entrez/query.fcgi?CMD=search&DB=gene&term=ECK1075) | 1.914481 |
| [B1472](http://www.ncbi.nlm.nih.gov/entrez/query.fcgi?CMD=search&DB=gene&term=B1472) | [ECK1466](http://www.ncbi.nlm.nih.gov/entrez/query.fcgi?CMD=search&DB=gene&term=ECK1466) | 1.836135 |
| [yabH](http://www.ncbi.nlm.nih.gov/entrez/query.fcgi?CMD=search&DB=gene&term=YABH) | [ECK0056](http://www.ncbi.nlm.nih.gov/entrez/query.fcgi?CMD=search&DB=gene&term=ECK0056) | 2.072036 |
| [B2451](http://www.ncbi.nlm.nih.gov/entrez/query.fcgi?CMD=search&DB=gene&term=B2451) | [ECK2446](http://www.ncbi.nlm.nih.gov/entrez/query.fcgi?CMD=search&DB=gene&term=ECK2446) | 2.168409 |
| [ynfM](http://www.ncbi.nlm.nih.gov/entrez/query.fcgi?CMD=search&DB=gene&term=YNFM) | [ECK1591](http://www.ncbi.nlm.nih.gov/entrez/query.fcgi?CMD=search&DB=gene&term=ECK1591) | 1.703814 |
| [yhdE](http://www.ncbi.nlm.nih.gov/entrez/query.fcgi?CMD=search&DB=gene&term=YHDE) | [ECK3236](http://www.ncbi.nlm.nih.gov/entrez/query.fcgi?CMD=search&DB=gene&term=ECK3236) | 1.753037 |
| [pflB](http://www.ncbi.nlm.nih.gov/entrez/query.fcgi?CMD=search&DB=gene&term=PFLB) | [ECK0894](http://www.ncbi.nlm.nih.gov/entrez/query.fcgi?CMD=search&DB=gene&term=ECK0894) | 2.033942 |
| [ybeF](http://www.ncbi.nlm.nih.gov/entrez/query.fcgi?CMD=search&DB=gene&term=YBEF) | [ECK0622](http://www.ncbi.nlm.nih.gov/entrez/query.fcgi?CMD=search&DB=gene&term=ECK0622) | 1.989502 |
| [clpP](http://www.ncbi.nlm.nih.gov/entrez/query.fcgi?CMD=search&DB=gene&term=CLPP) | [ECK0431](http://www.ncbi.nlm.nih.gov/entrez/query.fcgi?CMD=search&DB=gene&term=ECK0431) | 2.265460 |
| [aceE](http://www.ncbi.nlm.nih.gov/entrez/query.fcgi?CMD=search&DB=gene&term=ACEE) | [ECK0113](http://www.ncbi.nlm.nih.gov/entrez/query.fcgi?CMD=search&DB=gene&term=ECK0113) | 1.720231 |
| [ydfG](http://www.ncbi.nlm.nih.gov/entrez/query.fcgi?CMD=search&DB=gene&term=YDFG) | [ECK1532](http://www.ncbi.nlm.nih.gov/entrez/query.fcgi?CMD=search&DB=gene&term=ECK1532) | 2.283205 |
| [acpP](http://www.ncbi.nlm.nih.gov/entrez/query.fcgi?CMD=search&DB=gene&term=ACPP) | [ECK1080](http://www.ncbi.nlm.nih.gov/entrez/query.fcgi?CMD=search&DB=gene&term=ECK1080) | 1.857267 |
| [lyxK](http://www.ncbi.nlm.nih.gov/entrez/query.fcgi?CMD=search&DB=gene&term=LYXK) | [ECK3569](http://www.ncbi.nlm.nih.gov/entrez/query.fcgi?CMD=search&DB=gene&term=ECK3569) | 1.984142 |
| [hpt](http://www.ncbi.nlm.nih.gov/entrez/query.fcgi?CMD=search&DB=gene&term=HPT) | [ECK0124](http://www.ncbi.nlm.nih.gov/entrez/query.fcgi?CMD=search&DB=gene&term=ECK0124) | 1.579347 |
| [cspB](http://www.ncbi.nlm.nih.gov/entrez/query.fcgi?CMD=search&DB=gene&term=CSPB) | [ECK1551](http://www.ncbi.nlm.nih.gov/entrez/query.fcgi?CMD=search&DB=gene&term=ECK1551) | 1.689517 |
| [cspI](http://www.ncbi.nlm.nih.gov/entrez/query.fcgi?CMD=search&DB=gene&term=CSPI) | [ECK1546](http://www.ncbi.nlm.nih.gov/entrez/query.fcgi?CMD=search&DB=gene&term=ECK1546) | 5.478389 |
| [yihA](http://www.ncbi.nlm.nih.gov/entrez/query.fcgi?CMD=search&DB=gene&term=YIHA) | [ECK3857](http://www.ncbi.nlm.nih.gov/entrez/query.fcgi?CMD=search&DB=gene&term=ECK3857) | 3.276146 |
| [phnL](http://www.ncbi.nlm.nih.gov/entrez/query.fcgi?CMD=search&DB=gene&term=PHNL) | [ECK4089](http://www.ncbi.nlm.nih.gov/entrez/query.fcgi?CMD=search&DB=gene&term=ECK4089) | 1.757245 |
| [nac](http://www.ncbi.nlm.nih.gov/entrez/query.fcgi?CMD=search&DB=gene&term=NAC) | [ECK1983](http://www.ncbi.nlm.nih.gov/entrez/query.fcgi?CMD=search&DB=gene&term=ECK1983) | 1.609030 |
| [ybdO](http://www.ncbi.nlm.nih.gov/entrez/query.fcgi?CMD=search&DB=gene&term=YBDO) | [ECK0597](http://www.ncbi.nlm.nih.gov/entrez/query.fcgi?CMD=search&DB=gene&term=ECK0597) | 1.724240 |
| [B2363](http://www.ncbi.nlm.nih.gov/entrez/query.fcgi?CMD=search&DB=gene&term=B2363) | [ECK2357](http://www.ncbi.nlm.nih.gov/entrez/query.fcgi?CMD=search&DB=gene&term=ECK2357) | 1.534708 |
| [rbsD](http://www.ncbi.nlm.nih.gov/entrez/query.fcgi?CMD=search&DB=gene&term=RBSD) | [ECK3742](http://www.ncbi.nlm.nih.gov/entrez/query.fcgi?CMD=search&DB=gene&term=ECK3742) | 1.917067 |
| [kdgK](http://www.ncbi.nlm.nih.gov/entrez/query.fcgi?CMD=search&DB=gene&term=KDGK) | [ECK3511](http://www.ncbi.nlm.nih.gov/entrez/query.fcgi?CMD=search&DB=gene&term=ECK3511) | 1.634646 |
| [yggS](http://www.ncbi.nlm.nih.gov/entrez/query.fcgi?CMD=search&DB=gene&term=YGGS) | [ECK2946](http://www.ncbi.nlm.nih.gov/entrez/query.fcgi?CMD=search&DB=gene&term=ECK2946) | 1.622883 |
| [ydeK](http://www.ncbi.nlm.nih.gov/entrez/query.fcgi?CMD=search&DB=gene&term=YDEK) | [ECK1503](http://www.ncbi.nlm.nih.gov/entrez/query.fcgi?CMD=search&DB=gene&term=ECK1503) | 2.288664 |
| [adk](http://www.ncbi.nlm.nih.gov/entrez/query.fcgi?CMD=search&DB=gene&term=ADK) | [ECK0468](http://www.ncbi.nlm.nih.gov/entrez/query.fcgi?CMD=search&DB=gene&term=ECK0468) | 1.605790 |
| [yijD](http://www.ncbi.nlm.nih.gov/entrez/query.fcgi?CMD=search&DB=gene&term=YIJD) | [ECK3956](http://www.ncbi.nlm.nih.gov/entrez/query.fcgi?CMD=search&DB=gene&term=ECK3956) | 2.137429 |
| [rpmE](http://www.ncbi.nlm.nih.gov/entrez/query.fcgi?CMD=search&DB=gene&term=RPME) | [ECK3928](http://www.ncbi.nlm.nih.gov/entrez/query.fcgi?CMD=search&DB=gene&term=ECK3928) | 1.864183 |
| [sfsA](http://www.ncbi.nlm.nih.gov/entrez/query.fcgi?CMD=search&DB=gene&term=SFSA) | [ECK0145](http://www.ncbi.nlm.nih.gov/entrez/query.fcgi?CMD=search&DB=gene&term=ECK0145) | 1.833008 |
| [B1631](http://www.ncbi.nlm.nih.gov/entrez/query.fcgi?CMD=search&DB=gene&term=B1631) | [ECK1627](http://www.ncbi.nlm.nih.gov/entrez/query.fcgi?CMD=search&DB=gene&term=ECK1627) | 2.880244 |
| [ndh](http://www.ncbi.nlm.nih.gov/entrez/query.fcgi?CMD=search&DB=gene&term=NDH) | [ECK1095](http://www.ncbi.nlm.nih.gov/entrez/query.fcgi?CMD=search&DB=gene&term=ECK1095) | 2.023623 |
| [yhaR](http://www.ncbi.nlm.nih.gov/entrez/query.fcgi?CMD=search&DB=gene&term=YHAR) | [ECK3102](http://www.ncbi.nlm.nih.gov/entrez/query.fcgi?CMD=search&DB=gene&term=ECK3102) | 5.158855 |
| [yafZ](http://www.ncbi.nlm.nih.gov/entrez/query.fcgi?CMD=search&DB=gene&term=YAFZ) | [ECK0254](http://www.ncbi.nlm.nih.gov/entrez/query.fcgi?CMD=search&DB=gene&term=ECK0254) | 1.999682 |
| [yagJ](http://www.ncbi.nlm.nih.gov/entrez/query.fcgi?CMD=search&DB=gene&term=YAGJ) | [ECK0275](http://www.ncbi.nlm.nih.gov/entrez/query.fcgi?CMD=search&DB=gene&term=ECK0275) | 1.569349 |
| [rpsG](http://www.ncbi.nlm.nih.gov/entrez/query.fcgi?CMD=search&DB=gene&term=RPSG) | [ECK3328](http://www.ncbi.nlm.nih.gov/entrez/query.fcgi?CMD=search&DB=gene&term=ECK3328) | 1.914715 |
| [yagH](http://www.ncbi.nlm.nih.gov/entrez/query.fcgi?CMD=search&DB=gene&term=YAGH) | [ECK0272](http://www.ncbi.nlm.nih.gov/entrez/query.fcgi?CMD=search&DB=gene&term=ECK0272) | 2.026964 |
| [yggF](http://www.ncbi.nlm.nih.gov/entrez/query.fcgi?CMD=search&DB=gene&term=YGGF) | [ECK2926](http://www.ncbi.nlm.nih.gov/entrez/query.fcgi?CMD=search&DB=gene&term=ECK2926) | 1.520099 |
| [yghK](http://www.ncbi.nlm.nih.gov/entrez/query.fcgi?CMD=search&DB=gene&term=YGHK) | [ECK2969](http://www.ncbi.nlm.nih.gov/entrez/query.fcgi?CMD=search&DB=gene&term=ECK2969) | 1.660683 |
| [thrC](http://www.ncbi.nlm.nih.gov/entrez/query.fcgi?CMD=search&DB=gene&term=THRC) | [ECK0004](http://www.ncbi.nlm.nih.gov/entrez/query.fcgi?CMD=search&DB=gene&term=ECK0004) | 1.521617 |
| [cspE](http://www.ncbi.nlm.nih.gov/entrez/query.fcgi?CMD=search&DB=gene&term=CSPE) | [ECK0616](http://www.ncbi.nlm.nih.gov/entrez/query.fcgi?CMD=search&DB=gene&term=ECK0616) | 2.040197 |
| [B2740](http://www.ncbi.nlm.nih.gov/entrez/query.fcgi?CMD=search&DB=gene&term=B2740) | [ECK2735](http://www.ncbi.nlm.nih.gov/entrez/query.fcgi?CMD=search&DB=gene&term=ECK2735) | 1.585534 |
| [phnP](http://www.ncbi.nlm.nih.gov/entrez/query.fcgi?CMD=search&DB=gene&term=PHNP) | [ECK4085](http://www.ncbi.nlm.nih.gov/entrez/query.fcgi?CMD=search&DB=gene&term=ECK4085) | 1.578660 |
| [yjfJ](http://www.ncbi.nlm.nih.gov/entrez/query.fcgi?CMD=search&DB=gene&term=YJFJ) | [ECK4178](http://www.ncbi.nlm.nih.gov/entrez/query.fcgi?CMD=search&DB=gene&term=ECK4178) | 1.574001 |
| [trpD](http://www.ncbi.nlm.nih.gov/entrez/query.fcgi?CMD=search&DB=gene&term=TRPD) | [ECK1257](http://www.ncbi.nlm.nih.gov/entrez/query.fcgi?CMD=search&DB=gene&term=ECK1257) | 1.563395 |
| [yheK](http://www.ncbi.nlm.nih.gov/entrez/query.fcgi?CMD=search&DB=gene&term=YHEK) | [ECK3320](http://www.ncbi.nlm.nih.gov/entrez/query.fcgi?CMD=search&DB=gene&term=ECK3320) | 1.673367 |
| [yidL](http://www.ncbi.nlm.nih.gov/entrez/query.fcgi?CMD=search&DB=gene&term=YIDL) | [ECK3671](http://www.ncbi.nlm.nih.gov/entrez/query.fcgi?CMD=search&DB=gene&term=ECK3671) | 1.566285 |
| [hdeA](http://www.ncbi.nlm.nih.gov/entrez/query.fcgi?CMD=search&DB=gene&term=HDEA) | [ECK3494](http://www.ncbi.nlm.nih.gov/entrez/query.fcgi?CMD=search&DB=gene&term=ECK3494) | 3.365580 |
| [purT](http://www.ncbi.nlm.nih.gov/entrez/query.fcgi?CMD=search&DB=gene&term=PURT) | [ECK1850](http://www.ncbi.nlm.nih.gov/entrez/query.fcgi?CMD=search&DB=gene&term=ECK1850) | 2.497086 |
| [lpxB](http://www.ncbi.nlm.nih.gov/entrez/query.fcgi?CMD=search&DB=gene&term=LPXB) | [ECK0181](http://www.ncbi.nlm.nih.gov/entrez/query.fcgi?CMD=search&DB=gene&term=ECK0181) | 2.261292 |
| [dcm](http://www.ncbi.nlm.nih.gov/entrez/query.fcgi?CMD=search&DB=gene&term=DCM) | [ECK1959](http://www.ncbi.nlm.nih.gov/entrez/query.fcgi?CMD=search&DB=gene&term=ECK1959) | 1.563970 |
| [B2710](http://www.ncbi.nlm.nih.gov/entrez/query.fcgi?CMD=search&DB=gene&term=B2710) | [ECK2705](http://www.ncbi.nlm.nih.gov/entrez/query.fcgi?CMD=search&DB=gene&term=ECK2705) | 1.660091 |
| [napF](http://www.ncbi.nlm.nih.gov/entrez/query.fcgi?CMD=search&DB=gene&term=NAPF) | [ECK2200](http://www.ncbi.nlm.nih.gov/entrez/query.fcgi?CMD=search&DB=gene&term=ECK2200) | 1.591847 |
| [cspG](http://www.ncbi.nlm.nih.gov/entrez/query.fcgi?CMD=search&DB=gene&term=CSPG) | [ECK0980](http://www.ncbi.nlm.nih.gov/entrez/query.fcgi?CMD=search&DB=gene&term=ECK0980) | 1.671964 |
| [yahA](http://www.ncbi.nlm.nih.gov/entrez/query.fcgi?CMD=search&DB=gene&term=YAHA) | [ECK0313](http://www.ncbi.nlm.nih.gov/entrez/query.fcgi?CMD=search&DB=gene&term=ECK0313) | 2.101125 |
| [sfhB](http://www.ncbi.nlm.nih.gov/entrez/query.fcgi?CMD=search&DB=gene&term=SFHB) | [ECK2592](http://www.ncbi.nlm.nih.gov/entrez/query.fcgi?CMD=search&DB=gene&term=ECK2592) | 1.579754 |
| [yebK](http://www.ncbi.nlm.nih.gov/entrez/query.fcgi?CMD=search&DB=gene&term=YEBK) | [ECK1854](http://www.ncbi.nlm.nih.gov/entrez/query.fcgi?CMD=search&DB=gene&term=ECK1854) | 1.998762 |
| [kgtP](http://www.ncbi.nlm.nih.gov/entrez/query.fcgi?CMD=search&DB=gene&term=KGTP) | [ECK2585](http://www.ncbi.nlm.nih.gov/entrez/query.fcgi?CMD=search&DB=gene&term=ECK2585) | 2.358483 |
| [rpsH](http://www.ncbi.nlm.nih.gov/entrez/query.fcgi?CMD=search&DB=gene&term=RPSH) | [ECK3293](http://www.ncbi.nlm.nih.gov/entrez/query.fcgi?CMD=search&DB=gene&term=ECK3293) | 1.689817 |
| [mbhA](http://www.ncbi.nlm.nih.gov/entrez/query.fcgi?CMD=search&DB=gene&term=MBHA) | [ECK0231](http://www.ncbi.nlm.nih.gov/entrez/query.fcgi?CMD=search&DB=gene&term=ECK0231) | 1.711622 |
| [ugpQ](http://www.ncbi.nlm.nih.gov/entrez/query.fcgi?CMD=search&DB=gene&term=UGPQ) | [ECK3433](http://www.ncbi.nlm.nih.gov/entrez/query.fcgi?CMD=search&DB=gene&term=ECK3433) | 2.137842 |
| [yadS](http://www.ncbi.nlm.nih.gov/entrez/query.fcgi?CMD=search&DB=gene&term=YADS) | [ECK0156](http://www.ncbi.nlm.nih.gov/entrez/query.fcgi?CMD=search&DB=gene&term=ECK0156) | 2.017587 |
| [recO](http://www.ncbi.nlm.nih.gov/entrez/query.fcgi?CMD=search&DB=gene&term=RECO) | [ECK2563](http://www.ncbi.nlm.nih.gov/entrez/query.fcgi?CMD=search&DB=gene&term=ECK2563) | 1.527236 |
| [citG](http://www.ncbi.nlm.nih.gov/entrez/query.fcgi?CMD=search&DB=gene&term=CITG) | [ECK0606](http://www.ncbi.nlm.nih.gov/entrez/query.fcgi?CMD=search&DB=gene&term=ECK0606) | 1.623333 |
| [folA](http://www.ncbi.nlm.nih.gov/entrez/query.fcgi?CMD=search&DB=gene&term=FOLA) | [ECK0049](http://www.ncbi.nlm.nih.gov/entrez/query.fcgi?CMD=search&DB=gene&term=ECK0049) | 1.625219 |
| [cydC](http://www.ncbi.nlm.nih.gov/entrez/query.fcgi?CMD=search&DB=gene&term=CYDC) | [ECK0877](http://www.ncbi.nlm.nih.gov/entrez/query.fcgi?CMD=search&DB=gene&term=ECK0877) | 1.798349 |
| [yifB](http://www.ncbi.nlm.nih.gov/entrez/query.fcgi?CMD=search&DB=gene&term=YIFB) | [ECK3758](http://www.ncbi.nlm.nih.gov/entrez/query.fcgi?CMD=search&DB=gene&term=ECK3758) | 1.709593 |
| [moeB-R](http://www.ncbi.nlm.nih.gov/entrez/query.fcgi?CMD=search&DB=gene&term=MOEB-R) | [ECK0816](http://www.ncbi.nlm.nih.gov/entrez/query.fcgi?CMD=search&DB=gene&term=ECK0816) | 1.630849 |
| [mglC](http://www.ncbi.nlm.nih.gov/entrez/query.fcgi?CMD=search&DB=gene&term=MGLC) | [ECK2141](http://www.ncbi.nlm.nih.gov/entrez/query.fcgi?CMD=search&DB=gene&term=ECK2141) | 1.779120 |
| [cspC](http://www.ncbi.nlm.nih.gov/entrez/query.fcgi?CMD=search&DB=gene&term=CSPC) | [ECK1821](http://www.ncbi.nlm.nih.gov/entrez/query.fcgi?CMD=search&DB=gene&term=ECK1821) | 3.090209 |
| [atoA](http://www.ncbi.nlm.nih.gov/entrez/query.fcgi?CMD=search&DB=gene&term=ATOA) | [ECK2215](http://www.ncbi.nlm.nih.gov/entrez/query.fcgi?CMD=search&DB=gene&term=ECK2215) | 1.753427 |
| [ybbD](http://www.ncbi.nlm.nih.gov/entrez/query.fcgi?CMD=search&DB=gene&term=YBBD) | [ECK0494](http://www.ncbi.nlm.nih.gov/entrez/query.fcgi?CMD=search&DB=gene&term=ECK0494) | 2.585485 |
| [folK](http://www.ncbi.nlm.nih.gov/entrez/query.fcgi?CMD=search&DB=gene&term=FOLK) | [ECK0141](http://www.ncbi.nlm.nih.gov/entrez/query.fcgi?CMD=search&DB=gene&term=ECK0141) | 1.885711 |
| [glgS](http://www.ncbi.nlm.nih.gov/entrez/query.fcgi?CMD=search&DB=gene&term=GLGS) | [ECK3038](http://www.ncbi.nlm.nih.gov/entrez/query.fcgi?CMD=search&DB=gene&term=ECK3038) | 2.070471 |
| [cdd](http://www.ncbi.nlm.nih.gov/entrez/query.fcgi?CMD=search&DB=gene&term=CDD) | [ECK2136](http://www.ncbi.nlm.nih.gov/entrez/query.fcgi?CMD=search&DB=gene&term=ECK2136) | 1.656312 |
| [B2863](http://www.ncbi.nlm.nih.gov/entrez/query.fcgi?CMD=search&DB=gene&term=B2863) | [ECK2859](http://www.ncbi.nlm.nih.gov/entrez/query.fcgi?CMD=search&DB=gene&term=ECK2859) | 1.898705 |
| [yafN](http://www.ncbi.nlm.nih.gov/entrez/query.fcgi?CMD=search&DB=gene&term=YAFN) | [ECK0233](http://www.ncbi.nlm.nih.gov/entrez/query.fcgi?CMD=search&DB=gene&term=ECK0233) | 1.777507 |
| [B1578](http://www.ncbi.nlm.nih.gov/entrez/query.fcgi?CMD=search&DB=gene&term=B1578) | [ECK1572](http://www.ncbi.nlm.nih.gov/entrez/query.fcgi?CMD=search&DB=gene&term=ECK1572) | 1.647856 |
| [btuR](http://www.ncbi.nlm.nih.gov/entrez/query.fcgi?CMD=search&DB=gene&term=BTUR) | [ECK1264](http://www.ncbi.nlm.nih.gov/entrez/query.fcgi?CMD=search&DB=gene&term=ECK1264) | 1.565232 |
| [B2432](http://www.ncbi.nlm.nih.gov/entrez/query.fcgi?CMD=search&DB=gene&term=B2432) | [ECK2427](http://www.ncbi.nlm.nih.gov/entrez/query.fcgi?CMD=search&DB=gene&term=ECK2427) | 2.135634 |
| [yhcA](http://www.ncbi.nlm.nih.gov/entrez/query.fcgi?CMD=search&DB=gene&term=YHCA) | [ECK3205](http://www.ncbi.nlm.nih.gov/entrez/query.fcgi?CMD=search&DB=gene&term=ECK3205) | 2.504609 |
| [B1523](http://www.ncbi.nlm.nih.gov/entrez/query.fcgi?CMD=search&DB=gene&term=B1523) | [ECK1516](http://www.ncbi.nlm.nih.gov/entrez/query.fcgi?CMD=search&DB=gene&term=ECK1516) | 2.285775 |
| [B1815](http://www.ncbi.nlm.nih.gov/entrez/query.fcgi?CMD=search&DB=gene&term=B1815) | [ECK1813](http://www.ncbi.nlm.nih.gov/entrez/query.fcgi?CMD=search&DB=gene&term=ECK1813) | 1.765827 |
| [ybhS](http://www.ncbi.nlm.nih.gov/entrez/query.fcgi?CMD=search&DB=gene&term=YBHS) | [ECK0782](http://www.ncbi.nlm.nih.gov/entrez/query.fcgi?CMD=search&DB=gene&term=ECK0782) | 1.591548 |
| [nuoM](http://www.ncbi.nlm.nih.gov/entrez/query.fcgi?CMD=search&DB=gene&term=NUOM) | [ECK2271](http://www.ncbi.nlm.nih.gov/entrez/query.fcgi?CMD=search&DB=gene&term=ECK2271) | 1.996968 |
| [ytfI](http://www.ncbi.nlm.nih.gov/entrez/query.fcgi?CMD=search&DB=gene&term=YTFI) | [ECK4211](http://www.ncbi.nlm.nih.gov/entrez/query.fcgi?CMD=search&DB=gene&term=ECK4211) | 2.189650 |
| [yadQ](http://www.ncbi.nlm.nih.gov/entrez/query.fcgi?CMD=search&DB=gene&term=YADQ) | [ECK0154](http://www.ncbi.nlm.nih.gov/entrez/query.fcgi?CMD=search&DB=gene&term=ECK0154) | 1.530501 |
| [B1541](http://www.ncbi.nlm.nih.gov/entrez/query.fcgi?CMD=search&DB=gene&term=B1541) | [ECK1534](http://www.ncbi.nlm.nih.gov/entrez/query.fcgi?CMD=search&DB=gene&term=ECK1534) | 1.777446 |
| [mlc](http://www.ncbi.nlm.nih.gov/entrez/query.fcgi?CMD=search&DB=gene&term=MLC) | [ECK1589](http://www.ncbi.nlm.nih.gov/entrez/query.fcgi?CMD=search&DB=gene&term=ECK1589) | 1.647131 |
| [nuoC](http://www.ncbi.nlm.nih.gov/entrez/query.fcgi?CMD=search&DB=gene&term=NUOC) | [ECK2280](http://www.ncbi.nlm.nih.gov/entrez/query.fcgi?CMD=search&DB=gene&term=ECK2280) | 1.569045 |
| [cheY](http://www.ncbi.nlm.nih.gov/entrez/query.fcgi?CMD=search&DB=gene&term=CHEY) | [ECK1883](http://www.ncbi.nlm.nih.gov/entrez/query.fcgi?CMD=search&DB=gene&term=ECK1883) | 1.684099 |
| [B1199](http://www.ncbi.nlm.nih.gov/entrez/query.fcgi?CMD=search&DB=gene&term=B1199) | [ECK1187](http://www.ncbi.nlm.nih.gov/entrez/query.fcgi?CMD=search&DB=gene&term=ECK1187) | 1.870276 |
| [treR](http://www.ncbi.nlm.nih.gov/entrez/query.fcgi?CMD=search&DB=gene&term=TRER) | [ECK4236](http://www.ncbi.nlm.nih.gov/entrez/query.fcgi?CMD=search&DB=gene&term=ECK4236) | 1.904068 |
| [yaeG](http://www.ncbi.nlm.nih.gov/entrez/query.fcgi?CMD=search&DB=gene&term=YAEG) | [ECK0161](http://www.ncbi.nlm.nih.gov/entrez/query.fcgi?CMD=search&DB=gene&term=ECK0161) | 2.404050 |
| [B2866](http://www.ncbi.nlm.nih.gov/entrez/query.fcgi?CMD=search&DB=gene&term=B2866) | [ECK2862](http://www.ncbi.nlm.nih.gov/entrez/query.fcgi?CMD=search&DB=gene&term=ECK2862) | 1.691897 |
| [gcpE](http://www.ncbi.nlm.nih.gov/entrez/query.fcgi?CMD=search&DB=gene&term=GCPE) | [ECK2511](http://www.ncbi.nlm.nih.gov/entrez/query.fcgi?CMD=search&DB=gene&term=ECK2511) | 1.527332 |
| [B1446](http://www.ncbi.nlm.nih.gov/entrez/query.fcgi?CMD=search&DB=gene&term=B1446) | [ECK1440](http://www.ncbi.nlm.nih.gov/entrez/query.fcgi?CMD=search&DB=gene&term=ECK1440) | 2.254668 |
| [thiH](http://www.ncbi.nlm.nih.gov/entrez/query.fcgi?CMD=search&DB=gene&term=THIH) | [ECK3981](http://www.ncbi.nlm.nih.gov/entrez/query.fcgi?CMD=search&DB=gene&term=ECK3981) | 2.009915 |
| [cchA](http://www.ncbi.nlm.nih.gov/entrez/query.fcgi?CMD=search&DB=gene&term=CCHA) | [ECK2452](http://www.ncbi.nlm.nih.gov/entrez/query.fcgi?CMD=search&DB=gene&term=ECK2452) | 1.899654 |
| [B1964](http://www.ncbi.nlm.nih.gov/entrez/query.fcgi?CMD=search&DB=gene&term=B1964) | [ECK1962](http://www.ncbi.nlm.nih.gov/entrez/query.fcgi?CMD=search&DB=gene&term=ECK1962) | 2.155104 |
| [rbn](http://www.ncbi.nlm.nih.gov/entrez/query.fcgi?CMD=search&DB=gene&term=RBN) | [ECK3879](http://www.ncbi.nlm.nih.gov/entrez/query.fcgi?CMD=search&DB=gene&term=ECK3879) | 2.031235 |
| [leuB](http://www.ncbi.nlm.nih.gov/entrez/query.fcgi?CMD=search&DB=gene&term=LEUB) | [ECK0075](http://www.ncbi.nlm.nih.gov/entrez/query.fcgi?CMD=search&DB=gene&term=ECK0075) | 1.742631 |
| [fdrA](http://www.ncbi.nlm.nih.gov/entrez/query.fcgi?CMD=search&DB=gene&term=FDRA) | [ECK0511](http://www.ncbi.nlm.nih.gov/entrez/query.fcgi?CMD=search&DB=gene&term=ECK0511) | 1.696620 |
| [yojH](http://www.ncbi.nlm.nih.gov/entrez/query.fcgi?CMD=search&DB=gene&term=YOJH) | [ECK2202](http://www.ncbi.nlm.nih.gov/entrez/query.fcgi?CMD=search&DB=gene&term=ECK2202) | 2.048148 |
| [efp](http://www.ncbi.nlm.nih.gov/entrez/query.fcgi?CMD=search&DB=gene&term=EFP) | [ECK4141](http://www.ncbi.nlm.nih.gov/entrez/query.fcgi?CMD=search&DB=gene&term=ECK4141) | 1.535428 |
| [B0829](http://www.ncbi.nlm.nih.gov/entrez/query.fcgi?CMD=search&DB=gene&term=B0829) | [ECK0819](http://www.ncbi.nlm.nih.gov/entrez/query.fcgi?CMD=search&DB=gene&term=ECK0819) | 1.825797 |
| [glpQ](http://www.ncbi.nlm.nih.gov/entrez/query.fcgi?CMD=search&DB=gene&term=GLPQ) | [ECK2231](http://www.ncbi.nlm.nih.gov/entrez/query.fcgi?CMD=search&DB=gene&term=ECK2231) | 1.803944 |
| [hisS](http://www.ncbi.nlm.nih.gov/entrez/query.fcgi?CMD=search&DB=gene&term=HISS) | [ECK2510](http://www.ncbi.nlm.nih.gov/entrez/query.fcgi?CMD=search&DB=gene&term=ECK2510) | 1.585812 |
| [wecB](http://www.ncbi.nlm.nih.gov/entrez/query.fcgi?CMD=search&DB=gene&term=WECB) | [ECK3778](http://www.ncbi.nlm.nih.gov/entrez/query.fcgi?CMD=search&DB=gene&term=ECK3778) | 1.735980 |
| [yjfY](http://www.ncbi.nlm.nih.gov/entrez/query.fcgi?CMD=search&DB=gene&term=YJFY) | [ECK4195](http://www.ncbi.nlm.nih.gov/entrez/query.fcgi?CMD=search&DB=gene&term=ECK4195) | 2.010947 |
| [yafL](http://www.ncbi.nlm.nih.gov/entrez/query.fcgi?CMD=search&DB=gene&term=YAFL) | [ECK0228](http://www.ncbi.nlm.nih.gov/entrez/query.fcgi?CMD=search&DB=gene&term=ECK0228) | 1.935780 |
| [nadA](http://www.ncbi.nlm.nih.gov/entrez/query.fcgi?CMD=search&DB=gene&term=NADA) | [ECK0739](http://www.ncbi.nlm.nih.gov/entrez/query.fcgi?CMD=search&DB=gene&term=ECK0739) | 2.078081 |
| [chpS](http://www.ncbi.nlm.nih.gov/entrez/query.fcgi?CMD=search&DB=gene&term=CHPS) | [ECK4220](http://www.ncbi.nlm.nih.gov/entrez/query.fcgi?CMD=search&DB=gene&term=ECK4220) | 1.904554 |
| [flhB](http://www.ncbi.nlm.nih.gov/entrez/query.fcgi?CMD=search&DB=gene&term=FLHB) | [ECK1881](http://www.ncbi.nlm.nih.gov/entrez/query.fcgi?CMD=search&DB=gene&term=ECK1881) | 1.577767 |
| [ycdB](http://www.ncbi.nlm.nih.gov/entrez/query.fcgi?CMD=search&DB=gene&term=YCDB) | [ECK1009](http://www.ncbi.nlm.nih.gov/entrez/query.fcgi?CMD=search&DB=gene&term=ECK1009) | 1.651167 |
| [cobB](http://www.ncbi.nlm.nih.gov/entrez/query.fcgi?CMD=search&DB=gene&term=COBB) | [ECK1106](http://www.ncbi.nlm.nih.gov/entrez/query.fcgi?CMD=search&DB=gene&term=ECK1106) | 1.778642 |
| [B2433](http://www.ncbi.nlm.nih.gov/entrez/query.fcgi?CMD=search&DB=gene&term=B2433) | [ECK2428](http://www.ncbi.nlm.nih.gov/entrez/query.fcgi?CMD=search&DB=gene&term=ECK2428) | 1.692221 |
| [manX](http://www.ncbi.nlm.nih.gov/entrez/query.fcgi?CMD=search&DB=gene&term=MANX) | [ECK1815](http://www.ncbi.nlm.nih.gov/entrez/query.fcgi?CMD=search&DB=gene&term=ECK1815) | 1.691677 |
| [napB](http://www.ncbi.nlm.nih.gov/entrez/query.fcgi?CMD=search&DB=gene&term=NAPB) | [ECK2195](http://www.ncbi.nlm.nih.gov/entrez/query.fcgi?CMD=search&DB=gene&term=ECK2195) | 1.561832 |
| [yhaG](http://www.ncbi.nlm.nih.gov/entrez/query.fcgi?CMD=search&DB=gene&term=YHAG) | [ECK3116](http://www.ncbi.nlm.nih.gov/entrez/query.fcgi?CMD=search&DB=gene&term=ECK3116) | 1.763341 |
| [acrA](http://www.ncbi.nlm.nih.gov/entrez/query.fcgi?CMD=search&DB=gene&term=ACRA) | [ECK0457](http://www.ncbi.nlm.nih.gov/entrez/query.fcgi?CMD=search&DB=gene&term=ECK0457) | 1.821259 |
| [yihS](http://www.ncbi.nlm.nih.gov/entrez/query.fcgi?CMD=search&DB=gene&term=YIHS) | [ECK3873](http://www.ncbi.nlm.nih.gov/entrez/query.fcgi?CMD=search&DB=gene&term=ECK3873) | 1.726939 |
| [cysA](http://www.ncbi.nlm.nih.gov/entrez/query.fcgi?CMD=search&DB=gene&term=CYSA) | [ECK2417](http://www.ncbi.nlm.nih.gov/entrez/query.fcgi?CMD=search&DB=gene&term=ECK2417) | 1.948908 |
| [glgS-R](http://www.ncbi.nlm.nih.gov/entrez/query.fcgi?CMD=search&DB=gene&term=GLGS-R) | [ECK3038](http://www.ncbi.nlm.nih.gov/entrez/query.fcgi?CMD=search&DB=gene&term=ECK3038) | 2.157159 |
| [ruvA](http://www.ncbi.nlm.nih.gov/entrez/query.fcgi?CMD=search&DB=gene&term=RUVA) | [ECK1862](http://www.ncbi.nlm.nih.gov/entrez/query.fcgi?CMD=search&DB=gene&term=ECK1862) | 1.652759 |
| [ykfE](http://www.ncbi.nlm.nih.gov/entrez/query.fcgi?CMD=search&DB=gene&term=YKFE) | [ECK0221](http://www.ncbi.nlm.nih.gov/entrez/query.fcgi?CMD=search&DB=gene&term=ECK0221) | 1.534722 |
| mdaB | ECK3019 | 1.910507 |
| [yigP](http://www.ncbi.nlm.nih.gov/entrez/query.fcgi?CMD=search&DB=gene&term=YIGP) | [ECK3828](http://www.ncbi.nlm.nih.gov/entrez/query.fcgi?CMD=search&DB=gene&term=ECK3828) | 1.591076 |
| [yheN](http://www.ncbi.nlm.nih.gov/entrez/query.fcgi?CMD=search&DB=gene&term=YHEN) | [ECK3332](http://www.ncbi.nlm.nih.gov/entrez/query.fcgi?CMD=search&DB=gene&term=ECK3332) | 1.823913 |
| [ybaB](http://www.ncbi.nlm.nih.gov/entrez/query.fcgi?CMD=search&DB=gene&term=YBAB) | [ECK0465](http://www.ncbi.nlm.nih.gov/entrez/query.fcgi?CMD=search&DB=gene&term=ECK0465) | 1.532688 |
| [yfcG](http://www.ncbi.nlm.nih.gov/entrez/query.fcgi?CMD=search&DB=gene&term=YFCG) | [ECK2296](http://www.ncbi.nlm.nih.gov/entrez/query.fcgi?CMD=search&DB=gene&term=ECK2296) | 1.595351 |
| [ydcA](http://www.ncbi.nlm.nih.gov/entrez/query.fcgi?CMD=search&DB=gene&term=YDCA) | [ECK1411](http://www.ncbi.nlm.nih.gov/entrez/query.fcgi?CMD=search&DB=gene&term=ECK1411) | 1.559146 |
| [dsbA](http://www.ncbi.nlm.nih.gov/entrez/query.fcgi?CMD=search&DB=gene&term=DSBA) | [ECK3852](http://www.ncbi.nlm.nih.gov/entrez/query.fcgi?CMD=search&DB=gene&term=ECK3852) | 2.094439 |
| [rcsC](http://www.ncbi.nlm.nih.gov/entrez/query.fcgi?CMD=search&DB=gene&term=RCSC) | [ECK2211](http://www.ncbi.nlm.nih.gov/entrez/query.fcgi?CMD=search&DB=gene&term=ECK2211) | 1.574088 |
| [yjiT](http://www.ncbi.nlm.nih.gov/entrez/query.fcgi?CMD=search&DB=gene&term=YJIT) | [ECK4333](http://www.ncbi.nlm.nih.gov/entrez/query.fcgi?CMD=search&DB=gene&term=ECK4333) | 1.562214 |
| [ycgE](http://www.ncbi.nlm.nih.gov/entrez/query.fcgi?CMD=search&DB=gene&term=YCGE) | [ECK1149](http://www.ncbi.nlm.nih.gov/entrez/query.fcgi?CMD=search&DB=gene&term=ECK1149) | 1.914174 |
| [yjjQ](http://www.ncbi.nlm.nih.gov/entrez/query.fcgi?CMD=search&DB=gene&term=YJJQ) | [ECK4355](http://www.ncbi.nlm.nih.gov/entrez/query.fcgi?CMD=search&DB=gene&term=ECK4355) | 1.538125 |
| [yehQ](http://www.ncbi.nlm.nih.gov/entrez/query.fcgi?CMD=search&DB=gene&term=YEHQ) | [ECK2114](http://www.ncbi.nlm.nih.gov/entrez/query.fcgi?CMD=search&DB=gene&term=ECK2114) | 1.820804 |
| [lysS](http://www.ncbi.nlm.nih.gov/entrez/query.fcgi?CMD=search&DB=gene&term=LYSS) | [ECK2885](http://www.ncbi.nlm.nih.gov/entrez/query.fcgi?CMD=search&DB=gene&term=ECK2885) | 1.540937 |
| [gatC](http://www.ncbi.nlm.nih.gov/entrez/query.fcgi?CMD=search&DB=gene&term=GATC) | [ECK2085](http://www.ncbi.nlm.nih.gov/entrez/query.fcgi?CMD=search&DB=gene&term=ECK2085) | 2.683008 |
| [rplJ](http://www.ncbi.nlm.nih.gov/entrez/query.fcgi?CMD=search&DB=gene&term=RPLJ) | [ECK3976](http://www.ncbi.nlm.nih.gov/entrez/query.fcgi?CMD=search&DB=gene&term=ECK3976) | 1.735385 |
| [B1953](http://www.ncbi.nlm.nih.gov/entrez/query.fcgi?CMD=search&DB=gene&term=B1953) | [ECK1951](http://www.ncbi.nlm.nih.gov/entrez/query.fcgi?CMD=search&DB=gene&term=ECK1951) | 1.600454 |
| [flgK](http://www.ncbi.nlm.nih.gov/entrez/query.fcgi?CMD=search&DB=gene&term=FLGK) | [ECK1067](http://www.ncbi.nlm.nih.gov/entrez/query.fcgi?CMD=search&DB=gene&term=ECK1067) | 1.808925 |
| [ybbC](http://www.ncbi.nlm.nih.gov/entrez/query.fcgi?CMD=search&DB=gene&term=YBBC) | [ECK0492](http://www.ncbi.nlm.nih.gov/entrez/query.fcgi?CMD=search&DB=gene&term=ECK0492) | 1.545532 |
| [yheI](http://www.ncbi.nlm.nih.gov/entrez/query.fcgi?CMD=search&DB=gene&term=YHEI) | [ECK3318](http://www.ncbi.nlm.nih.gov/entrez/query.fcgi?CMD=search&DB=gene&term=ECK3318) | 1.579565 |
| [rtn](http://www.ncbi.nlm.nih.gov/entrez/query.fcgi?CMD=search&DB=gene&term=RTN) | [ECK2170](http://www.ncbi.nlm.nih.gov/entrez/query.fcgi?CMD=search&DB=gene&term=ECK2170) | 2.091281 |
| [yjiA](http://www.ncbi.nlm.nih.gov/entrez/query.fcgi?CMD=search&DB=gene&term=YJIA) | [ECK4342](http://www.ncbi.nlm.nih.gov/entrez/query.fcgi?CMD=search&DB=gene&term=ECK4342) | 1.667327 |
| [yrbG](http://www.ncbi.nlm.nih.gov/entrez/query.fcgi?CMD=search&DB=gene&term=YRBG) | [ECK3185](http://www.ncbi.nlm.nih.gov/entrez/query.fcgi?CMD=search&DB=gene&term=ECK3185) | 1.698980 |
| [yfjN](http://www.ncbi.nlm.nih.gov/entrez/query.fcgi?CMD=search&DB=gene&term=YFJN) | [ECK2626](http://www.ncbi.nlm.nih.gov/entrez/query.fcgi?CMD=search&DB=gene&term=ECK2626) | 2.653341 |
| [yffB](http://www.ncbi.nlm.nih.gov/entrez/query.fcgi?CMD=search&DB=gene&term=YFFB) | [ECK2466](http://www.ncbi.nlm.nih.gov/entrez/query.fcgi?CMD=search&DB=gene&term=ECK2466) | 1.586001 |
| [uxaB](http://www.ncbi.nlm.nih.gov/entrez/query.fcgi?CMD=search&DB=gene&term=UXAB) | [ECK1514](http://www.ncbi.nlm.nih.gov/entrez/query.fcgi?CMD=search&DB=gene&term=ECK1514) | 1.587168 |
| [B2461](http://www.ncbi.nlm.nih.gov/entrez/query.fcgi?CMD=search&DB=gene&term=B2461) | [ECK2456](http://www.ncbi.nlm.nih.gov/entrez/query.fcgi?CMD=search&DB=gene&term=ECK2456) | 1.735235 |
| [dsbE](http://www.ncbi.nlm.nih.gov/entrez/query.fcgi?CMD=search&DB=gene&term=DSBE) | [ECK2187](http://www.ncbi.nlm.nih.gov/entrez/query.fcgi?CMD=search&DB=gene&term=ECK2187) | 1.882562 |
| [yecH](http://www.ncbi.nlm.nih.gov/entrez/query.fcgi?CMD=search&DB=gene&term=YECH) | [ECK1905](http://www.ncbi.nlm.nih.gov/entrez/query.fcgi?CMD=search&DB=gene&term=ECK1905) | 1.516529 |
| [B3051](http://www.ncbi.nlm.nih.gov/entrez/query.fcgi?CMD=search&DB=gene&term=B3051) | [ECK3040](http://www.ncbi.nlm.nih.gov/entrez/query.fcgi?CMD=search&DB=gene&term=ECK3040) | 1.698791 |
| [B2434](http://www.ncbi.nlm.nih.gov/entrez/query.fcgi?CMD=search&DB=gene&term=B2434) | [ECK2429](http://www.ncbi.nlm.nih.gov/entrez/query.fcgi?CMD=search&DB=gene&term=ECK2429) | 2.082000 |
| [flhE](http://www.ncbi.nlm.nih.gov/entrez/query.fcgi?CMD=search&DB=gene&term=FLHE) | [ECK1879](http://www.ncbi.nlm.nih.gov/entrez/query.fcgi?CMD=search&DB=gene&term=ECK1879) | 1.620626 |
| [surE](http://www.ncbi.nlm.nih.gov/entrez/query.fcgi?CMD=search&DB=gene&term=SURE) | [ECK2739](http://www.ncbi.nlm.nih.gov/entrez/query.fcgi?CMD=search&DB=gene&term=ECK2739) | 1.550016 |
| [yceB](http://www.ncbi.nlm.nih.gov/entrez/query.fcgi?CMD=search&DB=gene&term=YCEB) | [ECK1048](http://www.ncbi.nlm.nih.gov/entrez/query.fcgi?CMD=search&DB=gene&term=ECK1048) | 1.680981 |
| [cheB](http://www.ncbi.nlm.nih.gov/entrez/query.fcgi?CMD=search&DB=gene&term=CHEB) | [ECK1884](http://www.ncbi.nlm.nih.gov/entrez/query.fcgi?CMD=search&DB=gene&term=ECK1884) | 1.512111 |
| [tehA](http://www.ncbi.nlm.nih.gov/entrez/query.fcgi?CMD=search&DB=gene&term=TEHA) | [ECK1422](http://www.ncbi.nlm.nih.gov/entrez/query.fcgi?CMD=search&DB=gene&term=ECK1422) | 2.200961 |
| [nuoG](http://www.ncbi.nlm.nih.gov/entrez/query.fcgi?CMD=search&DB=gene&term=NUOG) | [ECK2277](http://www.ncbi.nlm.nih.gov/entrez/query.fcgi?CMD=search&DB=gene&term=ECK2277) | 2.584888 |
| [ypfI](http://www.ncbi.nlm.nih.gov/entrez/query.fcgi?CMD=search&DB=gene&term=YPFI) | [ECK2470](http://www.ncbi.nlm.nih.gov/entrez/query.fcgi?CMD=search&DB=gene&term=ECK2470) | 2.305040 |
| [folC](http://www.ncbi.nlm.nih.gov/entrez/query.fcgi?CMD=search&DB=gene&term=FOLC) | [ECK2309](http://www.ncbi.nlm.nih.gov/entrez/query.fcgi?CMD=search&DB=gene&term=ECK2309) | 1.503756 |
| [ycfN](http://www.ncbi.nlm.nih.gov/entrez/query.fcgi?CMD=search&DB=gene&term=YCFN) | [ECK1092](http://www.ncbi.nlm.nih.gov/entrez/query.fcgi?CMD=search&DB=gene&term=ECK1092) | 2.160299 |
| [recE](http://www.ncbi.nlm.nih.gov/entrez/query.fcgi?CMD=search&DB=gene&term=RECE) | [ECK1347](http://www.ncbi.nlm.nih.gov/entrez/query.fcgi?CMD=search&DB=gene&term=ECK1347) | 1.646131 |
| [rpiA](http://www.ncbi.nlm.nih.gov/entrez/query.fcgi?CMD=search&DB=gene&term=RPIA) | [ECK2910](http://www.ncbi.nlm.nih.gov/entrez/query.fcgi?CMD=search&DB=gene&term=ECK2910) | 1.537576 |
| [yciH](http://www.ncbi.nlm.nih.gov/entrez/query.fcgi?CMD=search&DB=gene&term=YCIH) | [ECK1277](http://www.ncbi.nlm.nih.gov/entrez/query.fcgi?CMD=search&DB=gene&term=ECK1277) | 1.812484 |
| [B4140](http://www.ncbi.nlm.nih.gov/entrez/query.fcgi?CMD=search&DB=gene&term=B4140) | [ECK4134](http://www.ncbi.nlm.nih.gov/entrez/query.fcgi?CMD=search&DB=gene&term=ECK4134) | 2.019278 |
| [yciM](http://www.ncbi.nlm.nih.gov/entrez/query.fcgi?CMD=search&DB=gene&term=YCIM) | [ECK1275](http://www.ncbi.nlm.nih.gov/entrez/query.fcgi?CMD=search&DB=gene&term=ECK1275) | 1.895176 |
| [nrfF](http://www.ncbi.nlm.nih.gov/entrez/query.fcgi?CMD=search&DB=gene&term=NRFF) | [ECK4068](http://www.ncbi.nlm.nih.gov/entrez/query.fcgi?CMD=search&DB=gene&term=ECK4068) | 1.513575 |
| [yijF](http://www.ncbi.nlm.nih.gov/entrez/query.fcgi?CMD=search&DB=gene&term=YIJF) | [ECK3936](http://www.ncbi.nlm.nih.gov/entrez/query.fcgi?CMD=search&DB=gene&term=ECK3936) | 1.526122 |
| [ycaR](http://www.ncbi.nlm.nih.gov/entrez/query.fcgi?CMD=search&DB=gene&term=YCAR) | [ECK0908](http://www.ncbi.nlm.nih.gov/entrez/query.fcgi?CMD=search&DB=gene&term=ECK0908) | 1.927507 |
| yacC | ECK0121 | -1.769732 |
| dinD | ECK3635 | -1.628780 |
| menB | ECK2256 | -1.865654 |
| yhdA | ECK3240 | -1.818724 |
| yabI | ECK0066 | -2.000806 |
| nirD | ECK3354 | -1.705925 |
| ychJ | ECK1228 | -1.927514 |
| ompT | ECK0557 | -1.909177 |
| yjiM | ECK4326 | -2.574085 |
| yojL | ECK2206 | -1.764357 |
| B1471 | ECK1465 | -1.587824 |
| dacA | ECK0625 | -2.225531 |
| hslU | ECK3923 | -2.603575 |
| rpsP | ECK2606 | -1.541972 |
| sdhC | ECK0710 | -5.226546 |
| hha | ECK0454 | -1.782909 |
| ydjX | ECK1748 | -1.563755 |
| ylaB | ECK0451 | -2.008151 |
| tpx | ECK1320 | -2.503449 |
| cutC | ECK1875 | -2.060213 |
| yhcJ | ECK3212 | -2.347925 |
| hslV | ECK3924 | -3.204673 |
| sucB | ECK0715 | -3.723353 |
| perR | ECK0256 | -1.973553 |
| polB | ECK0061 | -5.121324 |
| yfbB | ECK2257 | -1.601426 |
| cmk | ECK0901 | -2.079595 |
| dsbC | ECK2888 | -1.592541 |
| lldP | ECK3593 | -2.417374 |
| B1806 | ECK1804 | -2.511358 |
| insB_2 | ECK0022 | -1.692962 |
| B1811 | ECK1809 | -5.784205 |
| yagN | ECK0279 | -1.538489 |
| yaeR | ECK0186 | -3.111188 |
| gltA | ECK0709 | -5.478395 |
| wcaF | ECK2048 | -1.814102 |
| yccA | ECK0961 | -2.427025 |
| hfq | ECK4168 | -1.890491 |
| yejF | ECK2174 | -1.864381 |
| menC | ECK2255 | -1.557282 |
| ybeV | ECK0642 | -2.356392 |
| ybbT | ECK0498 | -2.145575 |
| B1551 | ECK1545 | -1.815696 |
| aceE | ECK0113 | -1.791980 |
| B1976 | ECK1972 | -2.009649 |
| intF | ECK0280 | -1.744538 |
| yjcT | ECK4077 | -1.827705 |
| yegX | ECK2095 | -1.862655 |
| ybhC | ECK0761 | -1.903317 |
| B1012 | ECK1003 | -2.038887 |
| sbp | ECK3909 | -1.595263 |
| aceF | ECK0114 | -2.457307 |
| rseC | ECK2568 | -1.544031 |
| acpP | ECK1080 | -2.381710 |
| yiaH | ECK3550 | -1.675229 |
| ugD | ECK2023 | -1.520145 |
| pspE | ECK1303 | -2.769456 |
| acnB | ECK0117 | -2.204507 |
| rplS | ECK2603 | -1.555328 |
| ybgK | ECK0701 | -1.547019 |
| fhlA | ECK2726 | -2.012804 |
| psiF | ECK0379 | -2.383446 |
| ycaL | ECK0900 | -1.599876 |
| cspB | ECK1551 | -1.891016 |
| yfjP | ECK2628 | -1.585874 |
| cspI | ECK1546 | -5.425934 |
| ycaI | ECK0904 | -2.035101 |
| tdcD | ECK3104 | -1.678717 |
| livH | ECK3441 | -1.662634 |
| ybcZ | ECK0562 | -1.534391 |
| yieM | ECK3739 | -1.856223 |
| B1228 | ECK1222 | -1.669466 |
| sspA | ECK3218 | -1.814888 |
| yhaR | ECK3102 | -2.195356 |
| atpC | ECK3724 | -1.899664 |
| tolA | ECK0728 | -1.954911 |
| ykgJ | ECK0287 | -1.561115 |
| rpmB | ECK3627 | -1.935482 |
| ybfM | ECK0669 | -1.942438 |
| fur | ECK0671 | -1.903090 |
| B2740 | ECK2735 | -2.679119 |
| rpsG | ECK3328 | -3.202846 |
| yagH | ECK0272 | -1.584210 |
| rbsB | ECK3745 | -2.576408 |
| pntA | ECK1598 | -1.675686 |
| holA | ECK0633 | -1.625861 |
| suRA | ECK0054 | -1.855223 |
| B1995 | ECK1989 | -1.695460 |
| pyrD | ECK0936 | -2.525532 |
| bglX | ECK2125 | -1.510998 |
| B2710 | ECK2705 | -1.651181 |
| narH | ECK1219 | -1.552349 |
| trpD | ECK1257 | -2.197351 |
| B2618 | ECK2614 | -1.954814 |
| yceE | ECK1038 | -1.613031 |
| lpxB | ECK0181 | -1.612803 |
| ytfF | ECK4206 | -1.535993 |
| yhfQ | ECK3361 | -1.854675 |
| osmC | ECK1476 | -2.797395 |
| napF | ECK2200 | -1.582126 |
| yqaB | ECK2685 | -3.760714 |
| yahA | ECK0313 | -2.103850 |
| ymfA | ECK1108 | -1.787585 |
| yejO | ECK2184 | -2.097790 |
| phoH | ECK1010 | -1.665410 |
| yheK | ECK3320 | -1.736329 |
| ycbR | ECK0930 | -2.207193 |
| menE | ECK2254 | -1.639720 |
| trpC-R | ECK1256 | -1.579735 |
| cydC | ECK0877 | -2.649563 |
| ylbF | ECK0513 | -1.824224 |
| ackA | ECK2290 | -2.033190 |
| hyfC | ECK2479 | -1.665821 |
| ygdE | ECK2801 | -1.785376 |
| yebK | ECK1854 | -1.724056 |
| B1007 | ECK0998 | -1.735899 |
| yceL | ECK1050 | -2.057021 |
| kgtP | ECK2585 | -2.331114 |
| nirB | ECK3353 | -1.733382 |
| yphC | ECK2542 | -1.754923 |
| ilvB | ECK3662 | -2.104565 |
| yacH | ECK0116 | -1.531941 |
| cspC | ECK1821 | -2.983206 |
| tauC | ECK0364 | -1.587746 |
| ygeA | ECK2838 | -1.716369 |
| yieP | ECK3749 | -2.303410 |
| B2432 | ECK2427 | -1.678068 |
| ybfG | ECK0678 | -1.935529 |
| B2834 | ECK2830 | -1.549706 |
| B2863 | ECK2859 | -1.864610 |
| B2998 | ECK2992 | -1.568288 |
| yeaL | ECK1787 | -1.747971 |
| holE | ECK1843 | -1.537457 |
| glgS | ECK3038 | -2.813802 |
| yiaD | ECK3539 | -1.616667 |
| gloB | ECK0212 | -1.575785 |
| yggC | ECK2924 | -1.712882 |
| B1541 | ECK1534 | -1.804288 |
| yaeG | ECK0161 | -2.459861 |
| treR | ECK4236 | -1.608128 |
| prmA | ECK3246 | -2.345368 |
| cdd | ECK2136 | -1.829296 |
| thdF | ECK3699 | -1.932046 |
| B1604 | ECK1599 | -1.520281 |
| pldB | ECK3819 | -1.663057 |
| yjcR | ECK4075 | -2.058018 |
| cspA | ECK3543 | -2.167342 |
| cchA | ECK2452 | -1.866226 |
| B1983 | ECK1978 | -1.791606 |
| leuB | ECK0075 | -2.080812 |
| nadA | ECK0739 | -2.043091 |
| hisS | ECK2510 | -1.973683 |
| rpsK | ECK3284 | -2.377029 |
| wecB | ECK3778 | -2.432338 |
| ygaD | ECK2695 | -1.825193 |
| ygiX | ECK3016 | -2.296333 |
| phnG | ECK4094 | -1.630202 |
| soxS | ECK4054 | -1.789086 |
| fdrA | ECK0511 | -1.539921 |
| mlc | ECK1589 | -1.816148 |
| ydaT | ECK1356 | -1.825636 |
| B1964 | ECK1962 | -1.524573 |
| yihS | ECK3873 | -1.590668 |
| sppA | ECK1764 | -1.622147 |
| ybgG | ECK0720 | -1.656728 |
| yfhC | ECK2557 | -1.719980 |
| B1314 | ECK1309 | -1.777041 |
| B2760 | ECK2755 | -2.111679 |
| ygcU | ECK2767 | -2.324184 |
| yihA | ECK3857 | -1.586299 |
| ftsY | ECK3448 | -2.485607 |
| potG | ECK0846 | -1.508964 |
| napB | ECK2195 | -2.176974 |
| yniC | ECK1725 | -1.516477 |
| bfr | ECK3323 | -1.624323 |
| B1399 | ECK1396 | -1.983728 |
| yhaJ | ECK3096 | -1.804132 |
| yciL | ECK1263 | -1.516069 |
| mrsA | ECK3165 | -2.807452 |
| rplQ | ECK3281 | -2.228854 |
| yeaN | ECK1789 | -1.986137 |
| ruvA | ECK1862 | -1.736063 |
| wrbA | ECK0995 | -1.597290 |
| serS | ECK0884 | -1.934435 |
| ychN | ECK1213 | -1.565597 |
| yeiH | ECK2151 | -1.740466 |
| smg | ECK3271 | -1.829372 |
| ycjU | ECK1312 | -2.119297 |
| ddlB | ECK0093 | -1.700188 |
| B3776 | ECK3768 | -1.569111 |
| cvpA | ECK2307 | -2.532928 |
| mdaB | ECK3019 | -2.071357 |
| B0298 | ECK0298 | -1.558773 |
| ydhD | ECK1650 | -1.764876 |
| pdxA | ECK0053 | -1.751146 |
| pth | ECK1192 | -2.133195 |
| ycfP | ECK1094 | -3.231233 |
| yhhL | ECK3450 | -1.815767 |
| yfeG | ECK2432 | -1.554868 |
| racC | ECK1348 | -1.857479 |
| pepP | ECK2903 | -2.010652 |
| ytfS | ECK4224 | -2.219486 |
| rplJ | ECK3976 | -2.365663 |
| hyfI | ECK2485 | -1.714988 |
| ykfE | ECK0221 | -2.715482 |
| rpiB | ECK4083 | -2.108956 |
| rnd | ECK1802 | -1.599697 |
| fliT | ECK1925 | -2.012987 |
| B1953 | ECK1951 | -1.718499 |
| artM | ECK0852 | -1.775991 |
| yjiT | ECK4333 | -1.613536 |
| ykfG | ECK0249 | -1.673386 |
| mhpF | ECK0348 | -2.558617 |
| selD | ECK1762 | -2.007988 |
| yjjQ | ECK4355 | -1.505213 |
| ycfT | ECK1101 | -1.774087 |
| yfjN | ECK2626 | -2.268581 |
| motA | ECK1891 | -1.847183 |
| yeaM | ECK1788 | -1.993724 |
| B1371 | ECK1366 | -1.738041 |
| B3051 | ECK3040 | -3.087694 |
| yrbG | ECK3185 | -1.514688 |
| B2434 | ECK2429 | -2.716858 |
| yliG | ECK0825 | -1.547399 |
| pitA | ECK3478 | -1.773331 |
| yedJ | ECK1960 | -2.190290 |
| tehA | ECK1422 | -2.261668 |
| hisB | ECK2017 | -1.619531 |
| fumB | ECK4115 | -1.667381 |
| metL | ECK3932 | -2.264326 |
| uidR | ECK1613 | -1.529491 |
| B1963 | ECK1961 | -1.720596 |
| wzxC | ECK2040 | -2.288229 |
| yceB | ECK1048 | -1.633468 |
| ycfU | ECK1102 | -1.734345 |
| arcB | ECK3200 | -1.702296 |
| nuoG | ECK2277 | -2.440615 |
| aceK | ECK4008 | -2.006906 |
| gyrA | ECK2223 | -1.700214 |
| yhjG | ECK3509 | -1.518522 |
| dcp | ECK1531 | -1.818165 |
| yihV | ECK3876 | -1.709450 |
| cobU | ECK1988 | -1.598561 |
| yciM | ECK1275 | -1.773122 |
