# Supplementary material for: Datasets for transcriptomics, q-proteomics and phenotype microarrays of polyphosphate metabolism mutants from Escherichia coli
Source: Data Brief. 2017 Mar 18;12:13–7. doi: 10.1016/j.dib.2017.03.010 (PMC5367803; doi:10.1016/j.dib.2017.03.010)
Supplement: Supplementary file 2 — Supplementary material [file mmc2.zip › BBAGEN-16-480/Ecoli polyP_versus_EcoliK12.doc]

```
EcolSIDx5935xppkx/ppxx versus EcolSIDx5932xK12


BiOLOG
21124 Cabot Blvd.
Hayward, CA 94545


                    Phenotype MicroArray Analysis
              Test: Ecol_SID_5935_ppk-/ppx-
         Reference: Ecol_SID_5932_K12
       Report Date: 6/16/2010 11:20:04 AM


Reproducibiltiy Analysis:

Test                                               Reference
PM01:    00: passes                      PM01:    01: passes
PM02:    00: passes                      PM02:    00: passes
PM03:    00: passes                      PM03:    01: passes
PM04:    04: passes                      PM04:    00: passes
PM05:    00: passes                      PM05:    00: passes
PM06:    00: passes                      PM06:    00: passes
PM07:    00: passes                      PM07:    00: passes
PM08:    00: passes                      PM08:    00: passes
PM09:    00: passes                      PM09:    00: passes
PM10:    00: passes                      PM10:    00: passes
PM11:    00: passes                      PM11:    00: passes
PM12:    03: passes                      PM12:    01: passes
PM13:    00: passes                      PM13:    00: passes
PM14:    00: passes                      PM14:    00: passes
PM15:    00: passes                      PM15:    00: passes
PM16:    00: passes                      PM16:    00: passes
PM17:    05: passes                      PM17:    02: passes
PM18:    02: passes                      PM18:    00: passes
PM19:    03: passes                      PM19:    00: passes
PM20:    00: passes                      PM20:    00: passes
PM21:    -1:    N/A                      PM21:    -1:    N/A
PM22:    -1:    N/A                      PM22:    -1:    N/A
PM23:    -1:    N/A                      PM23:    -1:    N/A
PM24:    -1:    N/A                      PM24:    -1:    N/A
PM25:    -1:    N/A                      PM25:    -1:    N/A
PM26:    -1:    N/A                      PM26:    -1:    N/A
PM27:    -1:    N/A                      PM27:    -1:    N/A
PM28:    -1:    N/A                      PM28:    -1:    N/A
PM29:    -1:    N/A                      PM29:    -1:    N/A
PM30:    -1:    N/A                      PM30:    -1:    N/A
PM31:    -1:    N/A                      PM31:    -1:    N/A
PM32:    -1:    N/A                      PM32:    -1:    N/A
PM33:    -1:    N/A                      PM33:    -1:    N/A
PM34:    -1:    N/A                      PM34:    -1:    N/A
PM35:    -1:    N/A                      PM35:    -1:    N/A
PM36:    -1:    N/A                      PM36:    -1:    N/A
PM37:    -1:    N/A                      PM37:    -1:    N/A
PM38:    -1:    N/A                      PM38:    -1:    N/A
PM39:    -1:    N/A                      PM39:    -1:    N/A
PM40:    -1:    N/A                      PM40:    -1:    N/A


Replicate 1 versus Replicate 2 of Test:
Ecol_SID_5935_ppk-/ppx-
```

Replicate 1 versus Replicate 2 of Reference:

Ecol_SID_5932_K12

```
Run 1:
Ecol_SID_5935_ppk-/ppx-( green )
 versus
Ecol_SID_5932_K12( red )
```

```
Run 2:
Ecol_SID_5935_ppk-/ppx-( green )
 versus
Ecol_SID_5932_K12( red )
```

```
Consensus:
Ecol_SID_5935_ppk-/ppx-( green )
 versus
Ecol_SID_5932_K12( red )
```

```
PM Report:
Ecol_SID_5935_ppk-/ppx-_
 versus
Ecol_SID_5932_K12_

Phenotypes Gained:
PM10 	H09	 102	X-?-D-Galactosaminide	?-D-galactosaminidase
PM10 	H04	 110	X-?-D-Galactoside	?-D-galactosidase
PM10 	H08	 111	X-?-D-Glucosaminide	?-D-glucosaminidase
PM10 	H02	 145	X-?-D-Glucoside	?-D-glucosidase
PM10 	H03	 103	X-?-D-Glucoside	?-D-glucosidase
PM10 	H06	 105	X-?-D-Glucuronide	?-D-glucuronidase
PM10 	H07	 104	X-?-D-Glucuronide	?-D-glucuronidase
PM10 	H10	  99	X-?-D-Mannoside	?-D-mannosidase
PM10 	H11	  93	X-PO4	aryl phosphatase
PM01 	G11	 120	D-Malic Acid	C-Source, carboxylic acid
PM01 	E07	  97	a-Hydroxybutyric Acid	C-Source, carboxylic acid
PM01 	D07	  89	a-Ketobutyric Acid	C-Source, carboxylic acid
PM01 	F07	  82	Propionic Acid	C-Source, carboxylic acid
PM01 	G09	  76	Mono-Methylsuccinate	C-Source, carboxylic acid
PM10 	H01	  91	X-Caprylate	caprylate esterase
PM15B	A05,A06,A07	 226	Guanidine hydrochloride	membrane, chaotropic agent
PM19 	B03	  43	Methyltrioctylammonium chloride	membrane, detergent, cationic
PM03B	B02	 144	Glycine	N-Source, amino acid
PM03B	C12	 114	L-Ornithine	N-Source, amino acid
PM03B	C04	 111	D-Asparagine	N-Source, amino acid
PM03B	A07	 108	L-Alanine	N-Source, amino acid
PM03B	A09	 107	L-Asparagine	N-Source, amino acid
PM03B	A08	 105	L-Arginine	N-Source, amino acid
PM03B	B06	  89	L-Lysine	N-Source, amino acid
PM03B	B01	  85	L-Glutamine	N-Source, amino acid
PM08 	A02	  80	L-Glutamine	N-Source, amino acid
PM03B	B08	  79	L-Phenylalanine	N-Source, amino acid
PM03B	C03	  77	D-Alanine	N-Source, amino acid
PM03B	A12	  76	L-Glutamic Acid	N-Source, amino acid
PM03B	C09	  73	D-Valine	N-Source, amino acid
PM03B	A02	 120	Ammonia	N-Source, inorganic
PM03B	G11	 109	d-Amino-N-Valeric Acid	N-Source, other
PM03B	F05	 102	Cytosine	N-Source, other
PM03B	D11	  85	Putrescine	N-Source, other
PM03B	F02	  85	Adenine	N-Source, other
PM03B	G08	  75	g-Amino-N-Butyric Acid	N-Source, other
PM08 	C07	 142	Gln-Glu	N-Source, peptide
PM03B	H11	 139	Gly-Met	N-Source, peptide
PM07 	B02	 135	Lys-Ser	N-Source, peptide
PM08 	A10	 133	Asp-Gly	N-Source, peptide
PM07 	A06	 132	Lys-Ala	N-Source, peptide
PM08 	A12	 130	Gly-Asn	N-Source, peptide
PM08 	B12	 130	Lys-Asp	N-Source, peptide
PM06 	C12	 128	Asp-Lys	N-Source, peptide
PM06 	F11	 126	His-Ser	N-Source, peptide
PM06 	C11	 124	Asp-Leu	N-Source, peptide
PM08 	A08	 124	Asp-Ala	N-Source, peptide
PM08 	A09	 123	Asp-Gln	N-Source, peptide
PM06 	H07	 121	Leu-Glu	N-Source, peptide
PM07 	E05	 121	Ser-Met	N-Source, peptide
PM07 	D05	 120	Pro-Asp	N-Source, peptide
PM08 	B06	 119	Ile-Asn	N-Source, peptide
PM08 	A11	 118	Glu-Ala	N-Source, peptide
PM08 	C12	 118	Pro-Asn	N-Source, peptide
PM08 	H11	 118	Phe-Gly-Gly	N-Source, peptide
PM03B	H10	 117	Gly-Glu	N-Source, peptide
PM08 	B02	 117	Gly-Ile	N-Source, peptide
PM08 	D11	 116	Thr-Asp	N-Source, peptide
PM06 	H05	 115	Leu-Arg	N-Source, peptide
PM08 	H03	 115	Gly-Gly-Gly	N-Source, peptide
PM08 	H06	 114	Gly-Gly-Phe	N-Source, peptide
PM06 	B07	 113	Arg-Asp	N-Source, peptide
PM08 	C06	 113	Phe-Glu	N-Source, peptide
PM08 	C11	 113	Pro-Arg	N-Source, peptide
PM06 	D04	 112	Cys-Gly	N-Source, peptide
PM06 	E08	 112	Gly-Lys	N-Source, peptide
PM07 	B07	 112	Met-Arg	N-Source, peptide
PM08 	H08	 112	Gly-Phe-Phe	N-Source, peptide
PM08 	C01	 111	Lys-Gly	N-Source, peptide
PM07 	A07	 110	Lys-Arg	N-Source, peptide
PM07 	B11	 110	Met-Gly	N-Source, peptide
PM07 	D07	 110	Pro-Gly	N-Source, peptide
PM08 	B01	 110	Gly-Asp	N-Source, peptide
PM06 	C03	 109	Arg-Ser	N-Source, peptide
PM08 	C05	 109	Phe-Asp	N-Source, peptide
PM06 	E03	 108	Gly-Arg	N-Source, peptide
PM06 	E09	 108	Gly-Met	N-Source, peptide
PM06 	F05	 108	His-Asp	N-Source, peptide
PM06 	G05	 108	Ile-Gln	N-Source, peptide
PM07 	B03	 108	Lys-Thr	N-Source, peptide
PM06 	G04	 107	Ile-Arg	N-Source, peptide
PM07 	B01	 107	Lys-Pro	N-Source, peptide
PM07 	D06	 107	Pro-Gln	N-Source, peptide
PM08 	C08	 107	Phe-Met	N-Source, peptide
PM06 	E10	 106	Gly-Phe	N-Source, peptide
PM06 	E12	 105	Gly-Ser	N-Source, peptide
PM07 	B04	 105	Lys-Trp	N-Source, peptide
PM08 	B07	 105	Ile-Leu	N-Source, peptide
PM08 	C02	 105	Lys-Met	N-Source, peptide
PM08 	F08	 105	D-Ala-Gly	N-Source, peptide
PM07 	E06	 104	Ser-Phe	N-Source, peptide
PM03B	H08	 103	Gly-Asn	N-Source, peptide
PM07 	G08	 103	Tyr-Gly	N-Source, peptide
PM08 	D07	 103	Ser-Asn	N-Source, peptide
PM06 	D11	 102	Glu-Trp	N-Source, peptide
PM06 	D09	 101	Glu-Gly	N-Source, peptide
PM06 	F01	 101	Gly-Thr	N-Source, peptide
PM06 	G06	 101	Ile-Gly	N-Source, peptide
PM06 	G12	 101	Ile-Ser	N-Source, peptide
PM07 	A09	 100	Lys-Ile	N-Source, peptide
PM07 	E02	 100	Ser-Gly	N-Source, peptide
PM07 	F03	 100	Thr-Leu	N-Source, peptide
PM08 	D12	 100	Thr-Gln	N-Source, peptide
PM08 	H04	  99	Gly-Gly-Ile	N-Source, peptide
PM08 	H12	  99	Tyr-Gly-Gly	N-Source, peptide
PM06 	A06	  98	Ala-Glu	N-Source, peptide
PM06 	E06	  98	Gly-His	N-Source, peptide
PM08 	D08	  98	Ser-Asp	N-Source, peptide
PM06 	C01	  97	Arg-Met	N-Source, peptide
PM06 	G03	  97	Ile-Ala	N-Source, peptide
PM06 	B05	  96	Arg-Ala	N-Source, peptide
PM06 	D07	  96	Glu-Asp	N-Source, peptide
PM06 	F06	  96	His-Gly	N-Source, peptide
PM07 	B10	  96	Met-Glu	N-Source, peptide
PM08 	A05	  96	Ala-Ile	N-Source, peptide
PM08 	B04	  96	His-Glu	N-Source, peptide
PM08 	B10	  96	Leu-Pro	N-Source, peptide
PM06 	A04	  95	Ala-Arg	N-Source, peptide
PM06 	C10	  95	Asp-Glu	N-Source, peptide
PM07 	F04	  95	Thr-Met	N-Source, peptide
PM08 	H01	  95	Gly-Gly-Ala	N-Source, peptide
PM03B	H05	  94	Ala-His	N-Source, peptide
PM06 	B12	  94	Arg-Lys	N-Source, peptide
PM06 	C07	  94	Asn-Glu	N-Source, peptide
PM06 	E05	  94	Gly-Gly	N-Source, peptide
PM07 	C10	  94	Phe-Gly	N-Source, peptide
PM08 	A06	  94	Ala-Met	N-Source, peptide
PM08 	B09	  94	Leu-His	N-Source, peptide
PM06 	B10	  93	Arg-Ile	N-Source, peptide
PM07 	A08	  93	Lys-Glu	N-Source, peptide
PM07 	E03	  93	Ser-His	N-Source, peptide
PM08 	B05	  93	His-His	N-Source, peptide
PM06 	C02	  92	Arg-Phe	N-Source, peptide
PM08 	G11	  92	Ala-Ala-Ala	N-Source, peptide
PM08 	H05	  92	Gly-Gly-Leu	N-Source, peptide
PM08 	D10	  91	Ser-Glu	N-Source, peptide
PM07 	F01	  90	Thr-Glu	N-Source, peptide
PM08 	A03	  90	Ala-Asp	N-Source, peptide
PM06 	D08	  89	Glu-Glu	N-Source, peptide
PM06 	E04	  89	Gly-Cys	N-Source, peptide
PM07 	B08	  89	Met-Asp	N-Source, peptide
PM06 	A08	  88	Ala-His	N-Source, peptide
PM06 	C05	  86	Arg-Tyr	N-Source, peptide
PM08 	C03	  85	Met-Thr	N-Source, peptide
PM07 	C03	  84	Met-Lys	N-Source, peptide
PM06 	D12	  83	Glu-Tyr	N-Source, peptide
PM07 	F02	  83	Thr-Gly	N-Source, peptide
PM07 	G11	  83	Tyr-Lys	N-Source, peptide
PM08 	D05	  83	Pro-Trp	N-Source, peptide
PM06 	B09	  80	Arg-Glu	N-Source, peptide
PM07 	D04	  80	Pro-Ala	N-Source, peptide
PM03B	H03	  79	Ala-Glu	N-Source, peptide
PM06 	A10	  79	Ala-Lys	N-Source, peptide
PM07 	D02	  79	Phe-Ser	N-Source, peptide
PM08 	B03	  79	His-Ala	N-Source, peptide
PM08 	D04	  79	Pro-Ser	N-Source, peptide
PM07 	A10	  78	Lys-Leu	N-Source, peptide
PM07 	C02	  78	Met-Leu	N-Source, peptide
PM07 	C05	  78	Met-Phe	N-Source, peptide
PM07 	C09	  78	Phe-Ala	N-Source, peptide
PM06 	C09	  77	Asp-Asp	N-Source, peptide
PM06 	E11	  77	Gly-Pro	N-Source, peptide
PM06 	F03	  77	Gly-Tyr	N-Source, peptide
PM08 	E02	  77	Thr-Ser	N-Source, peptide
PM06 	B06	  75	Arg-Arg	N-Source, peptide
PM06 	B08	  75	Arg-Gln	N-Source, peptide
PM06 	H06	  75	Leu-Asp	N-Source, peptide
PM07 	D08	  75	Pro-Hyp	N-Source, peptide
PM07 	E09	  75	Ser-Tyr	N-Source, peptide
PM06 	A03	  74	Ala-Ala	N-Source, peptide
PM07 	C04	  73	Met-Met	N-Source, peptide
PM08 	D09	  73	Ser-Gln	N-Source, peptide
PM08 	E01	  73	Thr-Phe	N-Source, peptide
PM06 	H11	  72	Leu-Met	N-Source, peptide
PM07 	G10	  72	Tyr-Leu	N-Source, peptide
PM08 	G12	  72	D-Ala-Gly-Gly	N-Source, peptide
PM06 	A07	  71	Ala-Gly	N-Source, peptide
PM06 	C04	  71	Arg-Trp	N-Source, peptide
PM06 	F08	  71	His-Lys	N-Source, peptide
PM06 	B02	  70	Ala-Thr	N-Source, peptide
PM09 	G02,G03,G04	 266	200mM Sodium Phosphate pH 7	osmotic sensitivity; sodium phosphate
PM04A	A03	 103	Pyrophosphate	P-Source, inorganic
PM04A	A04	 101	Trimetaphosphate	P-Source, inorganic
PM04A	A02	 100	Phosphate	P-Source, inorganic
PM04A	B06	 123	D-2-Phospho-Glyceric Acid	P-Source, organic
PM04A	A12	 121	Adenosine 3`,5`-Cyclic Monophosphate	P-Source, organic
PM04A	B07	 119	D-3-Phospho-Glyceric Acid	P-Source, organic
PM04A	E01	 115	O-Phospho-D-Tyrosine	P-Source, organic
PM04A	B05	 114	Carbamyl Phosphate	P-Source, organic
PM04A	C07	 114	6-Phospho-Gluconic Acid	P-Source, organic
PM04A	E05	 114	O-Phosphoryl-Ethanolamine	P-Source, organic
PM04A	D04	 113	Phospho-L-Arginine	P-Source, organic
PM04A	D07	 109	O-Phospho-L-Threonine	P-Source, organic
PM04A	E11	 108	Inositol Hexaphosphate	P-Source, organic
PM04A	C02	 102	Phospho-Glycolic Acid	P-Source, organic
PM04A	D05	 101	O-Phospho-D-Serine	P-Source, organic
PM04A	E04	 101	Phosphoryl Choline	P-Source, organic
PM04A	E10	  98	Thymidine 5`-Monophosphate	P-Source, organic
PM04A	A08	  97	Adenosine 2`-Monophosphate	P-Source, organic
PM04A	D01	  92	D-Mannose-1-Phosphate	P-Source, organic
PM04A	E09	  90	Thymidine 3`-Monophosphate	P-Source, organic
PM04A	D03	  85	Cysteamine-S-Phosphate	P-Source, organic
PM04A	C01	  80	Phosphoenol Pyruvate	P-Source, organic
PM04A	B11	  73	Guanosine 2`,3`-Cyclic Monophosphate	P-Source, organic
PM10 	E04	 154	pH 9.5 + L-Asparagine	pH, deaminase
PM10 	E08	 140	pH 9.5 + Glycine	pH, deaminase
PM10 	E03	 138	pH 9.5 + L-Arginine	pH, deaminase
PM10 	F04	 137	pH 9.5 + L-Serine	pH, deaminase
PM10 	E07	 130	pH 9.5 + L-Glutamine	pH, deaminase
PM10 	E12	 130	pH 9.5 + L-Lysine	pH, deaminase
PM10 	F05	 129	pH 9.5 + L-Threonine	pH, deaminase
PM10 	E06	 128	pH 9.5 + L-Glutamic Acid	pH, deaminase
PM10 	F09	 121	pH 9.5 + Hydroxy-L-Proline	pH, deaminase
PM10 	G01	 121	pH 9.5 + Anthranilic Acid	pH, deaminase
PM10 	E05	 120	pH 9.5 + L-Aspartic Acid	pH, deaminase
PM10 	G12	 111	pH 9.5 + Urea	pH, deaminase
PM10 	E09	 108	pH 9.5 + L-Histidine	pH, deaminase
PM10 	F01	 107	pH 9.5 + L-Methionine	pH, deaminase
PM10 	F03	  93	pH 9.5 + L-Proline	pH, deaminase
PM10 	G10	  89	pH 9.5 + Creatine	pH, deaminase
PM10 	E10	  84	pH 9.5 + L-Isoleucine	pH, deaminase
PM10 	A06	  79	pH 6	pH, growth at 6
PM19 	F10	  81	Sodium caprylate	respiration, ionophore, H+
PM04A	F03	 116	Thiosulfate	S-Source, inorganic
PM04A	F04	 110	Tetrathionate	S-Source, inorganic
PM04A	F02	  94	Sulfate	S-Source, inorganic
PM04A	F05	  82	Thiophosphate	S-Source, inorganic
PM04A	F08	 128	D-Cysteine	S-Source, organic
PM04A	F09	 127	Cys-Gly	S-Source, organic
PM04A	G05	 115	Glutathione	S-Source, organic
PM04A	F12	 111	L-Cysteine Sulfinic Acid	S-Source, organic
PM04A	G04	 103	Lanthionine	S-Source, organic
PM04A	F06	  93	Dithiophosphate	S-Source, organic
PM04A	H06	  82	Taurine	S-Source, organic
PM04A	F07	  79	L-Cysteine	S-Source, organic
PM04A	G03	  79	Cystathionine	S-Source, organic
PM04A	H07	  77	Hypotaurine	S-Source, organic
PM04A	H01	  76	L-Djenkolic Acid	S-Source, organic
PM04A	G08	  75	D-Methionine	S-Source, organic
PM04A	G11	  73	L-Methionine Sulfoxide	S-Source, organic
PM04A	G09	  70	Gly-Met	S-Source, organic
PM14A	G09,G10	 166	Nitrite	toxic anion
PM14A	E09,E10	 138	Sodium metaborate	toxic anion
PM09 	H07,H08,H09	 295	100mM Sodium Nitrite	toxicity, nitrite

Phenotypes Lost:
PM17A	C09,C10	-154	Sulfamonomethoxine	folate antagonist
PM17A	C05,C06	-125	Sulfachloropyridazine	folate antagonist, PABA analog
PM12B	E02,E03	-142	2,4-Diamino-6,7-diisopropylpteridine	folate antagonist, vibriostatic agent
PM19 	E10	 -90	Lawsone	oxidizing agent


Appendix:

         Carbon and Nutrient Max Read Hour: 024
Nitrogen, Phosphorus, Sulfur Max Read Hour: 048
                 Sensitivity Max Read Hour: 024

              Metabolic Distance Threshold: 070
            Sensitivity Distance Threshold: 070

Test Data Files:
Replicate 1:
PM01 : J:\Service\Customer\300\313\data\assay\7983\ecol_SIDx5935xppkxxppxx___1_37_983_106_Junx03x2010_B_11A.oka
Replicate 2:
PM01 : J:\Service\Customer\300\313\data\assay\7994\ecol_SIDx5935xppkxxxxppxx___1_37_994_106_Junx09x2010_B_11A.oka
Replicate 1:
PM02A: J:\Service\Customer\300\313\data\assay\7994\ecol_SIDx5935xppkxxxxppxx___2_37_994_106_Junx09x2010_B_11B.oka
Replicate 2:
PM02A: J:\Service\Customer\300\313\data\assay\7983\ecol_SIDx5935xppkxxppxx___2_37_983_106_Junx03x2010_B_11B.oka
Replicate 1:
PM03B: J:\Service\Customer\300\313\data\assay\7994\ecol_SIDx5935xppkxxxxppxx___3_37_994_106_Junx09x2010_B_12A.oka
Replicate 2:
PM03B: J:\Service\Customer\300\313\data\assay\7983\ecol_SIDx5935xppkxxppxx___3_37_983_106_Junx03x2010_B_12A.oka
Replicate 1:
PM04A: J:\Service\Customer\300\313\data\assay\7983\ecol_SIDx5935xppkxxppxx___4_37_983_106_Junx03x2010_B_12B.oka
Replicate 2:
PM04A: J:\Service\Customer\300\313\data\assay\7994\ecol_SIDx5935xppkxxxxppxx___4_37_994_106_Junx09x2010_B_12B.oka
Replicate 1:
PM05 : J:\Service\Customer\300\313\data\assay\7994\ecol_SIDx5935xppkxxxxppxx___5_37_994_106_Junx09x2010_B_13A.oka
Replicate 2:
PM05 : J:\Service\Customer\300\313\data\assay\7983\ecol_SIDx5935xppkxxppxx___5_37_983_106_Junx03x2010_B_13A.oka
Replicate 1:
PM06 : J:\Service\Customer\300\313\data\assay\7983\ecol_SIDx5935xppkxxppxx___6_37_983_106_Junx03x2010_B_13B.oka
Replicate 2:
PM06 : J:\Service\Customer\300\313\data\assay\7994\ecol_SIDx5935xppkxxxxppxx___6_37_994_106_Junx09x2010_B_13B.oka
Replicate 1:
PM07 : J:\Service\Customer\300\313\data\assay\7983\ecol_SIDx5935xppkxxppxx___7_37_983_106_Junx03x2010_B_14A.oka
Replicate 2:
PM07 : J:\Service\Customer\300\313\data\assay\7994\ecol_SIDx5935xppkxxxxppxx___7_37_994_106_Junx09x2010_B_14A.oka
Replicate 1:
PM08 : J:\Service\Customer\300\313\data\assay\7983\ecol_SIDx5935xppkxxppxx___8_37_983_106_Junx03x2010_B_14B.oka
Replicate 2:
PM08 : J:\Service\Customer\300\313\data\assay\7994\ecol_SIDx5935xppkxxxxppxx___8_37_994_106_Junx09x2010_B_14B.oka
Replicate 1:
PM09 : J:\Service\Customer\300\313\data\assay\7983\ecol_SIDx5935xppkxxppxx___9_37_983_106_Junx03x2010_B_15A.oka
Replicate 2:
PM09 : J:\Service\Customer\300\313\data\assay\7994\ecol_SIDx5935xppkxxxxppxx___9_37_994_106_Junx09x2010_B_15A.oka
Replicate 1:
PM10 : J:\Service\Customer\300\313\data\assay\7994\ecol_SIDx5935xppkxxxxppxx___10_37_994_106_Junx09x2010_B_15B.oka
Replicate 2:
PM10 : J:\Service\Customer\300\313\data\assay\7983\ecol_SIDx5935xppkxxppxx___10_37_983_106_Junx03x2010_B_15B.oka
Replicate 1:
PM11C: J:\Service\Customer\300\313\data\assay\7994\ecol_SIDx5935xppkxxxxppxx___11_37_994_106_Junx09x2010_B_16A.oka
Replicate 2:
PM11C: J:\Service\Customer\300\313\data\assay\7983\ecol_SIDx5935xppkxxppxx___11_37_983_106_Junx03x2010_B_16A.oka
Replicate 1:
PM12B: J:\Service\Customer\300\313\data\assay\7983\ecol_SIDx5935xppkxxppxx___12_37_983_106_Junx03x2010_B_16B.oka
Replicate 2:
PM12B: J:\Service\Customer\300\313\data\assay\7994\ecol_SIDx5935xppkxxxxppxx___12_37_994_106_Junx09x2010_B_16B.oka
Replicate 1:
PM13B: J:\Service\Customer\300\313\data\assay\7994\ecol_SIDx5935xppkxxxxppxx___13_37_994_106_Junx09x2010_B_17A.oka
Replicate 2:
PM13B: J:\Service\Customer\300\313\data\assay\7983\ecol_SIDx5935xppkxxppxx___13_37_983_106_Junx03x2010_B_17A.oka
Replicate 1:
PM14A: J:\Service\Customer\300\313\data\assay\7994\ecol_SIDx5935xppkxxxxppxx___14_37_994_106_Junx09x2010_B_17B.oka
Replicate 2:
PM14A: J:\Service\Customer\300\313\data\assay\7983\ecol_SIDx5935xppkxxppxx___14_37_983_106_Junx03x2010_B_17B.oka
Replicate 1:
PM15B: J:\Service\Customer\300\313\data\assay\7994\ecol_SIDx5935xppkxxxxppxx___15_37_994_106_Junx09x2010_B_18A.oka
Replicate 2:
PM15B: J:\Service\Customer\300\313\data\assay\7983\ecol_SIDx5935xppkxxppxx___15_37_983_106_Junx03x2010_B_18A.oka
Replicate 1:
PM16A: J:\Service\Customer\300\313\data\assay\7994\ecol_SIDx5935xppkxxxxppxx___16_37_994_106_Junx09x2010_B_18B.oka
Replicate 2:
PM16A: J:\Service\Customer\300\313\data\assay\7983\ecol_SIDx5935xppkxxppxx___16_37_983_106_Junx03x2010_B_18B.oka
Replicate 1:
PM17A: J:\Service\Customer\300\313\data\assay\7983\ecol_SIDx5935xppkxxppxx___17_37_983_106_Junx03x2010_B_19A.oka
Replicate 2:
PM17A: J:\Service\Customer\300\313\data\assay\7994\ecol_SIDx5935xppkxxxxppxx___17_37_994_106_Junx09x2010_B_19A.oka
Replicate 1:
PM18C: J:\Service\Customer\300\313\data\assay\7994\ecol_SIDx5935xppkxxxxppxx___18_37_994_106_Junx09x2010_B_19B.oka
Replicate 2:
PM18C: J:\Service\Customer\300\313\data\assay\7983\ecol_SIDx5935xppkxxppxx___18_37_983_106_Junx03x2010_B_19B.oka
Replicate 1:
PM19 : J:\Service\Customer\300\313\data\assay\7994\ecol_SIDx5935xppkxxxxppxx___19_37_994_106_Junx09x2010_B_20A.oka
Replicate 2:
PM19 : J:\Service\Customer\300\313\data\assay\7983\ecol_SIDx5935xppkxxppxx___19_37_983_106_Junx03x2010_B_20A.oka
Replicate 1:
PM20B: J:\Service\Customer\300\313\data\assay\7994\ecol_SIDx5935xppkxxxxppxx___20_37_994_106_Junx09x2010_B_20B.oka
Replicate 2:
PM20B: J:\Service\Customer\300\313\data\assay\7983\ecol_SIDx5935xppkxxppxx___20_37_983_106_Junx03x2010_B_20B.oka

Reference Data Files:
Replicate 1:
PM01 : J:\Service\Customer\300\313\data\assay\7991\ecol_SIDx5932xK12___1_37_991_1_Junx09x2010_A_01A.oka
Replicate 2:
PM01 : J:\Service\Customer\300\313\data\assay\7980\ecol_SIDx5932xK12___1_37_980_1_Junx03x2010_A_01A.oka
Replicate 1:
PM02A: J:\Service\Customer\300\313\data\assay\7991\ecol_SIDx5932xK12___2_37_991_1_Junx09x2010_A_01B.oka
Replicate 2:
PM02A: J:\Service\Customer\300\313\data\assay\7980\ecol_SIDx5932xK12___2_37_980_1_Junx03x2010_A_01B.oka
Replicate 1:
PM03B: J:\Service\Customer\300\313\data\assay\7991\ecol_SIDx5932xK12___3_37_991_1_Junx09x2010_A_02A.oka
Replicate 2:
PM03B: J:\Service\Customer\300\313\data\assay\7980\ecol_SIDx5932xK12___3_37_980_1_Junx03x2010_A_02A.oka
Replicate 1:
PM04A: J:\Service\Customer\300\313\data\assay\7991\ecol_SIDx5932xK12___4_37_991_1_Junx09x2010_A_02B.oka
Replicate 2:
PM04A: J:\Service\Customer\300\313\data\assay\7980\ecol_SIDx5932xK12___4_37_980_1_Junx03x2010_A_02B.oka
Replicate 1:
PM05 : J:\Service\Customer\300\313\data\assay\7991\ecol_SIDx5932xK12___5_37_991_1_Junx09x2010_A_03A.oka
Replicate 2:
PM05 : J:\Service\Customer\300\313\data\assay\7980\ecol_SIDx5932xK12___5_37_980_1_Junx03x2010_A_03A.oka
Replicate 1:
PM06 : J:\Service\Customer\300\313\data\assay\7991\ecol_SIDx5932xK12___6_37_991_1_Junx09x2010_A_03B.oka
Replicate 2:
PM06 : J:\Service\Customer\300\313\data\assay\7980\ecol_SIDx5932xK12___6_37_980_1_Junx03x2010_A_03B.oka
Replicate 1:
PM07 : J:\Service\Customer\300\313\data\assay\7980\ecol_SIDx5932xK12___7_37_980_1_Junx03x2010_A_04A.oka
Replicate 2:
PM07 : J:\Service\Customer\300\313\data\assay\7991\ecol_SIDx5932xK12___7_37_991_1_Junx09x2010_A_04A.oka
Replicate 1:
PM08 : J:\Service\Customer\300\313\data\assay\7991\ecol_SIDx5932xK12___8_37_991_1_Junx09x2010_A_04B.oka
Replicate 2:
PM08 : J:\Service\Customer\300\313\data\assay\7980\ecol_SIDx5932xK12___8_37_980_1_Junx03x2010_A_04B.oka
Replicate 1:
PM09 : J:\Service\Customer\300\313\data\assay\7991\ecol_SIDx5932xK12___9_37_991_1_Junx09x2010_A_05A.oka
Replicate 2:
PM09 : J:\Service\Customer\300\313\data\assay\7980\ecol_SIDx5932xK12___9_37_980_1_Junx03x2010_A_05A.oka
Replicate 1:
PM10 : J:\Service\Customer\300\313\data\assay\7980\ecol_SIDx5932xK12___10_37_980_1_Junx03x2010_A_05B.oka
Replicate 2:
PM10 : J:\Service\Customer\300\313\data\assay\7991\ecol_SIDx5932xK12___10_37_991_1_Junx09x2010_A_05B.oka
Replicate 1:
PM11C: J:\Service\Customer\300\313\data\assay\7991\ecol_SIDx5932xK12___11_37_991_1_Junx09x2010_A_06A.oka
Replicate 2:
PM11C: J:\Service\Customer\300\313\data\assay\7980\ecol_SIDx5932xK12___11_37_980_1_Junx03x2010_A_06A.oka
Replicate 1:
PM12B: J:\Service\Customer\300\313\data\assay\7980\ecol_SIDx5932xK12___12_37_980_1_Junx03x2010_A_06B.oka
Replicate 2:
PM12B: J:\Service\Customer\300\313\data\assay\7991\ecol_SIDx5932xK12___12_37_991_1_Junx09x2010_A_06B.oka
Replicate 1:
PM13B: J:\Service\Customer\300\313\data\assay\7991\ecol_SIDx5932xK12___13_37_991_1_Junx09x2010_A_07A.oka
Replicate 2:
PM13B: J:\Service\Customer\300\313\data\assay\7980\ecol_SIDx5932xK12___13_37_980_1_Junx03x2010_A_07A.oka
Replicate 1:
PM14A: J:\Service\Customer\300\313\data\assay\7991\ecol_SIDx5932xK12___14_37_991_1_Junx09x2010_A_07B.oka
Replicate 2:
PM14A: J:\Service\Customer\300\313\data\assay\7980\ecol_SIDx5932xK12___14_37_980_1_Junx03x2010_A_07B.oka
Replicate 1:
PM15B: J:\Service\Customer\300\313\data\assay\7991\ecol_SIDx5932xK12___15_37_991_1_Junx09x2010_A_08A.oka
Replicate 2:
PM15B: J:\Service\Customer\300\313\data\assay\7980\ecol_SIDx5932xK12___15_37_980_1_Junx03x2010_A_08A.oka
Replicate 1:
PM16A: J:\Service\Customer\300\313\data\assay\7980\ecol_SIDx5932xK12___16_37_980_1_Junx03x2010_A_08B.oka
Replicate 2:
PM16A: J:\Service\Customer\300\313\data\assay\7991\ecol_SIDx5932xK12___16_37_991_1_Junx09x2010_A_08B.oka
Replicate 1:
PM17A: J:\Service\Customer\300\313\data\assay\7991\ecol_SIDx5932xK12___17_37_991_1_Junx09x2010_A_09A.oka
Replicate 2:
PM17A: J:\Service\Customer\300\313\data\assay\7980\ecol_SIDx5932xK12___17_37_980_1_Junx03x2010_A_09A.oka
Replicate 1:
PM18C: J:\Service\Customer\300\313\data\assay\7991\ecol_SIDx5932xK12___18_37_991_1_Junx09x2010_A_09B.oka
Replicate 2:
PM18C: J:\Service\Customer\300\313\data\assay\7980\ecol_SIDx5932xK12___18_37_980_1_Junx03x2010_A_09B.oka
Replicate 1:
PM19 : J:\Service\Customer\300\313\data\assay\7991\ecol_SIDx5932xK12___19_37_991_1_Junx09x2010_A_10A.oka
Replicate 2:
PM19 : J:\Service\Customer\300\313\data\assay\7980\ecol_SIDx5932xK12___19_37_980_1_Junx03x2010_A_10A.oka
Replicate 1:
PM20B: J:\Service\Customer\300\313\data\assay\7991\ecol_SIDx5932xK12___20_37_991_1_Junx09x2010_A_10B.oka
Replicate 2:
PM20B: J:\Service\Customer\300\313\data\assay\7980\ecol_SIDx5932xK12___20_37_980_1_Junx03x2010_A_10B.oka
```
