# Supplementary material for: Datasets for transcriptomics, q-proteomics and phenotype microarrays of polyphosphate metabolism mutants from Escherichia coli
Source: Data Brief. 2017 Mar 18;12:13–7. doi: 10.1016/j.dib.2017.03.010 (PMC5367803; doi:10.1016/j.dib.2017.03.010)
Supplement: Supplementary file 2 — Supplementary material [file mmc2.zip › BBAGEN-16-480/Ecoli ppk_versus_EcoliK12.doc]

```
EcolSIDx5933xppkx versus EcolSIDx5932xK12


BiOLOG
21124 Cabot Blvd.
Hayward, CA 94545


                    Phenotype MicroArray Analysis
              Test: Ecol_SID_5933_ppk-
         Reference: Ecol_SID_5932_K12
       Report Date: 6/16/2010 11:10:41 AM


Reproducibiltiy Analysis:

Test                                               Reference
PM01:    00: passes                      PM01:    01: passes
PM02:    00: passes                      PM02:    00: passes
PM03:    00: passes                      PM03:    01: passes
PM04:    04: passes                      PM04:    00: passes
PM05:    00: passes                      PM05:    00: passes
PM06:    00: passes                      PM06:    00: passes
PM07:    00: passes                      PM07:    00: passes
PM08:    02: passes                      PM08:    00: passes
PM09:    00: passes                      PM09:    00: passes
PM10:    00: passes                      PM10:    00: passes
PM11:    00: passes                      PM11:    00: passes
PM12:    00: passes                      PM12:    01: passes
PM13:    00: passes                      PM13:    00: passes
PM14:    00: passes                      PM14:    00: passes
PM15:    00: passes                      PM15:    00: passes
PM16:    01: passes                      PM16:    00: passes
PM17:    02: passes                      PM17:    02: passes
PM18:    00: passes                      PM18:    00: passes
PM19:    01: passes                      PM19:    00: passes
PM20:    01: passes                      PM20:    00: passes
PM21:    -1:    N/A                      PM21:    -1:    N/A
PM22:    -1:    N/A                      PM22:    -1:    N/A
PM23:    -1:    N/A                      PM23:    -1:    N/A
PM24:    -1:    N/A                      PM24:    -1:    N/A
PM25:    -1:    N/A                      PM25:    -1:    N/A
PM26:    -1:    N/A                      PM26:    -1:    N/A
PM27:    -1:    N/A                      PM27:    -1:    N/A
PM28:    -1:    N/A                      PM28:    -1:    N/A
PM29:    -1:    N/A                      PM29:    -1:    N/A
PM30:    -1:    N/A                      PM30:    -1:    N/A
PM31:    -1:    N/A                      PM31:    -1:    N/A
PM32:    -1:    N/A                      PM32:    -1:    N/A
PM33:    -1:    N/A                      PM33:    -1:    N/A
PM34:    -1:    N/A                      PM34:    -1:    N/A
PM35:    -1:    N/A                      PM35:    -1:    N/A
PM36:    -1:    N/A                      PM36:    -1:    N/A
PM37:    -1:    N/A                      PM37:    -1:    N/A
PM38:    -1:    N/A                      PM38:    -1:    N/A
PM39:    -1:    N/A                      PM39:    -1:    N/A
PM40:    -1:    N/A                      PM40:    -1:    N/A


Replicate 1 versus Replicate 2 of Test:
Ecol_SID_5933_ppk-
```

Replicate 1 versus Replicate 2 of Reference:

Ecol_SID_5932_K12

```
Run 1:
Ecol_SID_5933_ppk-( green )
 versus
Ecol_SID_5932_K12( red )
```

```
Run 2:
Ecol_SID_5933_ppk-( green )
 versus
Ecol_SID_5932_K12( red )
```

```
Consensus:
Ecol_SID_5933_ppk-( green )
 versus
Ecol_SID_5932_K12( red )
```

```
PM Report:
Ecol_SID_5933_ppk-_
 versus
Ecol_SID_5932_K12_

Phenotypes Gained:
PM01 	A07	 106	L-Aspartic Acid	C-Source, amino acid
PM01 	A08	  81	L-Proline	C-Source, amino acid
PM01 	C03	 103	D,L-Malic Acid	C-Source, carboxylic acid
PM01 	G11	 101	D-Malic Acid	C-Source, carboxylic acid
PM01 	G12	 101	L-Malic Acid	C-Source, carboxylic acid
PM01 	F07	  92	Propionic Acid	C-Source, carboxylic acid
PM01 	G09	  87	Mono-Methylsuccinate	C-Source, carboxylic acid
PM01 	F05	  83	Fumaric Acid	C-Source, carboxylic acid
PM03B	A09	 150	L-Asparagine	N-Source, amino acid
PM03B	A08	 146	L-Arginine	N-Source, amino acid
PM03B	A10	 141	L-Aspartic Acid	N-Source, amino acid
PM03B	B02	 137	Glycine	N-Source, amino acid
PM03B	B08	 114	L-Phenylalanine	N-Source, amino acid
PM03B	C04	 109	D-Asparagine	N-Source, amino acid
PM03B	C12	 109	L-Ornithine	N-Source, amino acid
PM03B	C10	  91	L-Citrulline	N-Source, amino acid
PM03B	A07	  88	L-Alanine	N-Source, amino acid
PM03B	B07	  86	L-Methionine	N-Source, amino acid
PM03B	C09	  86	D-Valine	N-Source, amino acid
PM03B	C01	  84	L-Tyrosine	N-Source, amino acid
PM03B	A12	  80	L-Glutamic Acid	N-Source, amino acid
PM03B	A02	 138	Ammonia	N-Source, inorganic
PM03B	F05	 114	Cytosine	N-Source, other
PM03B	G11	 102	d-Amino-N-Valeric Acid	N-Source, other
PM03B	D11	  98	Putrescine	N-Source, other
PM03B	G08	  92	g-Amino-N-Butyric Acid	N-Source, other
PM06 	H05	 123	Leu-Arg	N-Source, peptide
PM08 	A09	 118	Asp-Gln	N-Source, peptide
PM07 	D05	 115	Pro-Asp	N-Source, peptide
PM03B	H10	 114	Gly-Glu	N-Source, peptide
PM08 	B01	 114	Gly-Asp	N-Source, peptide
PM07 	C05	 113	Met-Phe	N-Source, peptide
PM08 	B06	 113	Ile-Asn	N-Source, peptide
PM08 	C07	 113	Gln-Glu	N-Source, peptide
PM07 	B03	 112	Lys-Thr	N-Source, peptide
PM08 	A10	 112	Asp-Gly	N-Source, peptide
PM08 	B02	 112	Gly-Ile	N-Source, peptide
PM08 	C08	 111	Phe-Met	N-Source, peptide
PM03B	H11	 110	Gly-Met	N-Source, peptide
PM08 	A08	 110	Asp-Ala	N-Source, peptide
PM08 	C06	 110	Phe-Glu	N-Source, peptide
PM08 	C01	 109	Lys-Gly	N-Source, peptide
PM08 	C05	 109	Phe-Asp	N-Source, peptide
PM07 	G08	 108	Tyr-Gly	N-Source, peptide
PM07 	B01	 107	Lys-Pro	N-Source, peptide
PM08 	H06	 107	Gly-Gly-Phe	N-Source, peptide
PM06 	C11	 106	Asp-Leu	N-Source, peptide
PM06 	F05	 106	His-Asp	N-Source, peptide
PM07 	D07	 106	Pro-Gly	N-Source, peptide
PM07 	C02	 105	Met-Leu	N-Source, peptide
PM08 	H05	 105	Gly-Gly-Leu	N-Source, peptide
PM06 	C01	 104	Arg-Met	N-Source, peptide
PM06 	C05	 104	Arg-Tyr	N-Source, peptide
PM06 	H07	 104	Leu-Glu	N-Source, peptide
PM07 	A09	 104	Lys-Ile	N-Source, peptide
PM07 	E05	 104	Ser-Met	N-Source, peptide
PM07 	G07	 104	Tyr-Glu	N-Source, peptide
PM08 	F08	 104	D-Ala-Gly	N-Source, peptide
PM07 	B02	 102	Lys-Ser	N-Source, peptide
PM08 	H12	 101	Tyr-Gly-Gly	N-Source, peptide
PM06 	F03	 100	Gly-Tyr	N-Source, peptide
PM06 	H06	 100	Leu-Asp	N-Source, peptide
PM07 	B07	 100	Met-Arg	N-Source, peptide
PM07 	C11	 100	Phe-Ile	N-Source, peptide
PM08 	B07	 100	Ile-Leu	N-Source, peptide
PM08 	H08	 100	Gly-Phe-Phe	N-Source, peptide
PM06 	C03	  99	Arg-Ser	N-Source, peptide
PM07 	E09	  99	Ser-Tyr	N-Source, peptide
PM08 	B04	  99	His-Glu	N-Source, peptide
PM06 	G04	  98	Ile-Arg	N-Source, peptide
PM08 	B12	  98	Lys-Asp	N-Source, peptide
PM08 	C09	  97	Phe-Tyr	N-Source, peptide
PM06 	B07	  96	Arg-Asp	N-Source, peptide
PM06 	C12	  96	Asp-Lys	N-Source, peptide
PM08 	B09	  96	Leu-His	N-Source, peptide
PM08 	H11	  96	Phe-Gly-Gly	N-Source, peptide
PM06 	E10	  95	Gly-Phe	N-Source, peptide
PM07 	A07	  95	Lys-Arg	N-Source, peptide
PM07 	C01	  95	Met-Ile	N-Source, peptide
PM08 	A11	  95	Glu-Ala	N-Source, peptide
PM06 	D07	  94	Glu-Asp	N-Source, peptide
PM07 	F01	  94	Thr-Glu	N-Source, peptide
PM07 	B11	  93	Met-Gly	N-Source, peptide
PM07 	D06	  93	Pro-Gln	N-Source, peptide
PM06 	C10	  92	Asp-Glu	N-Source, peptide
PM06 	A06	  91	Ala-Glu	N-Source, peptide
PM08 	D11	  91	Thr-Asp	N-Source, peptide
PM06 	B10	  90	Arg-Ile	N-Source, peptide
PM06 	D08	  90	Glu-Glu	N-Source, peptide
PM06 	D11	  90	Glu-Trp	N-Source, peptide
PM07 	A08	  90	Lys-Glu	N-Source, peptide
PM08 	B10	  90	Leu-Pro	N-Source, peptide
PM08 	H04	  90	Gly-Gly-Ile	N-Source, peptide
PM08 	H03	  89	Gly-Gly-Gly	N-Source, peptide
PM06 	E03	  88	Gly-Arg	N-Source, peptide
PM06 	F06	  88	His-Gly	N-Source, peptide
PM06 	G06	  88	Ile-Gly	N-Source, peptide
PM07 	A06	  88	Lys-Ala	N-Source, peptide
PM07 	D12	  88	Pro-Tyr	N-Source, peptide
PM07 	E06	  88	Ser-Phe	N-Source, peptide
PM07 	F03	  88	Thr-Leu	N-Source, peptide
PM07 	F04	  88	Thr-Met	N-Source, peptide
PM07 	G10	  88	Tyr-Leu	N-Source, peptide
PM06 	E06	  87	Gly-His	N-Source, peptide
PM06 	E08	  87	Gly-Lys	N-Source, peptide
PM06 	E09	  87	Gly-Met	N-Source, peptide
PM06 	G12	  87	Ile-Ser	N-Source, peptide
PM08 	C04	  87	Met-Tyr	N-Source, peptide
PM08 	C12	  87	Pro-Asn	N-Source, peptide
PM08 	D08	  87	Ser-Asp	N-Source, peptide
PM06 	C02	  86	Arg-Phe	N-Source, peptide
PM06 	D09	  86	Glu-Gly	N-Source, peptide
PM07 	B10	  86	Met-Glu	N-Source, peptide
PM06 	C07	  85	Asn-Glu	N-Source, peptide
PM06 	D12	  85	Glu-Tyr	N-Source, peptide
PM07 	D10	  85	Pro-Phe	N-Source, peptide
PM06 	D02	  84	Asp-Trp	N-Source, peptide
PM06 	F11	  84	His-Ser	N-Source, peptide
PM06 	G05	  84	Ile-Gln	N-Source, peptide
PM07 	B04	  84	Lys-Trp	N-Source, peptide
PM07 	B08	  84	Met-Asp	N-Source, peptide
PM07 	D08	  84	Pro-Hyp	N-Source, peptide
PM08 	B11	  84	Leu-Tyr	N-Source, peptide
PM07 	D01	  82	Phe-Pro	N-Source, peptide
PM08 	C11	  81	Pro-Arg	N-Source, peptide
PM06 	C09	  80	Asp-Asp	N-Source, peptide
PM04A	A04	 137	Trimetaphosphate	P-Source, inorganic
PM04A	A03	 117	Pyrophosphate	P-Source, inorganic
PM04A	A02	 114	Phosphate	P-Source, inorganic
PM04A	C07	 150	6-Phospho-Gluconic Acid	P-Source, organic
PM04A	A08	 132	Adenosine 2`-Monophosphate	P-Source, organic
PM04A	B05	 125	Carbamyl Phosphate	P-Source, organic
PM04A	B06	 123	D-2-Phospho-Glyceric Acid	P-Source, organic
PM04A	D04	 122	Phospho-L-Arginine	P-Source, organic
PM04A	B07	 121	D-3-Phospho-Glyceric Acid	P-Source, organic
PM04A	E11	 119	Inositol Hexaphosphate	P-Source, organic
PM04A	E02	 118	O-Phospho-L-Tyrosine	P-Source, organic
PM04A	E05	 117	O-Phosphoryl-Ethanolamine	P-Source, organic
PM04A	D07	 112	O-Phospho-L-Threonine	P-Source, organic
PM04A	E01	 110	O-Phospho-D-Tyrosine	P-Source, organic
PM04A	E09	 107	Thymidine 3`-Monophosphate	P-Source, organic
PM04A	A12	 106	Adenosine 3`,5`-Cyclic Monophosphate	P-Source, organic
PM04A	D05	 105	O-Phospho-D-Serine	P-Source, organic
PM04A	E03	 105	Phosphocreatine	P-Source, organic
PM04A	E04	 104	Phosphoryl Choline	P-Source, organic
PM04A	E10	 100	Thymidine 5`-Monophosphate	P-Source, organic
PM04A	D06	  96	O-Phospho-L-Serine	P-Source, organic
PM04A	C12	  85	Cytidine 3`,5`-Cyclic Monophosphate	P-Source, organic
PM04A	F03	 115	Thiosulfate	S-Source, inorganic
PM04A	F02	  98	Sulfate	S-Source, inorganic
PM04A	F04	  94	Tetrathionate	S-Source, inorganic
PM04A	F05	  88	Thiophosphate	S-Source, inorganic
PM04A	F08	 115	D-Cysteine	S-Source, organic
PM04A	F09	 103	Cys-Gly	S-Source, organic
PM04A	H06	 100	Taurine	S-Source, organic
PM04A	G04	  94	Lanthionine	S-Source, organic
PM04A	F12	  93	L-Cysteine Sulfinic Acid	S-Source, organic
PM04A	G03	  91	Cystathionine	S-Source, organic
PM04A	G05	  90	Glutathione	S-Source, organic
PM04A	G08	  86	D-Methionine	S-Source, organic
PM04A	F06	  84	Dithiophosphate	S-Source, organic

Phenotypes Lost:
PM18C	A06,A07	-110	Pyrophosphate	chelator, hydrophilic
PM17A	C09,C10	-130	Sulfamonomethoxine	folate antagonist
PM17A	C05,C06	-115	Sulfachloropyridazine	folate antagonist, PABA analog
PM12B	E02,E03	-152	2,4-Diamino-6,7-diisopropylpteridine	folate antagonist, vibriostatic agent
PM13B	A05	 -61	Dequalinium	ion channel inhibitor, K+
PM14A	A05,A06	-143	Furaltadone	nitro compound, oxidizing agent, DNA damage
PM15B	E05	 -66	Nitrofurazone	nitro compound, oxidizing agent, DNA damage
PM09 	F01	 -79	1% Sodium Lactate	osmotic sensitivity, sodium lactate
PM19 	E09,E10	-176	Lawsone	oxidizing agent
PM10 	G11	-101	pH 9.5 + Trimethylamine-N-Oxide	pH, deaminase
PM10 	A09	 -80	pH 8.5	pH, growth at 8.5
PM14A	G02,G03	-136	Chelerythrine	protein kinase C inhibitor
PM19 	H05,H06	-120	Thioglycerol	reducing agent, thiol, adenosyl methionine antagonist
PM16A	H12	 -68	Sorbic Acid	respiration, ionophore, H+
PM19 	A06	 -62	Gallic acid	respiration, ionophore, H+
PM12B	H08	 -76	Rifampicin	RNA polymerase


Appendix:

         Carbon and Nutrient Max Read Hour: 024
Nitrogen, Phosphorus, Sulfur Max Read Hour: 048
                 Sensitivity Max Read Hour: 024

              Metabolic Distance Threshold: 080
            Sensitivity Distance Threshold: 060

Test Data Files:
Replicate 1:
PM01 : J:\Service\Customer\300\313\data\assay\7981\ecol_SIDx5933xppkx___1_37_981_1_Junx03x2010_B_11A.oka
Replicate 2:
PM01 : J:\Service\Customer\300\313\data\assay\7992\ecol_SIDx5933xppkx___1_37_992_1_Junx09x2010_B_11A.oka
Replicate 1:
PM02A: J:\Service\Customer\300\313\data\assay\7992\ecol_SIDx5933xppkx___2_37_992_1_Junx09x2010_B_11B.oka
Replicate 2:
PM02A: J:\Service\Customer\300\313\data\assay\7981\ecol_SIDx5933xppkx___2_37_981_1_Junx03x2010_B_11B.oka
Replicate 1:
PM03B: J:\Service\Customer\300\313\data\assay\7981\ecol_SIDx5933xppkx___3_37_981_1_Junx03x2010_B_12A.oka
Replicate 2:
PM03B: J:\Service\Customer\300\313\data\assay\7992\ecol_SIDx5933xppkx___3_37_992_1_Junx09x2010_B_12A.oka
Replicate 1:
PM04A: J:\Service\Customer\300\313\data\assay\7992\ecol_SIDx5933xppkx___4_37_992_1_Junx09x2010_B_12B.oka
Replicate 2:
PM04A: J:\Service\Customer\300\313\data\assay\7981\ecol_SIDx5933xppkx___4_37_981_1_Junx03x2010_B_12B.oka
Replicate 1:
PM05 : J:\Service\Customer\300\313\data\assay\7981\ecol_SIDx5933xppkx___5_37_981_1_Junx03x2010_B_13A.oka
Replicate 2:
PM05 : J:\Service\Customer\300\313\data\assay\7992\ecol_SIDx5933xppkx___5_37_992_1_Junx09x2010_B_13A.oka
Replicate 1:
PM06 : J:\Service\Customer\300\313\data\assay\7992\ecol_SIDx5933xppkx___6_37_992_1_Junx09x2010_B_13B.oka
Replicate 2:
PM06 : J:\Service\Customer\300\313\data\assay\7981\ecol_SIDx5933xppkx___6_37_981_1_Junx03x2010_B_13B.oka
Replicate 1:
PM07 : J:\Service\Customer\300\313\data\assay\7992\ecol_SIDx5933xppkx___7_37_992_1_Junx09x2010_B_14A.oka
Replicate 2:
PM07 : J:\Service\Customer\300\313\data\assay\7981\ecol_SIDx5933xppkx___7_37_981_1_Junx03x2010_B_14A.oka
Replicate 1:
PM08 : J:\Service\Customer\300\313\data\assay\7992\ecol_SIDx5933xppkx___8_37_992_1_Junx09x2010_B_14B.oka
Replicate 2:
PM08 : J:\Service\Customer\300\313\data\assay\7981\ecol_SIDx5933xppkx___8_37_981_1_Junx03x2010_B_14B.oka
Replicate 1:
PM09 : J:\Service\Customer\300\313\data\assay\7992\ecol_SIDx5933xppkx___9_37_992_1_Junx09x2010_B_15A.oka
Replicate 2:
PM09 : J:\Service\Customer\300\313\data\assay\7981\ecol_SIDx5933xppkx___9_37_981_1_Junx03x2010_B_15A.oka
Replicate 1:
PM10 : J:\Service\Customer\300\313\data\assay\7981\ecol_SIDx5933xppkx___10_37_981_1_Junx03x2010_B_15B.oka
Replicate 2:
PM10 : J:\Service\Customer\300\313\data\assay\7992\ecol_SIDx5933xppkx___10_37_992_1_Junx09x2010_B_15B.oka
Replicate 1:
PM11C: J:\Service\Customer\300\313\data\assay\7992\ecol_SIDx5933xppkx___11_37_992_1_Junx09x2010_B_16A.oka
Replicate 2:
PM11C: J:\Service\Customer\300\313\data\assay\7981\ecol_SIDx5933xppkx___11_37_981_1_Junx03x2010_B_16A.oka
Replicate 1:
PM12B: J:\Service\Customer\300\313\data\assay\7981\ecol_SIDx5933xppkx___12_37_981_1_Junx03x2010_B_16B.oka
Replicate 2:
PM12B: J:\Service\Customer\300\313\data\assay\7992\ecol_SIDx5933xppkx___12_37_992_1_Junx09x2010_B_16B.oka
Replicate 1:
PM13B: J:\Service\Customer\300\313\data\assay\7992\ecol_SIDx5933xppkx___13_37_992_1_Junx09x2010_B_17A.oka
Replicate 2:
PM13B: J:\Service\Customer\300\313\data\assay\7981\ecol_SIDx5933xppkx___13_37_981_1_Junx03x2010_B_17A.oka
Replicate 1:
PM14A: J:\Service\Customer\300\313\data\assay\7992\ecol_SIDx5933xppkx___14_37_992_1_Junx09x2010_B_17B.oka
Replicate 2:
PM14A: J:\Service\Customer\300\313\data\assay\7981\ecol_SIDx5933xppkx___14_37_981_1_Junx03x2010_B_17B.oka
Replicate 1:
PM15B: J:\Service\Customer\300\313\data\assay\7992\ecol_SIDx5933xppkx___15_37_992_1_Junx09x2010_B_18A.oka
Replicate 2:
PM15B: J:\Service\Customer\300\313\data\assay\7981\ecol_SIDx5933xppkx___15_37_981_1_Junx03x2010_B_18A.oka
Replicate 1:
PM16A: J:\Service\Customer\300\313\data\assay\7981\ecol_SIDx5933xppkx___16_37_981_1_Junx03x2010_B_18B.oka
Replicate 2:
PM16A: J:\Service\Customer\300\313\data\assay\7992\ecol_SIDx5933xppkx___16_37_992_1_Junx09x2010_B_18B.oka
Replicate 1:
PM17A: J:\Service\Customer\300\313\data\assay\7981\ecol_SIDx5933xppkx___17_37_981_1_Junx03x2010_B_19A.oka
Replicate 2:
PM17A: J:\Service\Customer\300\313\data\assay\7992\ecol_SIDx5933xppkx___17_37_992_1_Junx09x2010_B_19A.oka
Replicate 1:
PM18C: J:\Service\Customer\300\313\data\assay\7981\ecol_SIDx5933xppkx___18_37_981_1_Junx03x2010_B_19B.oka
Replicate 2:
PM18C: J:\Service\Customer\300\313\data\assay\7992\ecol_SIDx5933xppkx___18_37_992_1_Junx09x2010_B_19B.oka
Replicate 1:
PM19 : J:\Service\Customer\300\313\data\assay\7992\ecol_SIDx5933xppkx___19_37_992_1_Junx09x2010_B_20A.oka
Replicate 2:
PM19 : J:\Service\Customer\300\313\data\assay\7981\ecol_SIDx5933xppkx___19_37_981_1_Junx03x2010_B_20A.oka
Replicate 1:
PM20B: J:\Service\Customer\300\313\data\assay\7981\ecol_SIDx5933xppkx___20_37_981_1_Junx03x2010_B_20B.oka
Replicate 2:
PM20B: J:\Service\Customer\300\313\data\assay\7992\ecol_SIDx5933xppkx___20_37_992_1_Junx09x2010_B_20B.oka

Reference Data Files:
Replicate 1:
PM01 : J:\Service\Customer\300\313\data\assay\7991\ecol_SIDx5932xK12___1_37_991_1_Junx09x2010_A_01A.oka
Replicate 2:
PM01 : J:\Service\Customer\300\313\data\assay\7980\ecol_SIDx5932xK12___1_37_980_1_Junx03x2010_A_01A.oka
Replicate 1:
PM02A: J:\Service\Customer\300\313\data\assay\7991\ecol_SIDx5932xK12___2_37_991_1_Junx09x2010_A_01B.oka
Replicate 2:
PM02A: J:\Service\Customer\300\313\data\assay\7980\ecol_SIDx5932xK12___2_37_980_1_Junx03x2010_A_01B.oka
Replicate 1:
PM03B: J:\Service\Customer\300\313\data\assay\7991\ecol_SIDx5932xK12___3_37_991_1_Junx09x2010_A_02A.oka
Replicate 2:
PM03B: J:\Service\Customer\300\313\data\assay\7980\ecol_SIDx5932xK12___3_37_980_1_Junx03x2010_A_02A.oka
Replicate 1:
PM04A: J:\Service\Customer\300\313\data\assay\7991\ecol_SIDx5932xK12___4_37_991_1_Junx09x2010_A_02B.oka
Replicate 2:
PM04A: J:\Service\Customer\300\313\data\assay\7980\ecol_SIDx5932xK12___4_37_980_1_Junx03x2010_A_02B.oka
Replicate 1:
PM05 : J:\Service\Customer\300\313\data\assay\7991\ecol_SIDx5932xK12___5_37_991_1_Junx09x2010_A_03A.oka
Replicate 2:
PM05 : J:\Service\Customer\300\313\data\assay\7980\ecol_SIDx5932xK12___5_37_980_1_Junx03x2010_A_03A.oka
Replicate 1:
PM06 : J:\Service\Customer\300\313\data\assay\7991\ecol_SIDx5932xK12___6_37_991_1_Junx09x2010_A_03B.oka
Replicate 2:
PM06 : J:\Service\Customer\300\313\data\assay\7980\ecol_SIDx5932xK12___6_37_980_1_Junx03x2010_A_03B.oka
Replicate 1:
PM07 : J:\Service\Customer\300\313\data\assay\7980\ecol_SIDx5932xK12___7_37_980_1_Junx03x2010_A_04A.oka
Replicate 2:
PM07 : J:\Service\Customer\300\313\data\assay\7991\ecol_SIDx5932xK12___7_37_991_1_Junx09x2010_A_04A.oka
Replicate 1:
PM08 : J:\Service\Customer\300\313\data\assay\7991\ecol_SIDx5932xK12___8_37_991_1_Junx09x2010_A_04B.oka
Replicate 2:
PM08 : J:\Service\Customer\300\313\data\assay\7980\ecol_SIDx5932xK12___8_37_980_1_Junx03x2010_A_04B.oka
Replicate 1:
PM09 : J:\Service\Customer\300\313\data\assay\7991\ecol_SIDx5932xK12___9_37_991_1_Junx09x2010_A_05A.oka
Replicate 2:
PM09 : J:\Service\Customer\300\313\data\assay\7980\ecol_SIDx5932xK12___9_37_980_1_Junx03x2010_A_05A.oka
Replicate 1:
PM10 : J:\Service\Customer\300\313\data\assay\7980\ecol_SIDx5932xK12___10_37_980_1_Junx03x2010_A_05B.oka
Replicate 2:
PM10 : J:\Service\Customer\300\313\data\assay\7991\ecol_SIDx5932xK12___10_37_991_1_Junx09x2010_A_05B.oka
Replicate 1:
PM11C: J:\Service\Customer\300\313\data\assay\7991\ecol_SIDx5932xK12___11_37_991_1_Junx09x2010_A_06A.oka
Replicate 2:
PM11C: J:\Service\Customer\300\313\data\assay\7980\ecol_SIDx5932xK12___11_37_980_1_Junx03x2010_A_06A.oka
Replicate 1:
PM12B: J:\Service\Customer\300\313\data\assay\7980\ecol_SIDx5932xK12___12_37_980_1_Junx03x2010_A_06B.oka
Replicate 2:
PM12B: J:\Service\Customer\300\313\data\assay\7991\ecol_SIDx5932xK12___12_37_991_1_Junx09x2010_A_06B.oka
Replicate 1:
PM13B: J:\Service\Customer\300\313\data\assay\7991\ecol_SIDx5932xK12___13_37_991_1_Junx09x2010_A_07A.oka
Replicate 2:
PM13B: J:\Service\Customer\300\313\data\assay\7980\ecol_SIDx5932xK12___13_37_980_1_Junx03x2010_A_07A.oka
Replicate 1:
PM14A: J:\Service\Customer\300\313\data\assay\7991\ecol_SIDx5932xK12___14_37_991_1_Junx09x2010_A_07B.oka
Replicate 2:
PM14A: J:\Service\Customer\300\313\data\assay\7980\ecol_SIDx5932xK12___14_37_980_1_Junx03x2010_A_07B.oka
Replicate 1:
PM15B: J:\Service\Customer\300\313\data\assay\7991\ecol_SIDx5932xK12___15_37_991_1_Junx09x2010_A_08A.oka
Replicate 2:
PM15B: J:\Service\Customer\300\313\data\assay\7980\ecol_SIDx5932xK12___15_37_980_1_Junx03x2010_A_08A.oka
Replicate 1:
PM16A: J:\Service\Customer\300\313\data\assay\7980\ecol_SIDx5932xK12___16_37_980_1_Junx03x2010_A_08B.oka
Replicate 2:
PM16A: J:\Service\Customer\300\313\data\assay\7991\ecol_SIDx5932xK12___16_37_991_1_Junx09x2010_A_08B.oka
Replicate 1:
PM17A: J:\Service\Customer\300\313\data\assay\7991\ecol_SIDx5932xK12___17_37_991_1_Junx09x2010_A_09A.oka
Replicate 2:
PM17A: J:\Service\Customer\300\313\data\assay\7980\ecol_SIDx5932xK12___17_37_980_1_Junx03x2010_A_09A.oka
Replicate 1:
PM18C: J:\Service\Customer\300\313\data\assay\7991\ecol_SIDx5932xK12___18_37_991_1_Junx09x2010_A_09B.oka
Replicate 2:
PM18C: J:\Service\Customer\300\313\data\assay\7980\ecol_SIDx5932xK12___18_37_980_1_Junx03x2010_A_09B.oka
Replicate 1:
PM19 : J:\Service\Customer\300\313\data\assay\7991\ecol_SIDx5932xK12___19_37_991_1_Junx09x2010_A_10A.oka
Replicate 2:
PM19 : J:\Service\Customer\300\313\data\assay\7980\ecol_SIDx5932xK12___19_37_980_1_Junx03x2010_A_10A.oka
Replicate 1:
PM20B: J:\Service\Customer\300\313\data\assay\7991\ecol_SIDx5932xK12___20_37_991_1_Junx09x2010_A_10B.oka
Replicate 2:
PM20B: J:\Service\Customer\300\313\data\assay\7980\ecol_SIDx5932xK12___20_37_980_1_Junx03x2010_A_10B.oka
```
