# Supplementary material for: Datasets for transcriptomics, q-proteomics and phenotype microarrays of polyphosphate metabolism mutants from Escherichia coli
Source: Data Brief. 2017 Mar 18;12:13–7. doi: 10.1016/j.dib.2017.03.010 (PMC5367803; doi:10.1016/j.dib.2017.03.010)
Supplement: Supplementary file 2 — Supplementary material [file mmc2.zip › BBAGEN-16-480/Ecoli ppx_versus_EcoliK12.doc]

```
EcolSIDx5934xppxx versus EcolSIDx5932xK12


BiOLOG
21124 Cabot Blvd.
Hayward, CA 94545


                    Phenotype MicroArray Analysis
              Test: Ecol_SID_5934_ppx-
         Reference: Ecol_SID_5932_K12
       Report Date: 6/16/2010 11:17:45 AM


Reproducibiltiy Analysis:

Test                                               Reference
PM01:    01: passes                      PM01:    01: passes
PM02:    00: passes                      PM02:    00: passes
PM03:    00: passes                      PM03:    01: passes
PM04:    00: passes                      PM04:    00: passes
PM05:    01: passes                      PM05:    00: passes
PM06:    00: passes                      PM06:    00: passes
PM07:    00: passes                      PM07:    00: passes
PM08:    00: passes                      PM08:    00: passes
PM09:    00: passes                      PM09:    00: passes
PM10:    01: passes                      PM10:    00: passes
PM11:    00: passes                      PM11:    00: passes
PM12:    00: passes                      PM12:    01: passes
PM13:    00: passes                      PM13:    00: passes
PM14:    00: passes                      PM14:    00: passes
PM15:    01: passes                      PM15:    00: passes
PM16:    00: passes                      PM16:    00: passes
PM17:    02: passes                      PM17:    02: passes
PM18:    01: passes                      PM18:    00: passes
PM19:    07: passes                      PM19:    00: passes
PM20:    01: passes                      PM20:    00: passes
PM21:    -1:    N/A                      PM21:    -1:    N/A
PM22:    -1:    N/A                      PM22:    -1:    N/A
PM23:    -1:    N/A                      PM23:    -1:    N/A
PM24:    -1:    N/A                      PM24:    -1:    N/A
PM25:    -1:    N/A                      PM25:    -1:    N/A
PM26:    -1:    N/A                      PM26:    -1:    N/A
PM27:    -1:    N/A                      PM27:    -1:    N/A
PM28:    -1:    N/A                      PM28:    -1:    N/A
PM29:    -1:    N/A                      PM29:    -1:    N/A
PM30:    -1:    N/A                      PM30:    -1:    N/A
PM31:    -1:    N/A                      PM31:    -1:    N/A
PM32:    -1:    N/A                      PM32:    -1:    N/A
PM33:    -1:    N/A                      PM33:    -1:    N/A
PM34:    -1:    N/A                      PM34:    -1:    N/A
PM35:    -1:    N/A                      PM35:    -1:    N/A
PM36:    -1:    N/A                      PM36:    -1:    N/A
PM37:    -1:    N/A                      PM37:    -1:    N/A
PM38:    -1:    N/A                      PM38:    -1:    N/A
PM39:    -1:    N/A                      PM39:    -1:    N/A
PM40:    -1:    N/A                      PM40:    -1:    N/A


Replicate 1 versus Replicate 2 of Test:
Ecol_SID_5934_ppx-
```

Replicate 1 versus Replicate 2 of Reference:

Ecol_SID_5932_K12

```
Run 1:
Ecol_SID_5934_ppx-( green )
 versus
Ecol_SID_5932_K12( red )
```

```
Run 2:
Ecol_SID_5934_ppx-( green )
 versus
Ecol_SID_5932_K12( red )
```

```
Consensus:
Ecol_SID_5934_ppx-( green )
 versus
Ecol_SID_5932_K12( red )
```

```
PM Report:
Ecol_SID_5934_ppx-_
 versus
Ecol_SID_5932_K12_

Phenotypes Gained:
PM01 	E07	  74	a-Hydroxybutyric Acid	C-Source, carboxylic acid
PM16A	E07,E08	 133	5-Azacytidine	DNA methylation, methyltransferase inhibitor
PM18C	B07	 115	Pipemidic Acid	DNA topoisomerase
PM20B	D07	  97	Ciprofloxacin	DNA topoisomerase
PM15B	A05,A06,A07	 188	Guanidine hydrochloride	membrane, chaotropic agent
PM08 	C12	 137	Pro-Asn	N-Source, peptide
PM07 	B01	 127	Lys-Pro	N-Source, peptide
PM08 	C11	 109	Pro-Arg	N-Source, peptide
PM06 	E11	 106	Gly-Pro	N-Source, peptide
PM07 	D11	 102	Pro-Pro	N-Source, peptide
PM07 	D06	  96	Pro-Gln	N-Source, peptide
PM07 	E07	  91	Ser-Pro	N-Source, peptide
PM08 	D05	  90	Pro-Trp	N-Source, peptide
PM07 	F05	  78	Thr-Pro	N-Source, peptide
PM06 	A12	  73	Ala-Pro	N-Source, peptide
PM18C	H01,H02,H03	 232	5-Fluoro-5`-deoxyuridine	nucleic acid analog, pyrimidine, DNA synthesis
PM17A	B06,B07	 131	Hygromycin B	protein synthesis, 30S ribosomal subunit, aminoglycoside
PM18C	A09,A10,A11	 191	Thiamphenicol	protein synthesis, amphenicol
PM14A	F01	  77	Chloramphenicol	protein synthesis, amphenicol
PM18C	G07	  91	Guanazole	ribonucleotide DP reductase inhibitor
PM17A	F01,F02,F03	 196	Lithium chloride	toxic cation
PM09 	H02,H03,H04,H05,H06	 540	100mM Sodium Nitrate	toxicity, nitrate

Phenotypes Lost:
PM01 	B01	 -84	D-Serine	C-Source, amino acid
PM01 	G06	 -83	Ala-Gly	C-Source, amino acid
PM01 	C06	 -87	L-Rhamnose	C-Source, carbohydrate
PM01 	B07	 -84	D,L-a-Glycerol Phosphate	C-Source, carbohydrate
PM01 	C04	 -82	D-Ribose	C-Source, carbohydrate
PM01 	A04	-114	D-Saccharic Acid	C-Source, carboxylic acid
PM01 	D06	-104	a-Ketoglutaric Acid	C-Source, carboxylic acid
PM01 	F08	 -78	Mucic Acid	C-Source, carboxylic acid
PM18C	A06,A07	-127	Pyrophosphate	chelator, hydrophilic
PM11C	E10	 -75	Nalidixic acid	DNA topoisomerase
PM12B	E02,E03	-175	2,4-Diamino-6,7-diisopropylpteridine	folate antagonist, vibriostatic agent
PM17A	A01,A02	-137	D-Serine	inhibits 3PGA dehydrogenase (L-serine and pantothenate synthesis)
PM03B	C03	 -84	D-Alanine	N-Source, amino acid
PM06 	A02	 -81	L-Glutamine	N-Source, amino acid
PM03B	F04	-127	Cytidine	N-Source, other
PM03B	F03	-122	Adenosine	N-Source, other
PM03B	E08	-110	D-Glucosamine	N-Source, other
PM03B	E11	-110	N-Acetyl-D-Glucosamine	N-Source, other
PM06 	D05	-112	Gln-Gln	N-Source, peptide
PM03B	H02	-105	Ala-Gln	N-Source, peptide
PM03B	H07	-105	Ala-Thr	N-Source, peptide
PM03B	H04	 -99	Ala-Gly	N-Source, peptide
PM07 	F06	 -98	Trp-Ala	N-Source, peptide
PM06 	D06	 -96	Gln-Gly	N-Source, peptide
PM07 	B09	 -96	Met-Gln	N-Source, peptide
PM03B	H09	 -94	Gly-Gln	N-Source, peptide
PM08 	A04	 -89	Ala-Gln	N-Source, peptide
PM06 	B03	 -86	Ala-Trp	N-Source, peptide
PM06 	B02	 -84	Ala-Thr	N-Source, peptide
PM06 	A05	 -81	Ala-Asn	N-Source, peptide
PM06 	E02	 -78	Gly-Ala	N-Source, peptide
PM07 	G06	 -78	Tyr-Gln	N-Source, peptide
PM06 	B04	 -76	Ala-Tyr	N-Source, peptide
PM06 	A07	 -75	Ala-Gly	N-Source, peptide
PM06 	A11	 -74	Ala-Phe	N-Source, peptide
PM09 	D10	-105	10% Ethylene Glycol	osmotic sensitivity, ethylene glycol
PM09 	D11	-102	15% Ethylene Glycol	osmotic sensitivity, ethylene glycol
PM19 	E09,E10	-247	Lawsone	oxidizing agent
PM04A	A11	 -79	Adenosine 2`,3`-Cyclic Monophosphate	P-Source, organic
PM14A	G02,G03	-154	Chelerythrine	protein kinase C inhibitor
PM19 	A01,A02	-164	Josamycin	protein synthesis, 50S ribosomal subunit, macrolide
PM19 	D05,D06	-161	Iodonitro tetrazolium violet	respiration
PM09 	G09	-109	100mM Ammonium Sulfate pH 8	toxicity, ammonia
PM15B	A09,A10	-136	Cefmetazole	wall, cephalosporin
PM14A	E02	 -75	Cefoxitin	wall, cephalosporin


Appendix:

         Carbon and Nutrient Max Read Hour: 024
Nitrogen, Phosphorus, Sulfur Max Read Hour: 048
                 Sensitivity Max Read Hour: 024

              Metabolic Distance Threshold: 070
            Sensitivity Distance Threshold: 070

Test Data Files:
Replicate 1:
PM01 : J:\Service\Customer\300\313\data\assay\7982\ecol_SIDx5934xppxx___1_37_982_106_Junx03x2010_A_01A.oka
Replicate 2:
PM01 : J:\Service\Customer\300\313\data\assay\7993\ecol_SIDx5934xppxx___1_37_993_106_Junx09x2010_A_01A.oka
Replicate 1:
PM02A: J:\Service\Customer\300\313\data\assay\7993\ecol_SIDx5934xppxx___2_37_993_106_Junx09x2010_A_01B.oka
Replicate 2:
PM02A: J:\Service\Customer\300\313\data\assay\7982\ecol_SIDx5934xppxx___2_37_982_106_Junx03x2010_A_01B.oka
Replicate 1:
PM03B: J:\Service\Customer\300\313\data\assay\7993\ecol_SIDx5934xppxx___3_37_993_106_Junx09x2010_A_02A.oka
Replicate 2:
PM03B: J:\Service\Customer\300\313\data\assay\7982\ecol_SIDx5934xppxx___3_37_982_106_Junx03x2010_A_02A.oka
Replicate 1:
PM04A: J:\Service\Customer\300\313\data\assay\7982\ecol_SIDx5934xppxx___4_37_982_106_Junx03x2010_A_02B.oka
Replicate 2:
PM04A: J:\Service\Customer\300\313\data\assay\7993\ecol_SIDx5934xppxx___4_37_993_106_Junx09x2010_A_02B.oka
Replicate 1:
PM05 : J:\Service\Customer\300\313\data\assay\7993\ecol_SIDx5934xppxx___5_37_993_106_Junx09x2010_A_03A.oka
Replicate 2:
PM05 : J:\Service\Customer\300\313\data\assay\7982\ecol_SIDx5934xppxx___5_37_982_106_Junx03x2010_A_03A.oka
Replicate 1:
PM06 : J:\Service\Customer\300\313\data\assay\7993\ecol_SIDx5934xppxx___6_37_993_106_Junx09x2010_A_03B.oka
Replicate 2:
PM06 : J:\Service\Customer\300\313\data\assay\7982\ecol_SIDx5934xppxx___6_37_982_106_Junx03x2010_A_03B.oka
Replicate 1:
PM07 : J:\Service\Customer\300\313\data\assay\7982\ecol_SIDx5934xppxx___7_37_982_106_Junx03x2010_A_04A.oka
Replicate 2:
PM07 : J:\Service\Customer\300\313\data\assay\7993\ecol_SIDx5934xppxx___7_37_993_106_Junx09x2010_A_04A.oka
Replicate 1:
PM08 : J:\Service\Customer\300\313\data\assay\7982\ecol_SIDx5934xppxx___8_37_982_106_Junx03x2010_A_04B.oka
Replicate 2:
PM08 : J:\Service\Customer\300\313\data\assay\7993\ecol_SIDx5934xppxx___8_37_993_106_Junx09x2010_A_04B.oka
Replicate 1:
PM09 : J:\Service\Customer\300\313\data\assay\7993\ecol_SIDx5934xppxx___9_37_993_106_Junx09x2010_A_05A.oka
Replicate 2:
PM09 : J:\Service\Customer\300\313\data\assay\7982\ecol_SIDx5934xppxx___9_37_982_106_Junx03x2010_A_05A.oka
Replicate 1:
PM10 : J:\Service\Customer\300\313\data\assay\7982\ecol_SIDx5934xppxx___10_37_982_106_Junx03x2010_A_05B.oka
Replicate 2:
PM10 : J:\Service\Customer\300\313\data\assay\7993\ecol_SIDx5934xppxx___10_37_993_106_Junx09x2010_A_05B.oka
Replicate 1:
PM11C: J:\Service\Customer\300\313\data\assay\7982\ecol_SIDx5934xppxx___11_37_982_106_Junx03x2010_A_06A.oka
Replicate 2:
PM11C: J:\Service\Customer\300\313\data\assay\7993\ecol_SIDx5934xppxx___11_37_993_106_Junx09x2010_A_06A.oka
Replicate 1:
PM12B: J:\Service\Customer\300\313\data\assay\7982\ecol_SIDx5934xppxx___12_37_982_106_Junx03x2010_A_06B.oka
Replicate 2:
PM12B: J:\Service\Customer\300\313\data\assay\7993\ecol_SIDx5934xppxx___12_37_993_106_Junx09x2010_A_06B.oka
Replicate 1:
PM13B: J:\Service\Customer\300\313\data\assay\7982\ecol_SIDx5934xppxx___13_37_982_106_Junx03x2010_A_07A.oka
Replicate 2:
PM13B: J:\Service\Customer\300\313\data\assay\7993\ecol_SIDx5934xppxx___13_37_993_106_Junx09x2010_A_07A.oka
Replicate 1:
PM14A: J:\Service\Customer\300\313\data\assay\7982\ecol_SIDx5934xppxx___14_37_982_106_Junx03x2010_A_07B.oka
Replicate 2:
PM14A: J:\Service\Customer\300\313\data\assay\7993\ecol_SIDx5934xppxx___14_37_993_106_Junx09x2010_A_07B.oka
Replicate 1:
PM15B: J:\Service\Customer\300\313\data\assay\7982\ecol_SIDx5934xppxx___15_37_982_106_Junx03x2010_A_08A.oka
Replicate 2:
PM15B: J:\Service\Customer\300\313\data\assay\7993\ecol_SIDx5934xppxx___15_37_993_106_Junx09x2010_A_08A.oka
Replicate 1:
PM16A: J:\Service\Customer\300\313\data\assay\7982\ecol_SIDx5934xppxx___16_37_982_106_Junx03x2010_A_08B.oka
Replicate 2:
PM16A: J:\Service\Customer\300\313\data\assay\7993\ecol_SIDx5934xppxx___16_37_993_106_Junx09x2010_A_08B.oka
Replicate 1:
PM17A: J:\Service\Customer\300\313\data\assay\7982\ecol_SIDx5934xppxx___17_37_982_106_Junx03x2010_A_09A.oka
Replicate 2:
PM17A: J:\Service\Customer\300\313\data\assay\7993\ecol_SIDx5934xppxx___17_37_993_106_Junx09x2010_A_09A.oka
Replicate 1:
PM18C: J:\Service\Customer\300\313\data\assay\7993\ecol_SIDx5934xppxx___18_37_993_106_Junx09x2010_A_09B.oka
Replicate 2:
PM18C: J:\Service\Customer\300\313\data\assay\7982\ecol_SIDx5934xppxx___18_37_982_106_Junx03x2010_A_09B.oka
Replicate 1:
PM19 : J:\Service\Customer\300\313\data\assay\7982\ecol_SIDx5934xppxx___19_37_982_106_Junx03x2010_A_10A.oka
Replicate 2:
PM19 : J:\Service\Customer\300\313\data\assay\7993\ecol_SIDx5934xppxx___19_37_993_106_Junx09x2010_A_10A.oka
Replicate 1:
PM20B: J:\Service\Customer\300\313\data\assay\7993\ecol_SIDx5934xppxx___20_37_993_106_Junx09x2010_A_10B.oka
Replicate 2:
PM20B: J:\Service\Customer\300\313\data\assay\7982\ecol_SIDx5934xppxx___20_37_982_106_Junx03x2010_A_10B.oka

Reference Data Files:
Replicate 1:
PM01 : J:\Service\Customer\300\313\data\assay\7991\ecol_SIDx5932xK12___1_37_991_1_Junx09x2010_A_01A.oka
Replicate 2:
PM01 : J:\Service\Customer\300\313\data\assay\7980\ecol_SIDx5932xK12___1_37_980_1_Junx03x2010_A_01A.oka
Replicate 1:
PM02A: J:\Service\Customer\300\313\data\assay\7991\ecol_SIDx5932xK12___2_37_991_1_Junx09x2010_A_01B.oka
Replicate 2:
PM02A: J:\Service\Customer\300\313\data\assay\7980\ecol_SIDx5932xK12___2_37_980_1_Junx03x2010_A_01B.oka
Replicate 1:
PM03B: J:\Service\Customer\300\313\data\assay\7991\ecol_SIDx5932xK12___3_37_991_1_Junx09x2010_A_02A.oka
Replicate 2:
PM03B: J:\Service\Customer\300\313\data\assay\7980\ecol_SIDx5932xK12___3_37_980_1_Junx03x2010_A_02A.oka
Replicate 1:
PM04A: J:\Service\Customer\300\313\data\assay\7991\ecol_SIDx5932xK12___4_37_991_1_Junx09x2010_A_02B.oka
Replicate 2:
PM04A: J:\Service\Customer\300\313\data\assay\7980\ecol_SIDx5932xK12___4_37_980_1_Junx03x2010_A_02B.oka
Replicate 1:
PM05 : J:\Service\Customer\300\313\data\assay\7991\ecol_SIDx5932xK12___5_37_991_1_Junx09x2010_A_03A.oka
Replicate 2:
PM05 : J:\Service\Customer\300\313\data\assay\7980\ecol_SIDx5932xK12___5_37_980_1_Junx03x2010_A_03A.oka
Replicate 1:
PM06 : J:\Service\Customer\300\313\data\assay\7991\ecol_SIDx5932xK12___6_37_991_1_Junx09x2010_A_03B.oka
Replicate 2:
PM06 : J:\Service\Customer\300\313\data\assay\7980\ecol_SIDx5932xK12___6_37_980_1_Junx03x2010_A_03B.oka
Replicate 1:
PM07 : J:\Service\Customer\300\313\data\assay\7980\ecol_SIDx5932xK12___7_37_980_1_Junx03x2010_A_04A.oka
Replicate 2:
PM07 : J:\Service\Customer\300\313\data\assay\7991\ecol_SIDx5932xK12___7_37_991_1_Junx09x2010_A_04A.oka
Replicate 1:
PM08 : J:\Service\Customer\300\313\data\assay\7991\ecol_SIDx5932xK12___8_37_991_1_Junx09x2010_A_04B.oka
Replicate 2:
PM08 : J:\Service\Customer\300\313\data\assay\7980\ecol_SIDx5932xK12___8_37_980_1_Junx03x2010_A_04B.oka
Replicate 1:
PM09 : J:\Service\Customer\300\313\data\assay\7991\ecol_SIDx5932xK12___9_37_991_1_Junx09x2010_A_05A.oka
Replicate 2:
PM09 : J:\Service\Customer\300\313\data\assay\7980\ecol_SIDx5932xK12___9_37_980_1_Junx03x2010_A_05A.oka
Replicate 1:
PM10 : J:\Service\Customer\300\313\data\assay\7980\ecol_SIDx5932xK12___10_37_980_1_Junx03x2010_A_05B.oka
Replicate 2:
PM10 : J:\Service\Customer\300\313\data\assay\7991\ecol_SIDx5932xK12___10_37_991_1_Junx09x2010_A_05B.oka
Replicate 1:
PM11C: J:\Service\Customer\300\313\data\assay\7991\ecol_SIDx5932xK12___11_37_991_1_Junx09x2010_A_06A.oka
Replicate 2:
PM11C: J:\Service\Customer\300\313\data\assay\7980\ecol_SIDx5932xK12___11_37_980_1_Junx03x2010_A_06A.oka
Replicate 1:
PM12B: J:\Service\Customer\300\313\data\assay\7980\ecol_SIDx5932xK12___12_37_980_1_Junx03x2010_A_06B.oka
Replicate 2:
PM12B: J:\Service\Customer\300\313\data\assay\7991\ecol_SIDx5932xK12___12_37_991_1_Junx09x2010_A_06B.oka
Replicate 1:
PM13B: J:\Service\Customer\300\313\data\assay\7991\ecol_SIDx5932xK12___13_37_991_1_Junx09x2010_A_07A.oka
Replicate 2:
PM13B: J:\Service\Customer\300\313\data\assay\7980\ecol_SIDx5932xK12___13_37_980_1_Junx03x2010_A_07A.oka
Replicate 1:
PM14A: J:\Service\Customer\300\313\data\assay\7991\ecol_SIDx5932xK12___14_37_991_1_Junx09x2010_A_07B.oka
Replicate 2:
PM14A: J:\Service\Customer\300\313\data\assay\7980\ecol_SIDx5932xK12___14_37_980_1_Junx03x2010_A_07B.oka
Replicate 1:
PM15B: J:\Service\Customer\300\313\data\assay\7991\ecol_SIDx5932xK12___15_37_991_1_Junx09x2010_A_08A.oka
Replicate 2:
PM15B: J:\Service\Customer\300\313\data\assay\7980\ecol_SIDx5932xK12___15_37_980_1_Junx03x2010_A_08A.oka
Replicate 1:
PM16A: J:\Service\Customer\300\313\data\assay\7980\ecol_SIDx5932xK12___16_37_980_1_Junx03x2010_A_08B.oka
Replicate 2:
PM16A: J:\Service\Customer\300\313\data\assay\7991\ecol_SIDx5932xK12___16_37_991_1_Junx09x2010_A_08B.oka
Replicate 1:
PM17A: J:\Service\Customer\300\313\data\assay\7991\ecol_SIDx5932xK12___17_37_991_1_Junx09x2010_A_09A.oka
Replicate 2:
PM17A: J:\Service\Customer\300\313\data\assay\7980\ecol_SIDx5932xK12___17_37_980_1_Junx03x2010_A_09A.oka
Replicate 1:
PM18C: J:\Service\Customer\300\313\data\assay\7991\ecol_SIDx5932xK12___18_37_991_1_Junx09x2010_A_09B.oka
Replicate 2:
PM18C: J:\Service\Customer\300\313\data\assay\7980\ecol_SIDx5932xK12___18_37_980_1_Junx03x2010_A_09B.oka
Replicate 1:
PM19 : J:\Service\Customer\300\313\data\assay\7991\ecol_SIDx5932xK12___19_37_991_1_Junx09x2010_A_10A.oka
Replicate 2:
PM19 : J:\Service\Customer\300\313\data\assay\7980\ecol_SIDx5932xK12___19_37_980_1_Junx03x2010_A_10A.oka
Replicate 1:
PM20B: J:\Service\Customer\300\313\data\assay\7991\ecol_SIDx5932xK12___20_37_991_1_Junx09x2010_A_10B.oka
Replicate 2:
PM20B: J:\Service\Customer\300\313\data\assay\7980\ecol_SIDx5932xK12___20_37_980_1_Junx03x2010_A_10B.oka
```
