# Supplementary material for: Datasets for transcriptomics, q-proteomics and phenotype microarrays of polyphosphate metabolism mutants from Escherichia coli
Source: Data Brief. 2017 Mar 18;12:13–7. doi: 10.1016/j.dib.2017.03.010 (PMC5367803; doi:10.1016/j.dib.2017.03.010)
Supplement: Supplementary file 5 — Raw data from phenotypic microarray experiments (.docx files). [file mmc5.zip › EcolSIDx5934xppxx versus EcolSIDx5933xppkx.doc]

```
EcolSIDx5934xppxx versus EcolSIDx5933xppkx


BiOLOG
21124 Cabot Blvd.
Hayward, CA 94545


                    Phenotype MicroArray Analysis
              Test: Ecol_SID_5934_ppx-
         Reference: Ecol_SID_5933_ppk-
       Report Date: 6/18/2010 11:21:28 AM


Reproducibiltiy Analysis:

Test                                               Reference
PM01:    01: passes                      PM01:    00: passes
PM02:    00: passes                      PM02:    00: passes
PM03:    00: passes                      PM03:    00: passes
PM04:    00: passes                      PM04:    04: passes
PM05:    01: passes                      PM05:    00: passes
PM06:    00: passes                      PM06:    00: passes
PM07:    00: passes                      PM07:    00: passes
PM08:    00: passes                      PM08:    02: passes
PM09:    00: passes                      PM09:    00: passes
PM10:    01: passes                      PM10:    00: passes
PM11:    00: passes                      PM11:    00: passes
PM12:    00: passes                      PM12:    00: passes
PM13:    00: passes                      PM13:    00: passes
PM14:    00: passes                      PM14:    00: passes
PM15:    01: passes                      PM15:    00: passes
PM16:    00: passes                      PM16:    01: passes
PM17:    02: passes                      PM17:    02: passes
PM18:    01: passes                      PM18:    00: passes
PM19:    07: passes                      PM19:    01: passes
PM20:    01: passes                      PM20:    01: passes
PM21:    -1:    N/A                      PM21:    -1:    N/A
PM22:    -1:    N/A                      PM22:    -1:    N/A
PM23:    -1:    N/A                      PM23:    -1:    N/A
PM24:    -1:    N/A                      PM24:    -1:    N/A
PM25:    -1:    N/A                      PM25:    -1:    N/A
PM26:    -1:    N/A                      PM26:    -1:    N/A
PM27:    -1:    N/A                      PM27:    -1:    N/A
PM28:    -1:    N/A                      PM28:    -1:    N/A
PM29:    -1:    N/A                      PM29:    -1:    N/A
PM30:    -1:    N/A                      PM30:    -1:    N/A
PM31:    -1:    N/A                      PM31:    -1:    N/A
PM32:    -1:    N/A                      PM32:    -1:    N/A
PM33:    -1:    N/A                      PM33:    -1:    N/A
PM34:    -1:    N/A                      PM34:    -1:    N/A
PM35:    -1:    N/A                      PM35:    -1:    N/A
PM36:    -1:    N/A                      PM36:    -1:    N/A
PM37:    -1:    N/A                      PM37:    -1:    N/A
PM38:    -1:    N/A                      PM38:    -1:    N/A
PM39:    -1:    N/A                      PM39:    -1:    N/A
PM40:    -1:    N/A                      PM40:    -1:    N/A


Replicate 1 versus Replicate 2 of Test:
Ecol_SID_5934_ppx-
```

Replicate 1 versus Replicate 2 of Reference:

Ecol_SID_5933_ppk-

```
Run 1:
Ecol_SID_5934_ppx-( green )
 versus
Ecol_SID_5933_ppk-( red )
```

```
Run 2:
Ecol_SID_5934_ppx-( green )
 versus
Ecol_SID_5933_ppk-( red )
```

```
Consensus:
Ecol_SID_5934_ppx-( green )
 versus
Ecol_SID_5933_ppk-( red )
```

```
PM Report:
Ecol_SID_5934_ppx-_
 versus
Ecol_SID_5933_ppk-_

Phenotypes Gained:
PM14A	A01,A02,A03	 180	Acriflavine	DNA intercalator, inhibits RNA synthesis
PM14A	B01,B02	 134	9-Aminoacridine	DNA intercalator, inhibits RNA synthesis
PM20B	D02,D03	 121	Proflavine	DNA intercalator, inhibits RNA synthesis
PM18C	B07	 105	Pipemidic Acid	DNA topoisomerase
PM20B	D07	  99	Ciprofloxacin	DNA topoisomerase
PM13B	A05	 112	Dequalinium	ion channel inhibitor, K+
PM15B	A05,A06,A07	 305	Guanidine hydrochloride	membrane, chaotropic agent
PM12B	F09,F10,F11,F12	 372	5-Fluoroorotic acid	nucleic acid analog, pyrimidine
PM18C	H01,H02,H03,H04	 269	5-Fluoro-5`-deoxyuridine	nucleic acid analog, pyrimidine, DNA synthesis
PM18C	B01,B02	 145	Trifluorothymidine	nucleic acid analog, pyrimidine, DNA synthesis
PM09 	D03	  94	5% Potassium Chloride	osmotic sensitivity, KCl
PM09 	D04	  78	6% Potassium Chloride	osmotic sensitivity, KCl
PM09 	F01	 108	1% Sodium Lactate	osmotic sensitivity, sodium lactate
PM10 	G11	  86	pH 9.5 + Trimethylamine-N-Oxide	pH, deaminase
PM10 	A08	  85	pH 8	pH, growth at 8
PM10 	A09	 107	pH 8.5	pH, growth at 8.5
PM19 	F07	  87	Blasticidin S	protein synthesis
PM11C	A09,A10,A11	 171	Lincomycin	protein synthesis, 50S ribosomal subunit, lincosamide
PM18C	A09,A10,A11	 277	Thiamphenicol	protein synthesis, amphenicol
PM14A	F01,F02	 207	Chloramphenicol	protein synthesis, amphenicol
PM11C	F01	  79	Chloramphenicol	protein synthesis, amphenicol
PM17A	F01,F02,F03	 269	Lithium chloride	toxic cation
PM13B	G05,G06	 209	Manganese (II) chloride	toxic cation
PM15B	H10	  77	Zinc chloride	toxic cation
PM09 	H03,H04,H05,H06	 402	100mM Sodium Nitrate	toxicity, nitrate

Phenotypes Lost:
PM02A	G02	-101	L-Alaninamide	C-Source, amide
PM01 	A08	-119	L-Proline	C-Source, amino acid
PM01 	B01	-100	D-Serine	C-Source, amino acid
PM01 	A09	 -93	D-Alanine	C-Source, amino acid
PM01 	D01	 -90	L-Asparagine	C-Source, amino acid
PM01 	G06	 -90	Ala-Gly	C-Source, amino acid
PM01 	B07	-119	D,L-a-Glycerol Phosphate	C-Source, carbohydrate
PM01 	C06	-109	L-Rhamnose	C-Source, carbohydrate
PM01 	B02	-105	D-Sorbitol	C-Source, carbohydrate
PM01 	B03	 -97	Glycerol	C-Source, carbohydrate
PM02A	B03	 -90	b-D-Allose	C-Source, carbohydrate
PM01 	C04	 -82	D-Ribose	C-Source, carbohydrate
PM01 	A04	-143	D-Saccharic Acid	C-Source, carboxylic acid
PM01 	F08	-128	Mucic Acid	C-Source, carboxylic acid
PM01 	D06	-126	a-Ketoglutaric Acid	C-Source, carboxylic acid
PM01 	F09	 -93	Glycolic Acid	C-Source, carboxylic acid
PM01 	H08	 -92	Pyruvic Acid	C-Source, carboxylic acid
PM02A	C09	 -87	b-Methyl-D-Glucuronic Acid	C-Source, carboxylic acid
PM01 	F10	 -86	Glyoxylic Acid	C-Source, carboxylic acid
PM10 	H01	-143	X-Caprylate	caprylate esterase
PM11C	C01,C02	-125	Bleomycin	DNA damage, oxidation
PM15B	D01,D02	-115	Phleomycin	DNA damage, oxidation
PM19 	C05,C06	-114	Umbelliferone	DNA intercalator
PM20B	F01	 -99	4-Hydroxycoumarin	DNA intercalator
PM17A	A01,A02	-130	D-Serine	inhibits 3PGA dehydrogenase (L-serine and pantothenate synthesis)
PM03B	C03	-162	D-Alanine	N-Source, amino acid
PM03B	B01	-157	L-Glutamine	N-Source, amino acid
PM03B	A09	-152	L-Asparagine	N-Source, amino acid
PM03B	B02	-150	Glycine	N-Source, amino acid
PM03B	A10	-140	L-Aspartic Acid	N-Source, amino acid
PM06 	A02	-139	L-Glutamine	N-Source, amino acid
PM03B	A07	-138	L-Alanine	N-Source, amino acid
PM07 	A02	-138	L-Glutamine	N-Source, amino acid
PM03B	A08	-136	L-Arginine	N-Source, amino acid
PM08 	A02	-124	L-Glutamine	N-Source, amino acid
PM03B	B08	-107	L-Phenylalanine	N-Source, amino acid
PM03B	C04	-101	D-Asparagine	N-Source, amino acid
PM03B	C12	-101	L-Ornithine	N-Source, amino acid
PM03B	B12	 -92	L-Tryptophan	N-Source, amino acid
PM03B	B11	 -91	L-Threonine	N-Source, amino acid
PM03B	C10	 -90	L-Citrulline	N-Source, amino acid
PM03B	B07	 -89	L-Methionine	N-Source, amino acid
PM03B	A02	-129	Ammonia	N-Source, inorganic
PM03B	F04	-151	Cytidine	N-Source, other
PM03B	E08	-146	D-Glucosamine	N-Source, other
PM03B	F03	-144	Adenosine	N-Source, other
PM03B	E11	-122	N-Acetyl-D-Glucosamine	N-Source, other
PM03B	F05	-110	Cytosine	N-Source, other
PM03B	E06	-105	Glucuronamide	N-Source, other
PM03B	G11	 -98	d-Amino-N-Valeric Acid	N-Source, other
PM03B	G08	 -91	g-Amino-N-Butyric Acid	N-Source, other
PM03B	D11	 -90	Putrescine	N-Source, other
PM03B	H02	-154	Ala-Gln	N-Source, peptide
PM03B	H07	-146	Ala-Thr	N-Source, peptide
PM07 	G06	-144	Tyr-Gln	N-Source, peptide
PM06 	A05	-143	Ala-Asn	N-Source, peptide
PM07 	B09	-143	Met-Gln	N-Source, peptide
PM03B	H09	-142	Gly-Gln	N-Source, peptide
PM06 	A07	-141	Ala-Gly	N-Source, peptide
PM06 	C05	-141	Arg-Tyr	N-Source, peptide
PM07 	B03	-141	Lys-Thr	N-Source, peptide
PM06 	B08	-138	Arg-Gln	N-Source, peptide
PM07 	G07	-138	Tyr-Glu	N-Source, peptide
PM03B	H08	-137	Gly-Asn	N-Source, peptide
PM06 	B04	-137	Ala-Tyr	N-Source, peptide
PM06 	H05	-137	Leu-Arg	N-Source, peptide
PM06 	H06	-136	Leu-Asp	N-Source, peptide
PM06 	B06	-134	Arg-Arg	N-Source, peptide
PM06 	C01	-134	Arg-Met	N-Source, peptide
PM06 	A11	-132	Ala-Phe	N-Source, peptide
PM08 	A03	-131	Ala-Asp	N-Source, peptide
PM03B	H03	-130	Ala-Glu	N-Source, peptide
PM03B	H05	-130	Ala-His	N-Source, peptide
PM06 	G05	-130	Ile-Gln	N-Source, peptide
PM07 	G08	-130	Tyr-Gly	N-Source, peptide
PM08 	A09	-130	Asp-Gln	N-Source, peptide
PM08 	E02	-130	Thr-Ser	N-Source, peptide
PM03B	H04	-129	Ala-Gly	N-Source, peptide
PM06 	A04	-128	Ala-Arg	N-Source, peptide
PM06 	A06	-128	Ala-Glu	N-Source, peptide
PM06 	A10	-128	Ala-Lys	N-Source, peptide
PM06 	C03	-128	Arg-Ser	N-Source, peptide
PM06 	A08	-127	Ala-His	N-Source, peptide
PM06 	D05	-127	Gln-Gln	N-Source, peptide
PM06 	E02	-127	Gly-Ala	N-Source, peptide
PM06 	E03	-127	Gly-Arg	N-Source, peptide
PM06 	F05	-127	His-Asp	N-Source, peptide
PM07 	B07	-127	Met-Arg	N-Source, peptide
PM06 	B02	-126	Ala-Thr	N-Source, peptide
PM06 	B05	-126	Arg-Ala	N-Source, peptide
PM07 	B08	-126	Met-Asp	N-Source, peptide
PM07 	C09	-126	Phe-Ala	N-Source, peptide
PM07 	E09	-125	Ser-Tyr	N-Source, peptide
PM06 	B03	-124	Ala-Trp	N-Source, peptide
PM07 	F02	-124	Thr-Gly	N-Source, peptide
PM07 	G05	-124	Tyr-Ala	N-Source, peptide
PM06 	C07	-123	Asn-Glu	N-Source, peptide
PM06 	E08	-123	Gly-Lys	N-Source, peptide
PM07 	B10	-123	Met-Glu	N-Source, peptide
PM08 	B03	-123	His-Ala	N-Source, peptide
PM06 	F03	-122	Gly-Tyr	N-Source, peptide
PM07 	F03	-120	Thr-Leu	N-Source, peptide
PM08 	D08	-120	Ser-Asp	N-Source, peptide
PM06 	B09	-119	Arg-Glu	N-Source, peptide
PM06 	G04	-119	Ile-Arg	N-Source, peptide
PM07 	E06	-119	Ser-Phe	N-Source, peptide
PM08 	B01	-119	Gly-Asp	N-Source, peptide
PM06 	H07	-118	Leu-Glu	N-Source, peptide
PM07 	A07	-118	Lys-Arg	N-Source, peptide
PM07 	F04	-118	Thr-Met	N-Source, peptide
PM07 	F07	-118	Trp-Arg	N-Source, peptide
PM06 	A03	-117	Ala-Ala	N-Source, peptide
PM06 	F01	-117	Gly-Thr	N-Source, peptide
PM06 	F06	-117	His-Gly	N-Source, peptide
PM07 	F08	-117	Trp-Asp	N-Source, peptide
PM06 	B10	-116	Arg-Ile	N-Source, peptide
PM06 	D06	-116	Gln-Gly	N-Source, peptide
PM07 	A08	-116	Lys-Glu	N-Source, peptide
PM07 	B02	-116	Lys-Ser	N-Source, peptide
PM07 	G03	-116	Trp-Trp	N-Source, peptide
PM06 	C04	-115	Arg-Trp	N-Source, peptide
PM08 	A04	-115	Ala-Gln	N-Source, peptide
PM08 	C03	-115	Met-Thr	N-Source, peptide
PM08 	H06	-115	Gly-Gly-Phe	N-Source, peptide
PM06 	G03	-114	Ile-Ala	N-Source, peptide
PM07 	E12	-114	Thr-Arg	N-Source, peptide
PM07 	F09	-114	Trp-Glu	N-Source, peptide
PM08 	C01	-114	Lys-Gly	N-Source, peptide
PM06 	B07	-113	Arg-Asp	N-Source, peptide
PM07 	B04	-113	Lys-Trp	N-Source, peptide
PM07 	C03	-113	Met-Lys	N-Source, peptide
PM07 	C07	-113	Met-Trp	N-Source, peptide
PM07 	E05	-113	Ser-Met	N-Source, peptide
PM07 	F01	-113	Thr-Glu	N-Source, peptide
PM06 	E05	-112	Gly-Gly	N-Source, peptide
PM06 	E09	-112	Gly-Met	N-Source, peptide
PM07 	B11	-112	Met-Gly	N-Source, peptide
PM07 	C02	-112	Met-Leu	N-Source, peptide
PM08 	A08	-112	Asp-Ala	N-Source, peptide
PM08 	B04	-112	His-Glu	N-Source, peptide
PM08 	C05	-112	Phe-Asp	N-Source, peptide
PM06 	C02	-111	Arg-Phe	N-Source, peptide
PM06 	E06	-111	Gly-His	N-Source, peptide
PM07 	A04	-111	Leu-Trp	N-Source, peptide
PM07 	D03	-111	Phe-Trp	N-Source, peptide
PM07 	E11	-111	Thr-Ala	N-Source, peptide
PM03B	H01	-110	Ala-Asp	N-Source, peptide
PM03B	H10	-110	Gly-Glu	N-Source, peptide
PM03B	H11	-110	Gly-Met	N-Source, peptide
PM06 	D01	-110	Asp-Phe	N-Source, peptide
PM06 	E10	-110	Gly-Phe	N-Source, peptide
PM08 	A11	-110	Glu-Ala	N-Source, peptide
PM06 	G06	-109	Ile-Gly	N-Source, peptide
PM08 	H04	-109	Gly-Gly-Ile	N-Source, peptide
PM06 	C11	-108	Asp-Leu	N-Source, peptide
PM07 	H01	-108	Tyr-Trp	N-Source, peptide
PM08 	A05	-108	Ala-Ile	N-Source, peptide
PM08 	B06	-108	Ile-Asn	N-Source, peptide
PM08 	H05	-108	Gly-Gly-Leu	N-Source, peptide
PM07 	F10	-107	Trp-Gly	N-Source, peptide
PM08 	A06	-107	Ala-Met	N-Source, peptide
PM07 	C05	-106	Met-Phe	N-Source, peptide
PM06 	F02	-105	Gly-Trp	N-Source, peptide
PM08 	H01	-105	Gly-Gly-Ala	N-Source, peptide
PM07 	C10	-104	Phe-Gly	N-Source, peptide
PM08 	B02	-104	Gly-Ile	N-Source, peptide
PM08 	B08	-104	Leu-Asn	N-Source, peptide
PM03B	H12	-103	Met-Ala	N-Source, peptide
PM06 	D02	-103	Asp-Trp	N-Source, peptide
PM07 	D05	-103	Pro-Asp	N-Source, peptide
PM06 	A09	-102	Ala-Leu	N-Source, peptide
PM07 	C01	-101	Met-Ile	N-Source, peptide
PM06 	B12	-100	Arg-Lys	N-Source, peptide
PM06 	G12	-100	Ile-Ser	N-Source, peptide
PM07 	G04	-100	Trp-Tyr	N-Source, peptide
PM06 	D10	 -98	Glu-Ser	N-Source, peptide
PM07 	A09	 -98	Lys-Ile	N-Source, peptide
PM07 	E04	 -98	Ser-Leu	N-Source, peptide
PM07 	F12	 -98	Trp-Lys	N-Source, peptide
PM07 	C11	 -97	Phe-Ile	N-Source, peptide
PM07 	E02	 -97	Ser-Gly	N-Source, peptide
PM08 	D12	 -97	Thr-Gln	N-Source, peptide
PM07 	E03	 -96	Ser-His	N-Source, peptide
PM07 	G01	 -96	Trp-Phe	N-Source, peptide
PM07 	G11	 -96	Tyr-Lys	N-Source, peptide
PM07 	A06	 -95	Lys-Ala	N-Source, peptide
PM08 	D07	 -95	Ser-Asn	N-Source, peptide
PM06 	D09	 -93	Glu-Gly	N-Source, peptide
PM08 	A10	 -93	Asp-Gly	N-Source, peptide
PM08 	C06	 -93	Phe-Glu	N-Source, peptide
PM06 	D07	 -91	Glu-Asp	N-Source, peptide
PM07 	G02	 -91	Trp-Ser	N-Source, peptide
PM06 	C09	 -90	Asp-Asp	N-Source, peptide
PM06 	D08	 -90	Glu-Glu	N-Source, peptide
PM06 	C10	 -89	Asp-Glu	N-Source, peptide
PM07 	B05	 -89	Lys-Tyr	N-Source, peptide
PM07 	F11	 -89	Trp-Leu	N-Source, peptide
PM07 	G10	 -89	Tyr-Leu	N-Source, peptide
PM08 	D10	 -89	Ser-Glu	N-Source, peptide
PM08 	D11	 -89	Thr-Asp	N-Source, peptide
PM06 	C12	 -88	Asp-Lys	N-Source, peptide
PM08 	H03	 -88	Gly-Gly-Gly	N-Source, peptide
PM06 	D12	 -86	Glu-Tyr	N-Source, peptide
PM06 	D11	 -84	Glu-Trp	N-Source, peptide
PM08 	C07	 -84	Gln-Glu	N-Source, peptide
PM07 	F06	 -82	Trp-Ala	N-Source, peptide
PM08 	B12	 -82	Lys-Asp	N-Source, peptide
PM06 	F11	 -81	His-Ser	N-Source, peptide
PM08 	C08	 -80	Phe-Met	N-Source, peptide
PM05 	B01	 -88	L-Glutamine	Nutritional supplement
PM05 	A01	 -85	Negative Control	Nutritional supplement, Negative control
PM09 	D11	 -73	15% Ethylene Glycol	osmotic sensitivity, ethylene glycol
PM09 	D10	 -71	10% Ethylene Glycol	osmotic sensitivity, ethylene glycol
PM15B	E10,E11	 -98	Methyl viologen	oxidizing agent
PM15B	F01,F02	-114	3, 4-Dimethoxybenzyl alcohol	oxidizing agent, free radical-peroxidase substrate
PM04A	A04	-146	Trimetaphosphate	P-Source, inorganic
PM04A	A03	-139	Pyrophosphate	P-Source, inorganic
PM04A	A02	-124	Phosphate	P-Source, inorganic
PM04A	B01	 -93	Thiophosphate	P-Source, inorganic
PM04A	B02	 -81	Dithiophosphate	P-Source, inorganic
PM04A	C07	-165	6-Phospho-Gluconic Acid	P-Source, organic
PM04A	A08	-151	Adenosine 2`-Monophosphate	P-Source, organic
PM04A	E09	-147	Thymidine 3`-Monophosphate	P-Source, organic
PM04A	A09	-145	Adenosine 3`-Monophosphate	P-Source, organic
PM04A	E02	-138	O-Phospho-L-Tyrosine	P-Source, organic
PM04A	B03	-135	D,L-a-Glycerol Phosphate	P-Source, organic
PM04A	B06	-133	D-2-Phospho-Glyceric Acid	P-Source, organic
PM04A	B08	-132	Guanosine 2`-Monophosphate	P-Source, organic
PM04A	D08	-132	Uridine 2`-Monophosphate	P-Source, organic
PM04A	A11	-131	Adenosine 2`,3`-Cyclic Monophosphate	P-Source, organic
PM04A	B05	-131	Carbamyl Phosphate	P-Source, organic
PM04A	C03	-131	D-Glucose-1-Phosphate	P-Source, organic
PM04A	C08	-131	Cytidine 2`-Monophosphate	P-Source, organic
PM04A	C09	-131	Cytidine 3`-Monophosphate	P-Source, organic
PM04A	B07	-130	D-3-Phospho-Glyceric Acid	P-Source, organic
PM04A	A10	-128	Adenosine 5`-Monophosphate	P-Source, organic
PM04A	B09	-127	Guanosine 3`-Monophosphate	P-Source, organic
PM04A	B04	-126	b-Glycerol Phosphate	P-Source, organic
PM04A	D04	-124	Phospho-L-Arginine	P-Source, organic
PM04A	B10	-123	Guanosine 5`-Monophosphate	P-Source, organic
PM04A	D07	-122	O-Phospho-L-Threonine	P-Source, organic
PM04A	D10	-122	Uridine 5`-Monophosphate	P-Source, organic
PM04A	C10	-120	Cytidine 5`-Monophosphate	P-Source, organic
PM04A	D05	-120	O-Phospho-D-Serine	P-Source, organic
PM04A	E01	-120	O-Phospho-D-Tyrosine	P-Source, organic
PM04A	E03	-120	Phosphocreatine	P-Source, organic
PM04A	E05	-120	O-Phosphoryl-Ethanolamine	P-Source, organic
PM04A	E10	-118	Thymidine 5`-Monophosphate	P-Source, organic
PM04A	D06	-114	O-Phospho-L-Serine	P-Source, organic
PM04A	D11	-113	Uridine 2`,3`-Cyclic Monophosphate	P-Source, organic
PM04A	C04	-111	D-Glucose-6-Phosphate	P-Source, organic
PM04A	C06	-110	D-Glucosamine-6-Phosphate	P-Source, organic
PM04A	E04	-110	Phosphoryl Choline	P-Source, organic
PM04A	B11	-108	Guanosine 2`,3`-Cyclic Monophosphate	P-Source, organic
PM04A	C11	-105	Cytidine 2`,3`-Cyclic Monophosphate	P-Source, organic
PM04A	C01	 -93	Phosphoenol Pyruvate	P-Source, organic
PM04A	A12	 -91	Adenosine 3`,5`-Cyclic Monophosphate	P-Source, organic
PM04A	C02	 -82	Phospho-Glycolic Acid	P-Source, organic
PM15B	F05,F06	-154	Oleandomycin	protein synthesis, 50S ribosomal subunit, macrolide
PM19 	D05	 -77	Iodonitro tetrazolium violet	respiration
PM19 	F09,F10	-136	Sodium caprylate	respiration, ionophore, H+
PM19 	C09,C10	-130	Cinnamic acid	respiration, ionophore, H+
PM19 	E01	 -90	FCCP	respiration, ionophore, H+
PM04A	F03	-132	Thiosulfate	S-Source, inorganic
PM04A	F02	-112	Sulfate	S-Source, inorganic
PM04A	F04	 -99	Tetrathionate	S-Source, inorganic
PM04A	F05	 -96	Thiophosphate	S-Source, inorganic
PM04A	F08	-113	D-Cysteine	S-Source, organic
PM04A	G03	-109	Cystathionine	S-Source, organic
PM04A	G04	-100	Lanthionine	S-Source, organic
PM04A	H06	 -99	Taurine	S-Source, organic
PM04A	F09	 -93	Cys-Gly	S-Source, organic
PM04A	G07	 -92	L-Methionine	S-Source, organic
PM04A	G09	 -91	Gly-Met	S-Source, organic
PM04A	F06	 -90	Dithiophosphate	S-Source, organic
PM04A	G05	 -90	Glutathione	S-Source, organic
PM04A	G08	 -87	D-Methionine	S-Source, organic
PM09 	G09	 -76	100mM Ammonium Sulfate pH 8	toxicity, ammonia
PM09 	H07,H08,H09	-224	100mM Sodium Nitrite	toxicity, nitrite
PM13B	F07,F08	-126	Glycine	wall
PM11C	A02,A03	-106	Amikacin	wall, lactam


Appendix:

         Carbon and Nutrient Max Read Hour: 024
Nitrogen, Phosphorus, Sulfur Max Read Hour: 048
                 Sensitivity Max Read Hour: 024

              Metabolic Distance Threshold: 080
            Sensitivity Distance Threshold: 060

Test Data Files:
Replicate 1:
PM01 : J:\Service\Customer\300\313\data\assay\7982\ecol_SIDx5934xppxx___1_37_982_106_Junx03x2010_A_01A.oka
Replicate 2:
PM01 : J:\Service\Customer\300\313\data\assay\7993\ecol_SIDx5934xppxx___1_37_993_106_Junx09x2010_A_01A.oka
Replicate 1:
PM02A: J:\Service\Customer\300\313\data\assay\7993\ecol_SIDx5934xppxx___2_37_993_106_Junx09x2010_A_01B.oka
Replicate 2:
PM02A: J:\Service\Customer\300\313\data\assay\7982\ecol_SIDx5934xppxx___2_37_982_106_Junx03x2010_A_01B.oka
Replicate 1:
PM03B: J:\Service\Customer\300\313\data\assay\7993\ecol_SIDx5934xppxx___3_37_993_106_Junx09x2010_A_02A.oka
Replicate 2:
PM03B: J:\Service\Customer\300\313\data\assay\7982\ecol_SIDx5934xppxx___3_37_982_106_Junx03x2010_A_02A.oka
Replicate 1:
PM04A: J:\Service\Customer\300\313\data\assay\7982\ecol_SIDx5934xppxx___4_37_982_106_Junx03x2010_A_02B.oka
Replicate 2:
PM04A: J:\Service\Customer\300\313\data\assay\7993\ecol_SIDx5934xppxx___4_37_993_106_Junx09x2010_A_02B.oka
Replicate 1:
PM05 : J:\Service\Customer\300\313\data\assay\7993\ecol_SIDx5934xppxx___5_37_993_106_Junx09x2010_A_03A.oka
Replicate 2:
PM05 : J:\Service\Customer\300\313\data\assay\7982\ecol_SIDx5934xppxx___5_37_982_106_Junx03x2010_A_03A.oka
Replicate 1:
PM06 : J:\Service\Customer\300\313\data\assay\7993\ecol_SIDx5934xppxx___6_37_993_106_Junx09x2010_A_03B.oka
Replicate 2:
PM06 : J:\Service\Customer\300\313\data\assay\7982\ecol_SIDx5934xppxx___6_37_982_106_Junx03x2010_A_03B.oka
Replicate 1:
PM07 : J:\Service\Customer\300\313\data\assay\7982\ecol_SIDx5934xppxx___7_37_982_106_Junx03x2010_A_04A.oka
Replicate 2:
PM07 : J:\Service\Customer\300\313\data\assay\7993\ecol_SIDx5934xppxx___7_37_993_106_Junx09x2010_A_04A.oka
Replicate 1:
PM08 : J:\Service\Customer\300\313\data\assay\7982\ecol_SIDx5934xppxx___8_37_982_106_Junx03x2010_A_04B.oka
Replicate 2:
PM08 : J:\Service\Customer\300\313\data\assay\7993\ecol_SIDx5934xppxx___8_37_993_106_Junx09x2010_A_04B.oka
Replicate 1:
PM09 : J:\Service\Customer\300\313\data\assay\7993\ecol_SIDx5934xppxx___9_37_993_106_Junx09x2010_A_05A.oka
Replicate 2:
PM09 : J:\Service\Customer\300\313\data\assay\7982\ecol_SIDx5934xppxx___9_37_982_106_Junx03x2010_A_05A.oka
Replicate 1:
PM10 : J:\Service\Customer\300\313\data\assay\7982\ecol_SIDx5934xppxx___10_37_982_106_Junx03x2010_A_05B.oka
Replicate 2:
PM10 : J:\Service\Customer\300\313\data\assay\7993\ecol_SIDx5934xppxx___10_37_993_106_Junx09x2010_A_05B.oka
Replicate 1:
PM11C: J:\Service\Customer\300\313\data\assay\7982\ecol_SIDx5934xppxx___11_37_982_106_Junx03x2010_A_06A.oka
Replicate 2:
PM11C: J:\Service\Customer\300\313\data\assay\7993\ecol_SIDx5934xppxx___11_37_993_106_Junx09x2010_A_06A.oka
Replicate 1:
PM12B: J:\Service\Customer\300\313\data\assay\7982\ecol_SIDx5934xppxx___12_37_982_106_Junx03x2010_A_06B.oka
Replicate 2:
PM12B: J:\Service\Customer\300\313\data\assay\7993\ecol_SIDx5934xppxx___12_37_993_106_Junx09x2010_A_06B.oka
Replicate 1:
PM13B: J:\Service\Customer\300\313\data\assay\7982\ecol_SIDx5934xppxx___13_37_982_106_Junx03x2010_A_07A.oka
Replicate 2:
PM13B: J:\Service\Customer\300\313\data\assay\7993\ecol_SIDx5934xppxx___13_37_993_106_Junx09x2010_A_07A.oka
Replicate 1:
PM14A: J:\Service\Customer\300\313\data\assay\7982\ecol_SIDx5934xppxx___14_37_982_106_Junx03x2010_A_07B.oka
Replicate 2:
PM14A: J:\Service\Customer\300\313\data\assay\7993\ecol_SIDx5934xppxx___14_37_993_106_Junx09x2010_A_07B.oka
Replicate 1:
PM15B: J:\Service\Customer\300\313\data\assay\7982\ecol_SIDx5934xppxx___15_37_982_106_Junx03x2010_A_08A.oka
Replicate 2:
PM15B: J:\Service\Customer\300\313\data\assay\7993\ecol_SIDx5934xppxx___15_37_993_106_Junx09x2010_A_08A.oka
Replicate 1:
PM16A: J:\Service\Customer\300\313\data\assay\7982\ecol_SIDx5934xppxx___16_37_982_106_Junx03x2010_A_08B.oka
Replicate 2:
PM16A: J:\Service\Customer\300\313\data\assay\7993\ecol_SIDx5934xppxx___16_37_993_106_Junx09x2010_A_08B.oka
Replicate 1:
PM17A: J:\Service\Customer\300\313\data\assay\7982\ecol_SIDx5934xppxx___17_37_982_106_Junx03x2010_A_09A.oka
Replicate 2:
PM17A: J:\Service\Customer\300\313\data\assay\7993\ecol_SIDx5934xppxx___17_37_993_106_Junx09x2010_A_09A.oka
Replicate 1:
PM18C: J:\Service\Customer\300\313\data\assay\7993\ecol_SIDx5934xppxx___18_37_993_106_Junx09x2010_A_09B.oka
Replicate 2:
PM18C: J:\Service\Customer\300\313\data\assay\7982\ecol_SIDx5934xppxx___18_37_982_106_Junx03x2010_A_09B.oka
Replicate 1:
PM19 : J:\Service\Customer\300\313\data\assay\7982\ecol_SIDx5934xppxx___19_37_982_106_Junx03x2010_A_10A.oka
Replicate 2:
PM19 : J:\Service\Customer\300\313\data\assay\7993\ecol_SIDx5934xppxx___19_37_993_106_Junx09x2010_A_10A.oka
Replicate 1:
PM20B: J:\Service\Customer\300\313\data\assay\7993\ecol_SIDx5934xppxx___20_37_993_106_Junx09x2010_A_10B.oka
Replicate 2:
PM20B: J:\Service\Customer\300\313\data\assay\7982\ecol_SIDx5934xppxx___20_37_982_106_Junx03x2010_A_10B.oka

Reference Data Files:
Replicate 1:
PM01 : J:\Service\Customer\300\313\data\assay\7981\ecol_SIDx5933xppkx___1_37_981_1_Junx03x2010_B_11A.oka
Replicate 2:
PM01 : J:\Service\Customer\300\313\data\assay\7992\ecol_SIDx5933xppkx___1_37_992_1_Junx09x2010_B_11A.oka
Replicate 1:
PM02A: J:\Service\Customer\300\313\data\assay\7992\ecol_SIDx5933xppkx___2_37_992_1_Junx09x2010_B_11B.oka
Replicate 2:
PM02A: J:\Service\Customer\300\313\data\assay\7981\ecol_SIDx5933xppkx___2_37_981_1_Junx03x2010_B_11B.oka
Replicate 1:
PM03B: J:\Service\Customer\300\313\data\assay\7981\ecol_SIDx5933xppkx___3_37_981_1_Junx03x2010_B_12A.oka
Replicate 2:
PM03B: J:\Service\Customer\300\313\data\assay\7992\ecol_SIDx5933xppkx___3_37_992_1_Junx09x2010_B_12A.oka
Replicate 1:
PM04A: J:\Service\Customer\300\313\data\assay\7992\ecol_SIDx5933xppkx___4_37_992_1_Junx09x2010_B_12B.oka
Replicate 2:
PM04A: J:\Service\Customer\300\313\data\assay\7981\ecol_SIDx5933xppkx___4_37_981_1_Junx03x2010_B_12B.oka
Replicate 1:
PM05 : J:\Service\Customer\300\313\data\assay\7981\ecol_SIDx5933xppkx___5_37_981_1_Junx03x2010_B_13A.oka
Replicate 2:
PM05 : J:\Service\Customer\300\313\data\assay\7992\ecol_SIDx5933xppkx___5_37_992_1_Junx09x2010_B_13A.oka
Replicate 1:
PM06 : J:\Service\Customer\300\313\data\assay\7992\ecol_SIDx5933xppkx___6_37_992_1_Junx09x2010_B_13B.oka
Replicate 2:
PM06 : J:\Service\Customer\300\313\data\assay\7981\ecol_SIDx5933xppkx___6_37_981_1_Junx03x2010_B_13B.oka
Replicate 1:
PM07 : J:\Service\Customer\300\313\data\assay\7992\ecol_SIDx5933xppkx___7_37_992_1_Junx09x2010_B_14A.oka
Replicate 2:
PM07 : J:\Service\Customer\300\313\data\assay\7981\ecol_SIDx5933xppkx___7_37_981_1_Junx03x2010_B_14A.oka
Replicate 1:
PM08 : J:\Service\Customer\300\313\data\assay\7992\ecol_SIDx5933xppkx___8_37_992_1_Junx09x2010_B_14B.oka
Replicate 2:
PM08 : J:\Service\Customer\300\313\data\assay\7981\ecol_SIDx5933xppkx___8_37_981_1_Junx03x2010_B_14B.oka
Replicate 1:
PM09 : J:\Service\Customer\300\313\data\assay\7992\ecol_SIDx5933xppkx___9_37_992_1_Junx09x2010_B_15A.oka
Replicate 2:
PM09 : J:\Service\Customer\300\313\data\assay\7981\ecol_SIDx5933xppkx___9_37_981_1_Junx03x2010_B_15A.oka
Replicate 1:
PM10 : J:\Service\Customer\300\313\data\assay\7981\ecol_SIDx5933xppkx___10_37_981_1_Junx03x2010_B_15B.oka
Replicate 2:
PM10 : J:\Service\Customer\300\313\data\assay\7992\ecol_SIDx5933xppkx___10_37_992_1_Junx09x2010_B_15B.oka
Replicate 1:
PM11C: J:\Service\Customer\300\313\data\assay\7992\ecol_SIDx5933xppkx___11_37_992_1_Junx09x2010_B_16A.oka
Replicate 2:
PM11C: J:\Service\Customer\300\313\data\assay\7981\ecol_SIDx5933xppkx___11_37_981_1_Junx03x2010_B_16A.oka
Replicate 1:
PM12B: J:\Service\Customer\300\313\data\assay\7981\ecol_SIDx5933xppkx___12_37_981_1_Junx03x2010_B_16B.oka
Replicate 2:
PM12B: J:\Service\Customer\300\313\data\assay\7992\ecol_SIDx5933xppkx___12_37_992_1_Junx09x2010_B_16B.oka
Replicate 1:
PM13B: J:\Service\Customer\300\313\data\assay\7992\ecol_SIDx5933xppkx___13_37_992_1_Junx09x2010_B_17A.oka
Replicate 2:
PM13B: J:\Service\Customer\300\313\data\assay\7981\ecol_SIDx5933xppkx___13_37_981_1_Junx03x2010_B_17A.oka
Replicate 1:
PM14A: J:\Service\Customer\300\313\data\assay\7992\ecol_SIDx5933xppkx___14_37_992_1_Junx09x2010_B_17B.oka
Replicate 2:
PM14A: J:\Service\Customer\300\313\data\assay\7981\ecol_SIDx5933xppkx___14_37_981_1_Junx03x2010_B_17B.oka
Replicate 1:
PM15B: J:\Service\Customer\300\313\data\assay\7992\ecol_SIDx5933xppkx___15_37_992_1_Junx09x2010_B_18A.oka
Replicate 2:
PM15B: J:\Service\Customer\300\313\data\assay\7981\ecol_SIDx5933xppkx___15_37_981_1_Junx03x2010_B_18A.oka
Replicate 1:
PM16A: J:\Service\Customer\300\313\data\assay\7981\ecol_SIDx5933xppkx___16_37_981_1_Junx03x2010_B_18B.oka
Replicate 2:
PM16A: J:\Service\Customer\300\313\data\assay\7992\ecol_SIDx5933xppkx___16_37_992_1_Junx09x2010_B_18B.oka
Replicate 1:
PM17A: J:\Service\Customer\300\313\data\assay\7981\ecol_SIDx5933xppkx___17_37_981_1_Junx03x2010_B_19A.oka
Replicate 2:
PM17A: J:\Service\Customer\300\313\data\assay\7992\ecol_SIDx5933xppkx___17_37_992_1_Junx09x2010_B_19A.oka
Replicate 1:
PM18C: J:\Service\Customer\300\313\data\assay\7981\ecol_SIDx5933xppkx___18_37_981_1_Junx03x2010_B_19B.oka
Replicate 2:
PM18C: J:\Service\Customer\300\313\data\assay\7992\ecol_SIDx5933xppkx___18_37_992_1_Junx09x2010_B_19B.oka
Replicate 1:
PM19 : J:\Service\Customer\300\313\data\assay\7992\ecol_SIDx5933xppkx___19_37_992_1_Junx09x2010_B_20A.oka
Replicate 2:
PM19 : J:\Service\Customer\300\313\data\assay\7981\ecol_SIDx5933xppkx___19_37_981_1_Junx03x2010_B_20A.oka
Replicate 1:
PM20B: J:\Service\Customer\300\313\data\assay\7981\ecol_SIDx5933xppkx___20_37_981_1_Junx03x2010_B_20B.oka
Replicate 2:
PM20B: J:\Service\Customer\300\313\data\assay\7992\ecol_SIDx5933xppkx___20_37_992_1_Junx09x2010_B_20B.oka
```
