# Supplementary material for: Datasets for transcriptomics, q-proteomics and phenotype microarrays of polyphosphate metabolism mutants from Escherichia coli
Source: Data Brief. 2017 Mar 18;12:13–7. doi: 10.1016/j.dib.2017.03.010 (PMC5367803; doi:10.1016/j.dib.2017.03.010)
Supplement: Supplementary file 5 — Raw data from phenotypic microarray experiments (.docx files). [file mmc5.zip › EcolSIDx5935xppkx_ppxx versus EcolSIDx5933xppkx.doc]

```
EcolSIDx5935xppkx/ppxx versus EcolSIDx5933xppkx


BiOLOG
21124 Cabot Blvd.
Hayward, CA 94545


                    Phenotype MicroArray Analysis
              Test: Ecol_SID_5935_ppk-/ppx-
         Reference: Ecol_SID_5933_ppk-
       Report Date: 6/18/2010 11:22:47 AM


Reproducibiltiy Analysis:

Test                                               Reference
PM01:    00: passes                      PM01:    00: passes
PM02:    00: passes                      PM02:    00: passes
PM03:    00: passes                      PM03:    00: passes
PM04:    04: passes                      PM04:    04: passes
PM05:    00: passes                      PM05:    00: passes
PM06:    00: passes                      PM06:    00: passes
PM07:    00: passes                      PM07:    00: passes
PM08:    00: passes                      PM08:    02: passes
PM09:    00: passes                      PM09:    00: passes
PM10:    00: passes                      PM10:    00: passes
PM11:    00: passes                      PM11:    00: passes
PM12:    03: passes                      PM12:    00: passes
PM13:    00: passes                      PM13:    00: passes
PM14:    00: passes                      PM14:    00: passes
PM15:    00: passes                      PM15:    00: passes
PM16:    00: passes                      PM16:    01: passes
PM17:    05: passes                      PM17:    02: passes
PM18:    02: passes                      PM18:    00: passes
PM19:    03: passes                      PM19:    01: passes
PM20:    00: passes                      PM20:    01: passes
PM21:    -1:    N/A                      PM21:    -1:    N/A
PM22:    -1:    N/A                      PM22:    -1:    N/A
PM23:    -1:    N/A                      PM23:    -1:    N/A
PM24:    -1:    N/A                      PM24:    -1:    N/A
PM25:    -1:    N/A                      PM25:    -1:    N/A
PM26:    -1:    N/A                      PM26:    -1:    N/A
PM27:    -1:    N/A                      PM27:    -1:    N/A
PM28:    -1:    N/A                      PM28:    -1:    N/A
PM29:    -1:    N/A                      PM29:    -1:    N/A
PM30:    -1:    N/A                      PM30:    -1:    N/A
PM31:    -1:    N/A                      PM31:    -1:    N/A
PM32:    -1:    N/A                      PM32:    -1:    N/A
PM33:    -1:    N/A                      PM33:    -1:    N/A
PM34:    -1:    N/A                      PM34:    -1:    N/A
PM35:    -1:    N/A                      PM35:    -1:    N/A
PM36:    -1:    N/A                      PM36:    -1:    N/A
PM37:    -1:    N/A                      PM37:    -1:    N/A
PM38:    -1:    N/A                      PM38:    -1:    N/A
PM39:    -1:    N/A                      PM39:    -1:    N/A
PM40:    -1:    N/A                      PM40:    -1:    N/A


Replicate 1 versus Replicate 2 of Test:
Ecol_SID_5935_ppk-/ppx-
```

Replicate 1 versus Replicate 2 of Reference:

Ecol_SID_5933_ppk-

```
Run 1:
Ecol_SID_5935_ppk-/ppx-( green )
 versus
Ecol_SID_5933_ppk-( red )
```

```
Run 2:
Ecol_SID_5935_ppk-/ppx-( green )
 versus
Ecol_SID_5933_ppk-( red )
```

```
Consensus:
Ecol_SID_5935_ppk-/ppx-( green )
 versus
Ecol_SID_5933_ppk-( red )
```

```
PM Report:
Ecol_SID_5935_ppk-/ppx-_
 versus
Ecol_SID_5933_ppk-_

Phenotypes Gained:
PM10 	H09	  74	X-?-D-Galactosaminide	?-D-galactosaminidase
PM10 	H08	  82	X-?-D-Glucosaminide	?-D-glucosaminidase
PM10 	H03	  72	X-?-D-Glucoside	?-D-glucosidase
PM10 	H07	  69	X-?-D-Glucuronide	?-D-glucuronidase
PM10 	H06	  64	X-?-D-Glucuronide	?-D-glucuronidase
PM10 	H10	  78	X-?-D-Mannoside	?-D-mannosidase
PM20B	C05,C06,C07	 226	Atropine	acetylcholine receptor, antagonist
PM20B	B01,B02	 127	Orphenadrine	anti-cholinergic
PM20B	F09	 117	Pridinol	anti-cholinergic
PM10 	H11	  60	X-PO4	aryl phosphatase
PM20B	B05,B06	 106	Propranolol	beta-adrenergic blocker
PM14A	H03,H04	 138	EGTA	chelator, Ca++
PM18C	A06,A07	 157	Pyrophosphate	chelator, hydrophilic
PM14A	B05	  74	Fusaric acid	chelator, lipophilic
PM17A	E05	 107	Compound 48/80	cyclic AMP phosphodiesterase inhibitor
PM18C	G09,G10,G11	 157	Myricetin	DNA & RNA synthesis, polymerase inhibitor
PM13B	A05	  71	Dequalinium	ion channel inhibitor, K+
PM18C	D09,D10	 153	Lidocaine	ion channel inhibitor, Na+, anaesthetic
PM15B	A05,A06,A07	 298	Guanidine hydrochloride	membrane, chaotropic agent
PM19 	H09	  68	Polymyxin B	membrane, cyclic peptide
PM12B	H09,H10	 113	Dodecyltrimethyl ammonium bromide	membrane, detergent, cationic
PM19 	B03	  72	Methyltrioctylammonium chloride	membrane, detergent, cationic
PM15B	E02	  63	Alexidine	membrane, electron transport, biguanide
PM20B	A01,A02	 134	Amitriptyline	membrane, transport
PM03B	A11	 193	L-Cysteine	N-Source, amino acid
PM06 	D04	 134	Cys-Gly	N-Source, peptide
PM06 	E04	  90	Gly-Cys	N-Source, peptide
PM09 	D09	  66	5% Ethylene Glycol	osmotic sensitivity, ethylene glycol
PM09 	D03	  90	5% Potassium Chloride	osmotic sensitivity, KCl
PM09 	D05	  80	2% Sodium Sulfate	osmotic sensitivity, Na2SO4
PM09 	D06	  78	3% Sodium Sulfate	osmotic sensitivity, Na2SO4
PM09 	F01	 122	1% Sodium Lactate	osmotic sensitivity, sodium lactate
PM09 	F02	  70	2% Sodium Lactate	osmotic sensitivity, sodium lactate
PM09 	G01,G02,G03	 263	200mM Sodium Phosphate pH 7	osmotic sensitivity; sodium phosphate
PM19 	E09	  97	Lawsone	oxidizing agent
PM10 	E08	 152	pH 9.5 + Glycine	pH, deaminase
PM10 	E04	 148	pH 9.5 + L-Asparagine	pH, deaminase
PM10 	E05	 146	pH 9.5 + L-Aspartic Acid	pH, deaminase
PM10 	E06	 142	pH 9.5 + L-Glutamic Acid	pH, deaminase
PM10 	G10	 141	pH 9.5 + Creatine	pH, deaminase
PM10 	G11	 139	pH 9.5 + Trimethylamine-N-Oxide	pH, deaminase
PM10 	G01	 136	pH 9.5 + Anthranilic Acid	pH, deaminase
PM10 	E03	 133	pH 9.5 + L-Arginine	pH, deaminase
PM10 	E07	 132	pH 9.5 + L-Glutamine	pH, deaminase
PM10 	F04	 132	pH 9.5 + L-Serine	pH, deaminase
PM10 	F05	 132	pH 9.5 + L-Threonine	pH, deaminase
PM10 	G12	 127	pH 9.5 + Urea	pH, deaminase
PM10 	E12	 126	pH 9.5 + L-Lysine	pH, deaminase
PM10 	F09	 124	pH 9.5 + Hydroxy-L-Proline	pH, deaminase
PM10 	F01	 122	pH 9.5 + L-Methionine	pH, deaminase
PM10 	E10	 115	pH 9.5 + L-Isoleucine	pH, deaminase
PM10 	E09	 112	pH 9.5 + L-Histidine	pH, deaminase
PM10 	F03	  99	pH 9.5 + L-Proline	pH, deaminase
PM10 	E01	 107	pH 9.5	pH, deaminase control
PM10 	B12	 105	pH 4.5 + L-Lysine	pH, decarboxylase
PM10 	A12	  94	pH 10	pH, growth at 10
PM10 	A08	 103	pH 8	pH, growth at 8
PM10 	A09	 119	pH 8.5	pH, growth at 8.5
PM10 	A10	 120	pH 9	pH, growth at 9
PM10 	A11	  91	pH 9.5	pH, growth at 9.5
PM14A	H05,H06	 181	Promethazine	phenothiazine, anti-cholinergic, anti-psychotic, sedative
PM17A	D09,D10	 141	Chlorpromazine	phenothiazine, anti-cholinergic, anti-psychotic, sedative
PM11C	A09	  62	Lincomycin	protein synthesis, 50S ribosomal subunit, lincosamide
PM20B	H09,H10	 174	Troleandomycin	protein synthesis, 50S ribosomal subunit, macrolide
PM13B	H09,H10	 117	Tylosin	protein synthesis, 50S ribosomal subunit, macrolide
PM12B	H01	  88	Spiramycin	protein synthesis, 50S ribosomal subunit, macrolide
PM14A	F01,F02	 193	Chloramphenicol	protein synthesis, amphenicol
PM18C	A09,A10	 141	Thiamphenicol	protein synthesis, amphenicol
PM11C	F01	  70	Chloramphenicol	protein synthesis, amphenicol
PM19 	H05,H06	 133	Thioglycerol	reducing agent, thiol, adenosyl methionine antagonist
PM20B	B09,B10	 230	Tetrazolium violet	respiration, uncoupler
PM15B	G05,G06	 127	Sodium azide	respiration, uncoupler
PM14A	E09,E10,E11,E12	 312	Sodium metaborate	toxic anion
PM14A	C09,C10,C11,C12	 279	Sodium cyanate	toxic anion
PM14A	G09,G10	 183	Nitrite	toxic anion
PM18C	E03	 100	Sodium metasilicate	toxic anion
PM17A	E09,E10	 118	Sodium tungstate	toxic anion, molybdate analog
PM14A	H09	  86	Sodium orthovanadate	toxic anion, PO4 analog
PM14A	D09,D10	 161	Sodium dichromate	toxic anion, SO4 analog
PM15B	H09,H10	 124	Zinc chloride	toxic cation
PM09 	H07,H08	 160	100mM Sodium Nitrite	toxicity, nitrite
PM15B	A04	  62	Procaine	wall, cephalosporin

Phenotypes Lost:
PM01 	A08	 -93	L-Proline	C-Source, amino acid
PM15B	D01,D02	-136	Phleomycin	DNA damage, oxidation
PM11C	A02,A03	-104	Amikacin	wall, lactam


Appendix:

         Carbon and Nutrient Max Read Hour: 024
Nitrogen, Phosphorus, Sulfur Max Read Hour: 048
                 Sensitivity Max Read Hour: 024

              Metabolic Distance Threshold: 080
            Sensitivity Distance Threshold: 060

Test Data Files:
Replicate 1:
PM01 : J:\Service\Customer\300\313\data\assay\7983\ecol_SIDx5935xppkxxppxx___1_37_983_106_Junx03x2010_B_11A.oka
Replicate 2:
PM01 : J:\Service\Customer\300\313\data\assay\7994\ecol_SIDx5935xppkxxxxppxx___1_37_994_106_Junx09x2010_B_11A.oka
Replicate 1:
PM02A: J:\Service\Customer\300\313\data\assay\7994\ecol_SIDx5935xppkxxxxppxx___2_37_994_106_Junx09x2010_B_11B.oka
Replicate 2:
PM02A: J:\Service\Customer\300\313\data\assay\7983\ecol_SIDx5935xppkxxppxx___2_37_983_106_Junx03x2010_B_11B.oka
Replicate 1:
PM03B: J:\Service\Customer\300\313\data\assay\7994\ecol_SIDx5935xppkxxxxppxx___3_37_994_106_Junx09x2010_B_12A.oka
Replicate 2:
PM03B: J:\Service\Customer\300\313\data\assay\7983\ecol_SIDx5935xppkxxppxx___3_37_983_106_Junx03x2010_B_12A.oka
Replicate 1:
PM04A: J:\Service\Customer\300\313\data\assay\7983\ecol_SIDx5935xppkxxppxx___4_37_983_106_Junx03x2010_B_12B.oka
Replicate 2:
PM04A: J:\Service\Customer\300\313\data\assay\7994\ecol_SIDx5935xppkxxxxppxx___4_37_994_106_Junx09x2010_B_12B.oka
Replicate 1:
PM05 : J:\Service\Customer\300\313\data\assay\7994\ecol_SIDx5935xppkxxxxppxx___5_37_994_106_Junx09x2010_B_13A.oka
Replicate 2:
PM05 : J:\Service\Customer\300\313\data\assay\7983\ecol_SIDx5935xppkxxppxx___5_37_983_106_Junx03x2010_B_13A.oka
Replicate 1:
PM06 : J:\Service\Customer\300\313\data\assay\7983\ecol_SIDx5935xppkxxppxx___6_37_983_106_Junx03x2010_B_13B.oka
Replicate 2:
PM06 : J:\Service\Customer\300\313\data\assay\7994\ecol_SIDx5935xppkxxxxppxx___6_37_994_106_Junx09x2010_B_13B.oka
Replicate 1:
PM07 : J:\Service\Customer\300\313\data\assay\7983\ecol_SIDx5935xppkxxppxx___7_37_983_106_Junx03x2010_B_14A.oka
Replicate 2:
PM07 : J:\Service\Customer\300\313\data\assay\7994\ecol_SIDx5935xppkxxxxppxx___7_37_994_106_Junx09x2010_B_14A.oka
Replicate 1:
PM08 : J:\Service\Customer\300\313\data\assay\7983\ecol_SIDx5935xppkxxppxx___8_37_983_106_Junx03x2010_B_14B.oka
Replicate 2:
PM08 : J:\Service\Customer\300\313\data\assay\7994\ecol_SIDx5935xppkxxxxppxx___8_37_994_106_Junx09x2010_B_14B.oka
Replicate 1:
PM09 : J:\Service\Customer\300\313\data\assay\7983\ecol_SIDx5935xppkxxppxx___9_37_983_106_Junx03x2010_B_15A.oka
Replicate 2:
PM09 : J:\Service\Customer\300\313\data\assay\7994\ecol_SIDx5935xppkxxxxppxx___9_37_994_106_Junx09x2010_B_15A.oka
Replicate 1:
PM10 : J:\Service\Customer\300\313\data\assay\7994\ecol_SIDx5935xppkxxxxppxx___10_37_994_106_Junx09x2010_B_15B.oka
Replicate 2:
PM10 : J:\Service\Customer\300\313\data\assay\7983\ecol_SIDx5935xppkxxppxx___10_37_983_106_Junx03x2010_B_15B.oka
Replicate 1:
PM11C: J:\Service\Customer\300\313\data\assay\7994\ecol_SIDx5935xppkxxxxppxx___11_37_994_106_Junx09x2010_B_16A.oka
Replicate 2:
PM11C: J:\Service\Customer\300\313\data\assay\7983\ecol_SIDx5935xppkxxppxx___11_37_983_106_Junx03x2010_B_16A.oka
Replicate 1:
PM12B: J:\Service\Customer\300\313\data\assay\7983\ecol_SIDx5935xppkxxppxx___12_37_983_106_Junx03x2010_B_16B.oka
Replicate 2:
PM12B: J:\Service\Customer\300\313\data\assay\7994\ecol_SIDx5935xppkxxxxppxx___12_37_994_106_Junx09x2010_B_16B.oka
Replicate 1:
PM13B: J:\Service\Customer\300\313\data\assay\7994\ecol_SIDx5935xppkxxxxppxx___13_37_994_106_Junx09x2010_B_17A.oka
Replicate 2:
PM13B: J:\Service\Customer\300\313\data\assay\7983\ecol_SIDx5935xppkxxppxx___13_37_983_106_Junx03x2010_B_17A.oka
Replicate 1:
PM14A: J:\Service\Customer\300\313\data\assay\7994\ecol_SIDx5935xppkxxxxppxx___14_37_994_106_Junx09x2010_B_17B.oka
Replicate 2:
PM14A: J:\Service\Customer\300\313\data\assay\7983\ecol_SIDx5935xppkxxppxx___14_37_983_106_Junx03x2010_B_17B.oka
Replicate 1:
PM15B: J:\Service\Customer\300\313\data\assay\7994\ecol_SIDx5935xppkxxxxppxx___15_37_994_106_Junx09x2010_B_18A.oka
Replicate 2:
PM15B: J:\Service\Customer\300\313\data\assay\7983\ecol_SIDx5935xppkxxppxx___15_37_983_106_Junx03x2010_B_18A.oka
Replicate 1:
PM16A: J:\Service\Customer\300\313\data\assay\7994\ecol_SIDx5935xppkxxxxppxx___16_37_994_106_Junx09x2010_B_18B.oka
Replicate 2:
PM16A: J:\Service\Customer\300\313\data\assay\7983\ecol_SIDx5935xppkxxppxx___16_37_983_106_Junx03x2010_B_18B.oka
Replicate 1:
PM17A: J:\Service\Customer\300\313\data\assay\7983\ecol_SIDx5935xppkxxppxx___17_37_983_106_Junx03x2010_B_19A.oka
Replicate 2:
PM17A: J:\Service\Customer\300\313\data\assay\7994\ecol_SIDx5935xppkxxxxppxx___17_37_994_106_Junx09x2010_B_19A.oka
Replicate 1:
PM18C: J:\Service\Customer\300\313\data\assay\7994\ecol_SIDx5935xppkxxxxppxx___18_37_994_106_Junx09x2010_B_19B.oka
Replicate 2:
PM18C: J:\Service\Customer\300\313\data\assay\7983\ecol_SIDx5935xppkxxppxx___18_37_983_106_Junx03x2010_B_19B.oka
Replicate 1:
PM19 : J:\Service\Customer\300\313\data\assay\7994\ecol_SIDx5935xppkxxxxppxx___19_37_994_106_Junx09x2010_B_20A.oka
Replicate 2:
PM19 : J:\Service\Customer\300\313\data\assay\7983\ecol_SIDx5935xppkxxppxx___19_37_983_106_Junx03x2010_B_20A.oka
Replicate 1:
PM20B: J:\Service\Customer\300\313\data\assay\7994\ecol_SIDx5935xppkxxxxppxx___20_37_994_106_Junx09x2010_B_20B.oka
Replicate 2:
PM20B: J:\Service\Customer\300\313\data\assay\7983\ecol_SIDx5935xppkxxppxx___20_37_983_106_Junx03x2010_B_20B.oka

Reference Data Files:
Replicate 1:
PM01 : J:\Service\Customer\300\313\data\assay\7981\ecol_SIDx5933xppkx___1_37_981_1_Junx03x2010_B_11A.oka
Replicate 2:
PM01 : J:\Service\Customer\300\313\data\assay\7992\ecol_SIDx5933xppkx___1_37_992_1_Junx09x2010_B_11A.oka
Replicate 1:
PM02A: J:\Service\Customer\300\313\data\assay\7992\ecol_SIDx5933xppkx___2_37_992_1_Junx09x2010_B_11B.oka
Replicate 2:
PM02A: J:\Service\Customer\300\313\data\assay\7981\ecol_SIDx5933xppkx___2_37_981_1_Junx03x2010_B_11B.oka
Replicate 1:
PM03B: J:\Service\Customer\300\313\data\assay\7981\ecol_SIDx5933xppkx___3_37_981_1_Junx03x2010_B_12A.oka
Replicate 2:
PM03B: J:\Service\Customer\300\313\data\assay\7992\ecol_SIDx5933xppkx___3_37_992_1_Junx09x2010_B_12A.oka
Replicate 1:
PM04A: J:\Service\Customer\300\313\data\assay\7992\ecol_SIDx5933xppkx___4_37_992_1_Junx09x2010_B_12B.oka
Replicate 2:
PM04A: J:\Service\Customer\300\313\data\assay\7981\ecol_SIDx5933xppkx___4_37_981_1_Junx03x2010_B_12B.oka
Replicate 1:
PM05 : J:\Service\Customer\300\313\data\assay\7981\ecol_SIDx5933xppkx___5_37_981_1_Junx03x2010_B_13A.oka
Replicate 2:
PM05 : J:\Service\Customer\300\313\data\assay\7992\ecol_SIDx5933xppkx___5_37_992_1_Junx09x2010_B_13A.oka
Replicate 1:
PM06 : J:\Service\Customer\300\313\data\assay\7992\ecol_SIDx5933xppkx___6_37_992_1_Junx09x2010_B_13B.oka
Replicate 2:
PM06 : J:\Service\Customer\300\313\data\assay\7981\ecol_SIDx5933xppkx___6_37_981_1_Junx03x2010_B_13B.oka
Replicate 1:
PM07 : J:\Service\Customer\300\313\data\assay\7992\ecol_SIDx5933xppkx___7_37_992_1_Junx09x2010_B_14A.oka
Replicate 2:
PM07 : J:\Service\Customer\300\313\data\assay\7981\ecol_SIDx5933xppkx___7_37_981_1_Junx03x2010_B_14A.oka
Replicate 1:
PM08 : J:\Service\Customer\300\313\data\assay\7992\ecol_SIDx5933xppkx___8_37_992_1_Junx09x2010_B_14B.oka
Replicate 2:
PM08 : J:\Service\Customer\300\313\data\assay\7981\ecol_SIDx5933xppkx___8_37_981_1_Junx03x2010_B_14B.oka
Replicate 1:
PM09 : J:\Service\Customer\300\313\data\assay\7992\ecol_SIDx5933xppkx___9_37_992_1_Junx09x2010_B_15A.oka
Replicate 2:
PM09 : J:\Service\Customer\300\313\data\assay\7981\ecol_SIDx5933xppkx___9_37_981_1_Junx03x2010_B_15A.oka
Replicate 1:
PM10 : J:\Service\Customer\300\313\data\assay\7981\ecol_SIDx5933xppkx___10_37_981_1_Junx03x2010_B_15B.oka
Replicate 2:
PM10 : J:\Service\Customer\300\313\data\assay\7992\ecol_SIDx5933xppkx___10_37_992_1_Junx09x2010_B_15B.oka
Replicate 1:
PM11C: J:\Service\Customer\300\313\data\assay\7992\ecol_SIDx5933xppkx___11_37_992_1_Junx09x2010_B_16A.oka
Replicate 2:
PM11C: J:\Service\Customer\300\313\data\assay\7981\ecol_SIDx5933xppkx___11_37_981_1_Junx03x2010_B_16A.oka
Replicate 1:
PM12B: J:\Service\Customer\300\313\data\assay\7981\ecol_SIDx5933xppkx___12_37_981_1_Junx03x2010_B_16B.oka
Replicate 2:
PM12B: J:\Service\Customer\300\313\data\assay\7992\ecol_SIDx5933xppkx___12_37_992_1_Junx09x2010_B_16B.oka
Replicate 1:
PM13B: J:\Service\Customer\300\313\data\assay\7992\ecol_SIDx5933xppkx___13_37_992_1_Junx09x2010_B_17A.oka
Replicate 2:
PM13B: J:\Service\Customer\300\313\data\assay\7981\ecol_SIDx5933xppkx___13_37_981_1_Junx03x2010_B_17A.oka
Replicate 1:
PM14A: J:\Service\Customer\300\313\data\assay\7992\ecol_SIDx5933xppkx___14_37_992_1_Junx09x2010_B_17B.oka
Replicate 2:
PM14A: J:\Service\Customer\300\313\data\assay\7981\ecol_SIDx5933xppkx___14_37_981_1_Junx03x2010_B_17B.oka
Replicate 1:
PM15B: J:\Service\Customer\300\313\data\assay\7992\ecol_SIDx5933xppkx___15_37_992_1_Junx09x2010_B_18A.oka
Replicate 2:
PM15B: J:\Service\Customer\300\313\data\assay\7981\ecol_SIDx5933xppkx___15_37_981_1_Junx03x2010_B_18A.oka
Replicate 1:
PM16A: J:\Service\Customer\300\313\data\assay\7981\ecol_SIDx5933xppkx___16_37_981_1_Junx03x2010_B_18B.oka
Replicate 2:
PM16A: J:\Service\Customer\300\313\data\assay\7992\ecol_SIDx5933xppkx___16_37_992_1_Junx09x2010_B_18B.oka
Replicate 1:
PM17A: J:\Service\Customer\300\313\data\assay\7981\ecol_SIDx5933xppkx___17_37_981_1_Junx03x2010_B_19A.oka
Replicate 2:
PM17A: J:\Service\Customer\300\313\data\assay\7992\ecol_SIDx5933xppkx___17_37_992_1_Junx09x2010_B_19A.oka
Replicate 1:
PM18C: J:\Service\Customer\300\313\data\assay\7981\ecol_SIDx5933xppkx___18_37_981_1_Junx03x2010_B_19B.oka
Replicate 2:
PM18C: J:\Service\Customer\300\313\data\assay\7992\ecol_SIDx5933xppkx___18_37_992_1_Junx09x2010_B_19B.oka
Replicate 1:
PM19 : J:\Service\Customer\300\313\data\assay\7992\ecol_SIDx5933xppkx___19_37_992_1_Junx09x2010_B_20A.oka
Replicate 2:
PM19 : J:\Service\Customer\300\313\data\assay\7981\ecol_SIDx5933xppkx___19_37_981_1_Junx03x2010_B_20A.oka
Replicate 1:
PM20B: J:\Service\Customer\300\313\data\assay\7981\ecol_SIDx5933xppkx___20_37_981_1_Junx03x2010_B_20B.oka
Replicate 2:
PM20B: J:\Service\Customer\300\313\data\assay\7992\ecol_SIDx5933xppkx___20_37_992_1_Junx09x2010_B_20B.oka
```
